# VINCIA for hadron colliders

**DOI:** 10.1140/epjc/s10052-016-4429-6

**Published:** 2016-10-28

**Authors:** N. Fischer, S. Prestel, M. Ritzmann, P. Skands

**Affiliations:** 1 School of Physics and Astronomy, Monash University, Clayton, VIC 3800 Australia; 2 SLAC National Accelerator Laboratory, Menlo Park, CA 94025 USA; 3 Nikhef, Theory Group, Science Park 105, 1098 XG Amsterdam, The Netherlands; 4 Institut de Physique Théorique, CEA Saclay, 91191 Gif-sur-Yvette Cedex, France

## Abstract

We present the first public implementation of antenna-based QCD initial- and final-state showers. The shower kernels are $2\rightarrow 3$ antenna functions, which capture not only the collinear dynamics but also the leading soft (coherent) singularities of QCD matrix elements. We define the evolution measure to be inversely proportional to the leading poles, hence gluon emissions are evolved in a $p_\perp$ measure inversely proportional to the eikonal, while processes that only contain a single pole (e.g., $g\rightarrow q\bar{q}$) are evolved in virtuality. Non-ordered emissions are allowed, suppressed by an additional power of $1/Q^2$. Recoils and kinematics are governed by exact on-shell $2\rightarrow 3$ phase-space factorisations. This first implementation is limited to massless QCD partons and colourless resonances. Tree-level matrix-element corrections are included for QCD up to $\mathcal {O}(\alpha _s^4)$ (4 jets), and for Drell–Yan and Higgs production up to $\mathcal {O}(\alpha _s^3)$ (*V* / *H* + 3 jets). The resulting algorithm has been made publicly available in Vincia  2.0.

## Introduction

The basic differential equations governing renormalisation-group-improved (resummed) perturbation theory for initial-state partons were derived in the 1970s [1–3]. The resulting DGLAP^1^ equations remain a cornerstone of high-energy phenomenology, underpinning our understanding of perturbative corrections and scaling in many contexts, in particular the structure of QCD jets, parton distribution functions, and fragmentation functions.

In the context of event generators [4], DGLAP splitting kernels are still at the heart of several present-day parton showers (including, e.g., [5–9]). Although the DGLAP kernels themselves are derived in the collinear (small-angle) limit of QCD, which is dominated by radiation off a single hard parton, the destructive-interference effects [10] which dominate for wide-angle soft-gluon emission can also be approximately accounted for in this formalism; either by choosing the shower evolution variable to be a measure of energy times angle [11] or by imposing a veto on non-angular-ordered emissions [12]. The resulting parton-shower algorithms are called *coherent*. A third alternative, increasingly popular and also adopted in this work, is to replace the parton-based DGLAP picture by so-called colour dipoles [13] (known as antennae in the context of fixed-order subtraction schemes [14–18]),^2^ which incorporate all single-unresolved (i.e., both soft and collinear) limits explicitly. In the context of shower algorithms, this approach was originally pioneered by the Ariadne program [13, 21] and is now widely used [22–30]. We note that the word “coherence” is used in different contexts, such as angular ordering. When we use coherence in the context of antenna functions, we define it at the lowest level, as follows: antenna functions sum up the radiation from two sides of the leading-$N_C$ dipole coherently, at the amplitude level; see also Ref. [27].

In addition, shower algorithms rely on several further improvements that go beyond the LO DGLAP picture, including: exact momentum conservation (related to the choice of recoil strategy), colour-flow tracing (in the leading-$N_C$ limit, related to coherence at both the perturbative and non-perturbative levels), and higher-order-improved scale choices (including the use of $\mu _R=p_\perp$ for gluon emissions and the so-called CMW scheme translation which applies in the soft limit [31, 32]). Each of these are associated with ambiguities, with Sect. 2 containing the details of our choices and motivations.

Finally, in the context of initial-state parton showers, the evolution from a high factorisation scale to a low one corresponds to an evolution in spacelike (negative) virtualities, “backwards” towards lower resolution. The correct equations for backwards parton-shower evolution were first derived by Sjöstrand [33]; in particular it is essential to multiply the evolution kernels by ratios of parton distribution functions (PDFs), to recover the correct low-scale structure of the incoming beam hadrons. We shall use a generalisation of backwards evolution to the case of simultaneous evolution of the two incoming-hadron PDFs, similar to that presented in [26].

The merits of different shower algorithms is a frequent topic of debate, with individual approaches differing by which compromises are made and by the effective higher-order terms that are generated. We emphasise the following three attractive properties of antenna showers:

- They are intrinsically coherent, in the sense that the correct eikonal structure is generated for each single-unresolved soft gluon, up to corrections suppressed by at least $1/N_C^2$. Especially for initial–final antennae, where gluon emission off initial- and final-state legs interfere, has some challenges.^3^ In the final–final case which was already testable with previous Vincia  versions, a recent OPAL study of 4-jet events [37] found good agreement between Vincia  and several recently proposed coherence-sensitive observables [38].
- They are extremely simple, relying on local and universal $2\rightarrow 3$ phase-space maps which represent an exact factorisation of the *n*-particle phase spaces not only in the soft and collinear regions but over all of phase space. This makes for highly tractable analytical expansions on which our accompanying matrix-element correction formalism is based [39]. The pure shower is in some sense merely a skeleton for generating the leading singularities, with corrections for both hard and soft emissions regarded as an intrinsic part of the formalism, restoring the emission patterns to at least LO accuracy up to the matched orders.
- There is a close correspondence with the antenna-subtraction formalism used in fixed-order calculations [16–18], which is based on the same subtraction terms and phase-space maps. This property was already utilised in [40] to implement a simple and highly efficient procedure for NLO corrections to gluon emission off a $q\bar{q}$ antenna. Highly non-trivial fixed-order results which have recently been obtained within the antenna formalism include NNLO calculations for $Z+\mathrm {jet}$ [41], $H+\mathrm {jet}$ [42] (for $m_t\rightarrow \infty$), $gg\rightarrow gg$ [43], and leading-colour $q\bar{q}\rightarrow t\bar{t}$ [44] production at hadron colliders. While it is (far) beyond the scope of the present work to connect directly with these calculations, their feasibility is encouraging to us, and provides a strong motivation for future developments of the antenna-shower formalism.

The aim with this work is to present the first full-fledged and publicly available antenna shower for hadron colliders, extending from previous work on final-state antenna showers developed in [23, 39] and building on the proof-of-concept studies for hadronic initial states reported in [29, 45]. The model is implemented in—and defines—version 2.0 of the Vincia  plug-in to the Pythia 8 event generator [34]. This article is also intended to serve as the first physics manual for Vincia  2.0. It is accompanied by a more technical HTML User Reference documenting each of the user-modifiable parameters and switches at the technical level [46] and an author’s compendium documenting more detailed algorithmic aspects [47]; both of these auxiliary documents are included with the code package, which is publicly available via the HepForge repository at http://vincia.hepforge.org.

In Sect. 2 we introduce the basic antenna-shower formalism, including our notation and conventions. We mainly focus on initial–initial and initial–final configurations and summarise final–final configurations only briefly, as a more extensive description is available in [23, 39]. Our conventions for colour flow are specified in Sect. 2.6. These are intended to maximise information on coherence while simultaneously generating a state in which all colour tags obey the index-based treatment of subleading-colour correlations proposed in [48, 49]. By assigning these indices after each branching and tracing them through the shower evolution, rather than statistically assigning them at the end of the evolution as was done in [49], we remove the risk of accidentally generating unphysical colour flows.^4^ We therefore believe the procedure proposed here represents an improvement on the one in [49]. The extension of Vincia ’s automated treatment of perturbative shower uncertainties to hadron collisions is documented in Sect. 2.7.

In Sect. 3, we present the extension of the GKS^5^ matrix-element-correction (MEC) formalism [39] to initial-state partons, starting with the case of a basic process accompanied by one or more jets whose scales are nominally harder than that of the basic process in Sect. 3.1. In Sect. 3.2, we present some basic numerical comparisons between tree-level matrix elements and our shower formalism expanded to the equivalent level (i.e., setting all Sudakov factors and coupling constants to unity), to validate that combinations of $2\rightarrow 3$ antenna branchings do produce a reasonable agreement with the full *n*-parton matrix elements. We discuss our extension of “smooth ordering” [39] to reach non-ordered parts of phase space in Sect. 3.3, again focusing on the initial-state context. Section 3.5 summarises the application of smooth ordering to the specific case of hard jets in QCD processes. In Sect. 3.6 we extend and document Vincia ’s existing use of MadGraph 4 [50] matrix elements.

The set of numerical parameters which define the default “tune” of Vincia  2.0 is documented in Sect. 4, including our preferred convention choice for $\alpha _s$, the most important parameter of any shower algorithm. A set of comparisons to a selection of salient experimentally measured distributions for hadronic *Z* decays, Drell–Yan, and QCD jet production are included to document and validate the performance of the shower algorithm with these parameters.

Finally, in Sect. 5, we summarise and give an outlook. Additional material, as referred to in the text, is collected in the Appendices.

## Vincia ’s Antenna showers

A QCD antenna represents a colour-connected parton pair which undergoes a (coherent) $2\rightarrow 3$ branching process [13–16, 51]. In contrast to conventional shower models (including both DGLAP and Catani–Seymour dipole ones) which single out one parton as the “emitter” with one (or more) other partons acting as “recoiler(s)”, the antenna formalism treats the two pre-branching “parent” partons as a single entity, with a single radiation kernel (an antenna function) driving the amount of radiation and a single “kinematics map” governing the exact relation between the pre-branching and post-branching momenta. Formally, the antenna function represents the approximate (to leading order in the vanishing invariant(s)) factorisation between the pre- and post-branching squared amplitudes, while the kinematics map encapsulates the exact on-shell factorisation of the $(n+1)$-parton phase space into the *n*-parton one and the $(2\rightarrow 3)$ antenna phase space.

Note that for branching processes involving flavour changes of the parent partons, such as $g\rightarrow q\bar{q}$, a distinction between “emitter” and “recoiler” and thus a treatment independent of the above description is possible. However, this is not compulsory and we are therefore still using the same $(2\rightarrow 3)$ antenna phase-space and kinematics map as in the case of gluon emission. Moreover, applying a $2\rightarrow 3$ branching amounts to using the lowest number of involved partons which admit an on-shell to on-shell mapping.

In this section we briefly review the notation and conventions that will be used throughout this paper (Sect. 2.1), followed by definitions for all of the phase-space convolutions or factorisations, respectively, antenna functions, and evolution variables on which Vincia ’s treatment of initial–initial, initial–final, and final–final configurations are based (Sects. 2.2, 2.3, and 2.4). The expressions for final–final configurations are unchanged relative to those in [23, 39], with the default antenna functions chosen to be those of [52] averaged over helicities. Some further details on the explicit kinematics constructions are collected in Appendix A. The explicit form of the shower-generation algorithm is presented in Sect. 2.5. Finally, we round off in Sect. 2.8 with comments on some features of earlier incarnations of Vincia  which have not (yet) been made available in Vincia  2.0.

### Notation and conventions

We use the following notation for labelling partons: capital letters for pre-branching (parent) and lower-case letters for post-branching (daughter) partons. We label incoming partons with the first letters of the alphabet, *a*, *b*, and outgoing ones with *i*, *j*, *k*. Thus, for example, a branching occurring in an initial–final antenna (a colour antenna spanned between an initial–state parton and a final–state one) would be labelled $AK \rightarrow a jk$. This is consistent with the conventions used in the most recent Vincia  papers [29, 39].^6^ The recoiler or recoiling system will be denoted by *R* and *r* respectively (compared with $R'$ and *R* in [29]).

We restrict our discussion to massless partons and denote the Lorentz-invariant momentum four-product between two partons 1 and 2 by

1 $$\begin{aligned} s_{12}~\equiv ~2p_1^{\mu }p_{2\mu }~=~(p_1+p_2)^2, \end{aligned}$$

which is always positive regardless of whether the partons involved are in the initial or final state. Momentum conservation then yields

2 $$\begin{aligned} \mathrm {FF} \,\, :\,\, s_{IK}= & {} s_{ij} + s_{jk} + s_{ik},\end{aligned}$$

3 $$\begin{aligned} \mathrm {IF} \,\, :\,\, s_{AK}= & {} s_{ak} + s_{aj} - s_{jk},\end{aligned}$$

4 $$\begin{aligned} \mathrm {II} \,\, :\,\, s_{AB}= & {} s_{ab} - s_{aj} - s_{jb}, \end{aligned}$$

for final–final (FF), initial–final (IF), and initial–initial (II) branchings, respectively.

The evolution variable, which we denote *t*, is evaluated on the post-branching partons, hence, e.g., $t_{\mathrm {FF}}=t(s_{ij},s_{jk})$. It serves as a dynamic factorisation scale for the shower, separating resolved from unresolved regions. As such, it must vanish for singular configurations. Generally, we define the evolution variable for each branching type to vanish with the same power of the momentum invariants as the leading poles of the corresponding antenna functions, see below. The complementary phase-space variable will be denoted $\zeta$.

**Colour Factors** $\mathcal {C}$ We use the following convention: for gluon emission the colour factors are $\mathcal {C}=C_A=3$ for gluon-only antennae, $\mathcal {C}=2\,C_F=8/3$ for quark-only antennae, and the mean, $\mathcal {C}=(C_A+2C_F)/2$, for quark-gluon antennae. For gluon splitting the colour factor is $\mathcal {C}=2\,T_R=1$. Note that symmetry factors, taking into account that gluons contribute to two antennae, are included in the antenna functions.

**Fig. 1:**
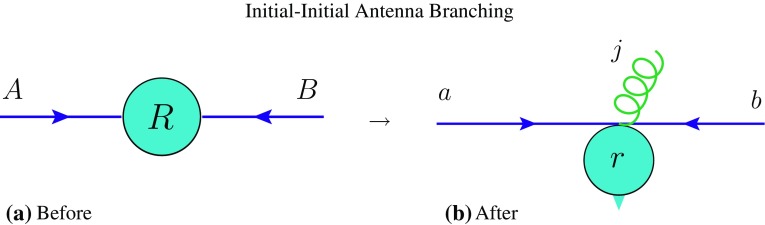
Illustration of pre-branching (*left*) and post-branching (*right*) on-shell momenta, for an initial–initial antenna branching, emphasising the transverse kick imparted to the hard system, *R*, which consists of all particles produced in the collision $A+B \rightarrow R$. The hard system is treated as a rigid body (i.e., any internal invariants are not modified) by the branching. It is subjected to a single overall Lorentz transformation, $R\rightarrow r$, equivalent to a frame reinterpretation required to orient the new incoming partons along the *z* axis. Note that we define our kinematics maps to preserve not only the invariant mass but also the rapidity of the recoiling system: $m_r^2 = m_R^2$ and $y_r = y_R$, cf. Appendix A.1

**Shower Basics** A shower algorithm is based on the probability that no branching occurs between two scales $t_{n}$ and $t_{n+1}$, with $t_{n}>t_{n+1}$. (For an introduction to conventional showers, see, e.g., [53, Chp. 40] or [4]. For antenna showers more specifically, see [39, 40]). In the case of initial-state radiation in the antenna picture the no-emission probability is

5 $$\begin{aligned} \Pi _n(t_{n},t_{n+1})= & {} \exp \left( -\sum _i \in \{n\rightarrow n+1\}\right. \nonumber \\&\left. \int _{t_{n+1}}^{t_{n}} \,\,\, \text {d}\Phi _{\text {ant}}\, 4\pi \alpha _s(t)\, \mathcal {C}\,{\bar{a}}_i(t,\zeta )\,R_{\text {pdf}\,i} \right) , \end{aligned}$$

with the colour- and coupling-stripped antenna function ${\bar{a}}$ and the (double) ratio of PDFs,

6 $$\begin{aligned} R_{\text {pdf}} = \frac{f_a(x_a,t)}{f_A(x_A,t)}\frac{f_b(x_b,t)}{f_B(x_B,t)}. \end{aligned}$$

Note that although the integral over $\text {d}\Phi _\text {ant}$ in Eq. (5) is three-dimensional, we only explicitly wrote down the boundaries in the evolution variable *t*, with integration over the complementary invariant, $\zeta$, and over the azimuth angle, $\phi$, implied. Given specific choices for *t* and $\zeta$ as functions of the phase-space invariants, the boundaries of the $\zeta$ integral are derived from energy-momentum conservation, as usual for shower algorithms (see, [4, 23, 47, 54]). This generates modifications to the LL structure which—since (*E*, *p*) conservation is a genuine physical effect—is expected to improve the shower approximation at the subleading level. (We are not aware of a rigorous proof of this statement, however.)

The sum in Eq. (5) runs over all possible $(n+1)$-parton states that can be created from the *n*-parton state, and will be implicit from here on. $\text {d}\Phi _{\text {ant}}$ is the antenna phase space, providing a mapping from two to three on-shell partons while preserving energy and momentum. The specific form for the two configurations, initial–initial and initial–final, are defined below, along with the specific forms of the evolution variable.

We define the Sudakov factor as

7 $$\begin{aligned} \Delta _n(t_{n},t_{n+1})= & {} \exp \left( -\sum _{i \in \{n\rightarrow n+1\}} \int _{t_{n+1}}^{t_{n}} \!\!\! \text {d}\Phi _{\text {ant}}\,\frac{x_A\,x_B}{x_a\,x_b}\,\right. \nonumber \\&\left. 4\pi \alpha _s(t)\,\mathcal {C}\,{\bar{a}}_i(t,\zeta )\right) . \end{aligned}$$

This object does not depend on parton distribution functions or other non-perturbative input and may thus be regarded as a purely perturbative object. Following the arguments of [30], we *define* the no-emission probability in terms of the Sudakov factor, as follows (generalised from [55]):

8 $$\begin{aligned} \Pi _n(t_{n},t_{n+1})=\frac{f_A(x_A,t_{n+1})}{f_A(x_A,t_{n})} \frac{f_B(x_B,t_{n+1})}{f_B(x_B,t_{n})}\Delta _n(t_{n},t_{n+1}). \end{aligned}$$

This in turn implicitly defines the evolution equation for the antenna shower, which, as shown in [30], is consistent with the DGLAP equation, provided the antenna functions used in Vincia  have the correct (AP-kernel) behaviour close to $z=1$, where *z* is an energy-sharing variable.^7^ This is shown in Appendix A.2, in which the collinear limits of all antenna functions used in this work are given. Note that a similar strategy of using Eq. (8) as a definition was also used when defining perturbative states in [58]. For final–final configurations, Eq. (8) simplifies to $\Pi _n(t_{n},t_{n+1})=\Delta _n(t_{n},t_{n+1})$.

### Initial–initial configurations

We denote the pre- and post-branching partons participating in an initial–initial branching by $AB\rightarrow abj$ and the (system of) particles produced by the collision by $R\rightarrow r$, cf. the illustrations in Fig. 1. In the following, we specify the phase-space convolution, antenna functions, evolution variables and the resulting no-emission probability.

**Fig. 2:**
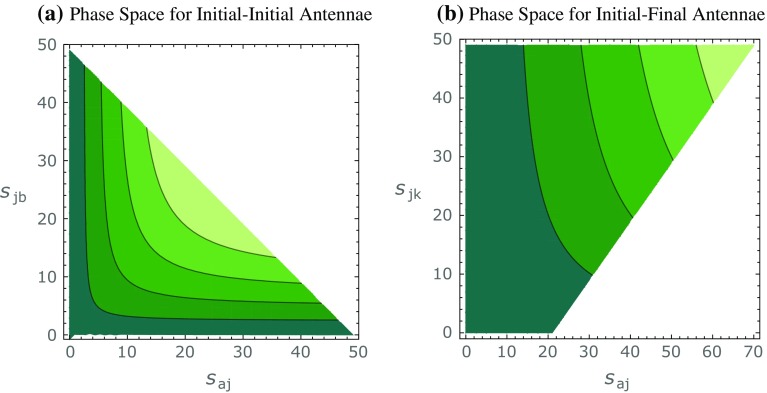
Contours of constant gluon-emission evolution variable for **a** initial–initial and **b** initial–final configurations. For **a** the recoiler is chosen to be a Higgs boson, $s_{AB}=m_H^2$, and for **b** $s_{AK}=10500\,\text {GeV}^2$ and $x_A=0.3$. For both cases, the total hadronic $\sqrt{s}=7\,\text {TeV}$

**Phase space** The phase-space convolution reads

9 $$\begin{aligned}&\int \frac{\text {d}x_a}{x_a}\Theta (1{-}x_a)\,\frac{\text {d}x_b}{x_b}\Theta (1-x_b)\, \text {d}\Phi _2(p_a,p_b\rightarrow p_j,p_r)\nonumber \\&=\int \frac{\text {d}x_A}{x_A}\Theta (1{-}x_A)\,\frac{\text {d}x_B}{x_B}\Theta (1{-}x_B) \text {d}\Phi _1(p_A,p_B{\rightarrow } p_R)\,\text {d}\Phi _{\text {ant}}^{\text {II}}\nonumber \\ \end{aligned}$$

with the antenna phase space

10 $$\begin{aligned} \text {d}\Phi _{\text {ant}}^{\text {II}} = \frac{1}{16\pi ^2}\,\frac{s_{AB}}{s_{ab}^2}\, \Theta (x_a-x_A)\,\Theta (x_b-x_B)\, \text {d}s_{aj}\,\text {d}s_{jb}\,\frac{\text {d}\phi }{2\pi }. \end{aligned}$$

See Appendix A.1 for the explicit construction of the post-branching momenta.

**Antenna functions** The gluon-emission antenna functions are

11 $$\begin{aligned} {\bar{a}}_{q\bar{q}\,g}^{\text {II}}&={\bar{a}}(a_q,b_{\bar{q}},j_g) = \frac{1}{s_{AB}}\left( 2\,\frac{s_{ab}s_{AB}}{s_{aj}s_{jb}} + \frac{s_{jb}}{s_{aj}}+ \frac{s_{aj}}{s_{jb}}\right) , \end{aligned}$$

12 $$\begin{aligned} \bar{a}_{gg\,g}^{\text {II}}&={\bar{a}}(a_g,b_g,j_g)\nonumber \\&= \frac{1}{s_{AB}}\left( 2\,\frac{s_{ab}s_{AB}}{s_{aj}s_{jb}} + 2\,\frac{s_{jb}}{s_{aj}}\frac{s_{ab}}{s_{AB}}+2\,\frac{s_{jb}}{s_{aj}}\frac{s_{AB}}{s_{ab}+s_{aj}}\right. \nonumber \\&\quad + \left. 2\,\frac{s_{aj}}{s_{jb}}\frac{s_{ab}}{s_{AB}}+2\,\frac{s_{aj}}{s_{jb}}\frac{s_{AB}}{s_{ab}+s_{jb}}\right) , \end{aligned}$$

13 $$\begin{aligned} {\bar{a}}_{qg\,g}^{\text {II}}&={\bar{a}}(a_q,b_g,j_g) =~ \frac{1}{s_{AB}}\left( 2\,\frac{s_{ab}s_{AB}}{s_{aj}s_{jb}} + \frac{s_{jb}}{s_{aj}}\right. \nonumber \\&\quad \left. +2\,\frac{s_{aj}}{s_{jb}}\frac{s_{ab}}{s_{AB}}+2\,\frac{s_{aj}}{s_{jb}}\frac{s_{AB}}{s_{ab}+s_{jb}}\right) . \end{aligned}$$

The antenna function for a gluon evolving backwards to a quark (and similarly to an antiquark) is

14 $$\begin{aligned} {\bar{a}}_{qx\,q}^{\text {II}}={\bar{a}}(a_q,b_x,j_q) =&~ \frac{1}{2s_{aj}}\frac{s_{jb}^2+s_{ab}^2}{s_{AB}^2}, \end{aligned}$$

and for a quark evolving backwards into a gluon

15 $$\begin{aligned} {\bar{a}}_{gx\,\bar{q}}^{\text {II}}={\bar{a}}(a_g,b_x,j_{\bar{q}}) =&~ \frac{1}{s_{AB}}\left( -2\,\frac{s_{jb}s_{AB}}{s_{aj}(s_{ab}-s_{aj})}+\frac{s_{ab}}{s_{aj}}\right) . \end{aligned}$$

In Appendix A.2 we show that the antenna functions correctly reproduce the DGLAP splitting kernels in the collinear limit.

**Evolution variables** We evolve gluon emission in the physical transverse momentum of the emission (relative to the $p_a$–$p_b$–axis),

16 $$\begin{aligned} t_{\text {II}}^{\text {emit}} = p_{\perp \,{\text {II}}}^2=\frac{s_{aj}s_{jb}}{s_{ab}}, \end{aligned}$$

which exhibits the same “antenna-like” $a\leftrightarrow b$ symmetry as the leading (double) poles of the corresponding antenna functions, Eqs. (11)–(13) above. The upper phase-space limit for this variable is $p_{\perp \,{\text {II}}}^2\le (s-s_{AB})^2/(4\,s)$, where *s* denotes the hadronic centre-of-mass energy squared.

Figure 2a shows constant contours of $p_{\perp \,\text {II}}^2$, as a function of the two branching invariants $s_{aj}$ and $s_{jb}$. As the phase space is symmetric in $s_{aj}$ and $s_{jb}$ it has a triangular shape whose hypotenuse is defined by the upper phase-space bound $s_{AB}+s_{aj}+s_{jb}\le s$. For branchings with flavour changes in the initial state (gluon evolving backwards to a quark or vice versa) for which the antenna functions only contain single poles, cf. Eqs. (14)–(15) above, we use the corresponding invariant, $s_{aj}$ or $s_{jb}$, respectively,

17 $$\begin{aligned} t_\text {II}^\text {conv} = Q^2_\text {II} = \left\{ \begin{array}{cl} s_{aj}&{} \text{ for } ~a ~\text{ converting } \text{ to/from } \text{ a } \text{ gluon }\\ s_{jb}&{} \text{ for } ~b ~\text{ converting } \text{ to/from } \text{ a } \text{ gluon } \end{array} \right. , \end{aligned}$$

where the phase-space limit is $s_{xj}\le s-s_{AB}$. Note that the conversion measure is equivalent to the Mandelstam |*t*| variable for the relevant diagrams. Since only one parton can convert at a time—either *A* *or* *B*—these diagrams are unique, with no interferences, as is also reflected by the corresponding antenna functions containing only single (collinear) poles.

**Fig. 3:**
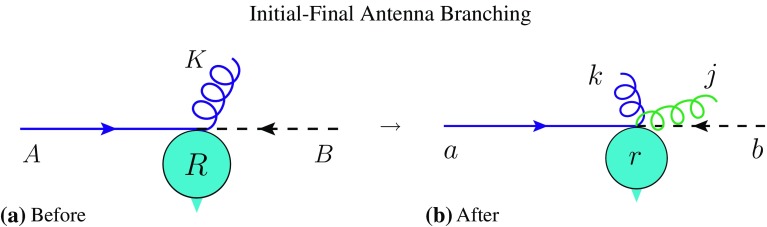
Illustration of pre-branching (*left*) and post-branching (*right*) on-shell momenta, for an initial–final (IF) antenna branching, emphasising that the momenta of the spectators *B* and *R* are unchanged: $p_b=p_B$ and $p_r = p_R$, cf. Appendix A.1

**No-emission probability** With the definitions given above (5) for initial–initial configurations reads

18 $$\begin{aligned} \Pi _n(t_{n},t_{n+1})= & {} \exp \left( - \int _{{s_{aj}}_{n+1}}^{{s_{aj}}_{n}}\!\!\!\!\!\!\!\!\!\text {d}s_{aj}\,\Theta (x_a-x_A)\,\right. \nonumber \\&\times \,\left. \int _{{s_{jb}}_{n+1}}^{{s_{jb}}_{n}}\!\!\!\!\!\!\!\!\!\text {d}s_{jb}\,\Theta (x_b-x_B)\, \int _0^{2\pi }\!\frac{\text {d}\phi }{2\pi }\, \right. \nonumber \\&\times \,\left. \frac{\alpha _s(t)}{4\pi }\,\frac{s_{AB}}{s_{ab}^2}\,\mathcal {C}\, {\bar{a}}(s_{aj},s_{jb},s_{AB})\, \frac{f_a(x_a,t)}{f_A(x_A,t)}\right. \nonumber \\&\times \,\left. \frac{f_b(x_b,t)}{f_B(x_B,t)} \right) , \end{aligned}$$

with $t=t_\text {II}(s_{aj},s_{jb},s_{ab})$. The subscript of the $s_{aj}$ and $s_{jb}$ integration limits indicates the association with the branching scales $t_n$ and $t_{n+1}$, respectively.

### Initial–final configurations

In traditional (DGLAP-based) parton-shower formulations, the radiation emitted by a colour line flowing from the initial to the final state is handled by two separate algorithms, one for ISR and one for FSR. Coherence can still be imposed by letting these algorithms share information on the angles between colour-connected partons and limiting radiation to the corresponding coherent radiation cones. But even so, several subtleties can arise in the context of specific processes or corners of phase space. Examples of problems encountered in the literature involving Pythia’s $p_\perp$-ordered showers include how radiation in dipoles stretched to the beam remnant is treated [59], whether the combined ISR+FSR evolution is interleaved or not [60] and whether/how coherence is imposed on the first emission [36].

In the context of antenna showers, the radiation off initial–final (IF) colour flows is generated by IF antennae, which are coherent ab initio. We therefore expect the treatment of wide-angle radiation to be more reliable and plagued by fewer subtleties. The main issue one faces instead is technical. Denoting the pre- and post-branching partons participating in an IF branching by $AK\rightarrow akj$, the choice of kinematics map specifying the global orientation of the *akj* system with respect to the *AK* one is equivalent to specifying the Lorentz transformation that connects the pre-branching frame, in which *A* is incoming along the *z* axis with momentum fraction $x_A$, to the post-branching one, in which *a* is incoming along the *z* axis with momentum fraction $x_a$. For a general choice of kinematics map, this can result in boosted angles entering in the relation between $x_a$ and the branching invariants, producing highly non-trivial expressions, and the phase-space boundaries can likewise become very complicated. To retain a simple structure for this first implementation, and since we anyway intend our shower as a baseline to be improved upon with matrix-element corrections, the algorithm we present in this paper is based on the simplest possible kinematics map, in which momentum is conserved locally within the antenna, $p_a - p_j - p_k = p_A - p_K$. This implies that the momentum of the hard system, *R*, is left unchanged, meaning IF branchings doe not produce a transverse recoil in the hard system. This is indicated by the unchanged momenta of the other incoming parton *B* and the final-state *R*, cf. the illustrations in Fig. 3.

**Fig. 4:**
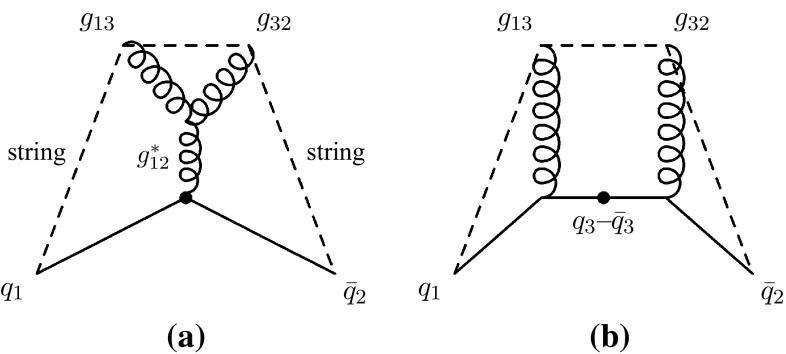
Illustration of *colour* flow in $Z\rightarrow qgg\bar{q}$, using subscripts to denote *colour indices*. Note that both *x* and *y* axes illustrate spatial dimensions, with time indicated roughly by the distance from the location of the original *Z*, denoted by *bullet symbol*. Two Feynman diagrams contribute to the same leading-colour string topology

Though we do perceive of this as artificial (e.g., a parton emitting near-collinear radiation will only generate recoil to the hard system if its colour partner happens to be in the initial state) and presumably a weak point of the physics generated by the IF algorithm [61], it is nevertheless worth pointing out that:

- Even in cases where there is only one original II antenna (as e.g., in Drell–Yan), it is not true that recoil can only be generated by the first emission. In particular, if the first branching is a (sea) quark evolving backwards to a gluon, that gluon will participate in a new II antenna, which will generate added recoil according to the above prescription for the II case. For cases with more than one II antenna (e.g., $gg\rightarrow H$), the number of possible $p_\perp$ kicks of course increases accordingly.
- In Vincia   matrix-element corrections (MECs) are regarded as an integral component of the evolution. Up to the first several orders (typically three powers of $\alpha _s$) we therefore expect to be able to apply MECs which will change the relative weighting of branching events in phase space, emphasising those regions which would have benefited most from large recoils and de-emphasising complementary ones. Matrix-element corrections will ensure that the emission pattern is correctly described with fixed-order precision. The all-orders resummation of non-LL configurations (e.g., configurations with balancing soft emissions), is, however, not formally improved, meaning a residual effect of the recoil strategy remains. Note that the MECs will nonetheless attribute a sensible lowest-order weight to hard configurations that are usually out of reach of strongly ordered parton showers.
- As already pointed out above and illustrated by [29, Figs. 3, 4], the IF radiation patterns remain *coherent*, in the sense that large colour opening angles are a prerequisite for wide-angle radiation. This is a non-trivial and important property of the antenna-shower formalism, which is preserved independently of the recoil strategy.

Given these arguments, we regard the maintained simplicity of the resulting formalism as the primary goal at this stage, which has the added benefit of producing faster, more efficient algorithms. For completeness, we note that the strategy adopted in [27] for “finite recoils” would not be applicable to Vincia  since it does not cover all of phase space and hence could not be used as the starting point for our matrix-element correction strategy.

In the following, we describe the phase-space convolution, antenna functions and resulting no-emission probability used for initial–final evolution.

**Phase space** The phase-space convolution reads

19 $$\begin{aligned}&\int \frac{\text {d}x_a}{x_a}\Theta (1-x_a)\,\frac{\text {d}x_B}{x_B}\,\Theta (1-x_B)\, \text {d}\Phi _3(p_a,\nonumber \\&\quad p_B\rightarrow p_R,p_j,p_k) \nonumber \\&= \int \frac{\text {d}x_A}{x_A}\Theta (1-x_A)\,\frac{\text {d}x_B}{x_B}\,\Theta (1-x_B)\, \text {d}\Phi _2(p_A,\nonumber \\&\quad p_B\rightarrow p_R,p_K)\,\text {d}\Phi _\text {ant}^\text {IF} \end{aligned}$$

with the antenna phase space

20 $$\begin{aligned} \text {d}\Phi _\text {ant}^\text {IF} = \frac{1}{16\pi ^2}\,\frac{s_{AK}}{(s_{AK}+s_{jk})^2}\, \Theta (x_a-x_A)\, \text {d}s_{aj}\,\text {d}s_{jk}\,\frac{\text {d}\phi }{2\pi }. \end{aligned}$$

See Appendix A.1 for the explicit construction of the post-branching momenta.

**Antenna functions** The gluon-emission antenna functions are

21 $$\begin{aligned} {\bar{a}}_{qq\,g}^\text {IF}&={\bar{a}}(a_q,k_q,j_g) = \frac{1}{s_{AK}}\left( 2\,\frac{s_{ak}s_{AK}}{s_{aj}s_{jk}} + \frac{s_{jk}}{s_{aj}}+ \frac{s_{aj}}{s_{jk}}\right) , \end{aligned}$$

22 $$\begin{aligned} {\bar{a}}_{gg\,g}^\text {IF}&=\bar{a}(a_g,k_g,j_g) = \frac{1}{s_{AK}}\left( 2\,\frac{s_{ak}s_{AK}}{s_{aj}s_{jk}} + 2\,\frac{s_{jk}}{s_{aj}}\frac{s_{ak}}{s_{AK}}\right. \nonumber \\&\left. \quad +\, 2\,\frac{s_{jk}s_{AK}}{s_{aj}(s_{AK}+s_{jk})} + \frac{s_{aj}}{s_{jk}}\frac{s_{ak}}{s_{AK}}\right) , \end{aligned}$$

23 $$\begin{aligned} \bar{a}_{qg\,g}^\text {IF}&={\bar{a}}(a_q,k_g,j_g)\nonumber \\&= \frac{1}{s_{AK}}\left( 2\,\frac{s_{ak}s_{AK}}{s_{aj}s_{jk}} + \frac{s_{jk}}{s_{aj}}+ \frac{s_{aj}}{s_{jk}}\frac{s_{ak}}{s_{AK}}\right) ,\end{aligned}$$

24 $$\begin{aligned} \bar{a}_{gq\,g}^\text {IF}&={\bar{a}}(a_g,k_q,j_g) = \frac{1}{s_{AK}}\left( 2\,\frac{s_{ak}s_{AK}}{s_{aj}s_{jk}} + 2\,\frac{s_{jk}}{s_{aj}}\frac{s_{ak}}{s_{AK}}\right. \nonumber \\&\left. \quad +\, 2\,\frac{s_{jk}s_{AK}}{s_{aj}(s_{AK}+s_{jk})} + \frac{s_{aj}}{s_{jk}}\right) . \end{aligned}$$

The antenna function for a gluon evolving backwards to a quark (and similarly to an antiquark) is

25 $$\begin{aligned} {\bar{a}}_{qx\,q}^\text {IF}={\bar{a}}(a_q,k_x,j_q)= & {} \frac{1}{2s_{aj}}\frac{s_{jk}^2+s_{ak}^2}{s_{AK}^2}, \end{aligned}$$

for a quark evolving backwards to a gluon

26 $$\begin{aligned} {\bar{a}}_{gx\,\bar{q}}^\text {IF}= & {} {\bar{a}}(a_g,k_x,j_{\bar{q}})\nonumber \\= & {} \frac{1}{s_{AK}}\left( -2\,\frac{s_{jk}(s_{AK}-s_{aj})}{s_{aj}(s_{AK}+s_{jk})}+\frac{s_{ak}}{s_{aj}}\right) ,\nonumber \\ \end{aligned}$$

and for a final-state gluon splitting

27 $$\begin{aligned} {\bar{a}}_{xq\,\bar{q}}^\text {IF}={\bar{a}}(a_x,k_q,j_{\bar{q}})&= \frac{1}{2s_{jk}}\frac{s_{aj}^2+s_{ak}^2}{s_{AK}^2}. \end{aligned}$$

In Appendix A.2 we show that the antenna functions correctly reproduce the DGLAP splitting kernels in the collinear limit.

**Evolution variables** We evolve gluon emission in the transverse momentum of the emission, defined as

28 $$\begin{aligned} t_\text {IF}^\text {emit} = p_{\perp \,\text {IF}}^2=\frac{s_{aj}s_{jk}}{s_{AK}+s_{jk}} =\frac{s_{aj}s_{jk}}{s_{aj}+s_{ak}}, \end{aligned}$$

with the phase-space limit $p_{\perp \,\text {IF}}^2\le s_{AK}(1-x_A)/x_A$. Figure 2b shows constant contours of $p_{\perp \,\text {IF}}^2$, as a function of the two branching invariants $s_{aj}$ and $s_{jk}$. Note that the phase space is limited by $s_{jk}\le s_{AK}(1-x_A)/x_A$ and $s_{aj}\le s_{AK}+s_{jk}$.

For branchings with flavour changes in the initial or final state we use the corresponding invariant, $s_{aj}$ or $s_{jk}$, respectively,

29 $$\begin{aligned} t_\text {IF}^\text {conv} = Q^2_\text {IF} = \left\{ \begin{array}{cl} s_{aj}&{} \text{ for }\,a\, \text{ converting } \text{ to/from } \text{ a } \text{ gluon }\\ s_{jk}&{} \text{ for }\,K \rightarrow q\bar{q} \end{array} ,\right. \end{aligned}$$

with the phase-space limits $s_{aj}\le s_{AK}/x_A$ and $s_{jk}\le s_{AK}(1-x_A)/x_A$.

**No-emission probability** With the definitions given above (5) for initial–final configurations reads

30 $$\begin{aligned} \Pi _n(t_{n},t_{n+1})= & {} \exp \left( - \int _{{s_{aj}}_{n+1}}^{{s_{aj}}_{n}}\!\!\!\!\!\!\!\!\!\!\!\!\text {d}s_{aj}\, \int _{{s_{jk}}_{n+1}}^{{s_{jk}}_{n}}\!\!\!\!\!\!\!\!\!\!\!\!\text {d}s_{jk}\, \int _0^{2\pi }\!\frac{\text {d}\phi }{2\pi }\, \right. \nonumber \\&\times \, \frac{\alpha _s(t)}{4\pi }\,\frac{s_{AK}}{(s_{AK}+s_{jk})^2}\, \mathcal {C}\, {\bar{a}}(s_{aj},s_{jk},s_{AK})\,\nonumber \\&\times \,\left. \frac{f_a(x_a,t)}{f_A(x_A,t)} \right) , \end{aligned}$$

with $t=t_\text {IF}(s_{aj},s_{jk},s_{ak})$. The subscript of the $s_{aj}$ and $s_{jk}$ integration limits indicates the association with the branching scales $t_n$ and $t_{n+1}$, respectively.

### Final–final configurations

We denote the pre- and post-branching partons participating in a final–final branching by $IK\rightarrow ijk$, with no recoils outside the antenna. In the following, we specify the phase-space factorisation, antenna functions, evolution variables and the resulting no-emission probability. More extensive descriptions of Vincia ’s final-state antenna-shower formalism can be found in [23, 39].

**Phase space** The phase-space factorisation reads

31 $$\begin{aligned} \text {d}\Phi _3(P\rightarrow p_i,p_j,p_k) = \text {d}\Phi _2(P\rightarrow p_I,p_K)\,\text {d}\Phi _\text {ant}^\text {FF} \end{aligned}$$

with the antenna phase space

32 $$\begin{aligned} \text {d}\Phi _\text {ant}^\text {FF} = \frac{1}{16\pi ^2}\,\frac{1}{s_{IK}^2}\, \text {d}s_{ij}\,\text {d}s_{jk}\,\frac{\text {d}\phi }{2\pi }. \end{aligned}$$

See Appendix A.1 for the explicit construction of the post-branching momenta.

**Antenna functions** The default final–final antenna functions are chosen to be the ones of [52] averaged over helicities. For gluon-emission antennae, these are

33 $$\begin{aligned} {\bar{a}}_{q\bar{q}\,g}^\text {FF}&={\bar{a}}(i_q,k_{\bar{q}},j_g) = \frac{1}{s_{IK}}\left( 2\,\frac{s_{ik}s_{IK}}{s_{ij}s_{jk}} + \frac{s_{jk}}{s_{ij}}+ \frac{s_{ij}}{s_{jk}}+1\right) ,\end{aligned}$$

34 $$\begin{aligned} {\bar{a}}_{gg\,g}^\text {FF}&=\bar{a}(i_g,k_g,j_g)\nonumber \\&= \frac{1}{s_{IK}}\left( 2\,\frac{s_{ik}s_{IK}}{s_{aj}s_{jb}} + \frac{s_{jk}}{s_{ij}}+ \frac{s_{ij}}{s_{jk}}- \frac{s_{jk}^2}{s_{ij}s_{IK}}\right. \nonumber \\&\quad \left. - \frac{s_{ij}^2}{s_{jk}s_{IK}} + \frac{3}{2} + \frac{s_{ij}+s_{jk}}{2\,s_{IK}} \right) ,\end{aligned}$$

35 $$\begin{aligned} \bar{a}_{qg\,g}^\text {FF}&={\bar{a}}(i_q,k_g,j_g) = \frac{1}{s_{IK}}\left( 2\,\frac{s_{ik}s_{IK}}{s_{aj}s_{jb}} + \frac{s_{jk}}{s_{ij}}+ \frac{s_{ij}}{s_{jk}}\right. \nonumber \\&\quad \left. - \frac{s_{ij}^2}{s_{jk}s_{IK}} + \frac{3}{2}\right) . \end{aligned}$$

For a final-state gluon splitting, the default is

36 $$\begin{aligned} {\bar{a}}_{xq\,\bar{q}}^\text {FF}={\bar{a}}(i_x,k_q,j_{\bar{q}})= & {} \frac{1}{2s_{jk}}\frac{s_{ij}^2+s_{ik}^2}{s_{IK}^2}+\frac{1}{2}\frac{s_{jk}}{s_{IK}^2}+\frac{s_{ik}}{s_{IK}^2}.\nonumber \\ \end{aligned}$$

In Appendix A.2 we show that the antenna functions correctly reproduce the DGLAP splitting kernels in the collinear limit.

**Evolution variables** We evolve gluon emission either in transverse momentum, which is the default choice, or in the antenna mass,

37 $$\begin{aligned} t_\text {FF}^\text {emit} = \left\{ \begin{array}{l} p_{\perp \,\text {FF}}^2=4\,\dfrac{s_{ij}s_{jk}}{s_{IK}}\\ m_{\text {A}\,\text {FF}}^2=2\,\text {min}(s_{ij},s_{jk}) \end{array}\right. . \end{aligned}$$

The upper phase-space limit is the parent antenna mass, $t_\text {FF}^\text {emit}\le s_{IK}$. Gluon splittings are evolved in the invariant mass of the quark-antiquark pair,

38 $$\begin{aligned} t_\text {FF}^\text {conv} = Q^2_\text {FF} = \left\{ \begin{array}{cl} s_{ij}&{} \text{ for }\quad i \quad \text{ being } \text{ the } \text{ gluon }\\ s_{jk}&{} \text{ for }\quad k \quad \text{ being } \text{ the } \text{ gluon } \end{array} \right. , \end{aligned}$$

with the same phase-space limit as before.

**No-emission probability** With the definitions given above (5) for final–final configurations reads

39 $$\begin{aligned} \Pi _n(t_{n},t_{n+1})~&=~\Delta _n(t_{n},t_{n+1}) \end{aligned}$$

40 $$\begin{aligned}&=~\exp \left( - \int _{{s_{ij}}_{n+1}}^{{s_{ij}}_{n}}\!\!\!\!\!\text {d}s_{ij}\int _{{s_{jk}}_{n+1}}^{{s_{ij}}_{n}}\!\!\!\!\!\text {d}s_{jk}\right. \nonumber \\&\quad ~\times \,\left. \int _0^{2\pi }\!\frac{\text {d}\phi }{2\pi }\, \frac{\alpha _s(t)}{4\pi }\,\frac{1}{s_{IK}^2}\,\mathcal {C}\, \bar{a}(s_{ij},s_{jk},s_{IK})\right) , \end{aligned}$$

with $t=t_\text {FF}(s_{ij},s_{jk},s_{IK})$. The subscript of the $s_{ij}$ and $s_{jk}$ integration limits indicates the association with the branching scales $t_n$ and $t_{n+1}$, respectively.

### The shower generator

We now illustrate how the shower algorithm generates branchings, starting from trial branchings generated according to a simplified version of the no-emission probability in Eq. (5). For definiteness we consider the specific example of initial–initial antennae, initial–final ones being handled in much the same way, with a PDF ratio that only involves one of the beams, and final–final ones not involving any PDF ratios at all. The full antenna-shower evolution (II+IF+FF) is combined with Pythia’s $p_\perp$-ordered multiple-parton-interactions (MPI) model, in a common interleaved sequence of evolution steps [8].

With the explicit form of the antenna phase space the no-emission probability reads

41 $$\begin{aligned} \Pi _n(t_\text {start},t_{n+1})&= \exp \left( -\int _{t_{n+1}}^{t_\text {start}}\! \text {d}s_{aj}\,\text {d}s_{jb}\,\frac{\alpha _s(t)\,\mathcal {C}}{4\pi }\,\right. \nonumber \\&\quad \,\left. \times \frac{s_{AB}}{s_{ab}^2}\, {\bar{a}}(s_{aj},s_{jb},s_{AB})\,R_\text {pdf} \right) \nonumber \\&= \exp \left( -\int _{t_{n+1}}^{t_\text {start}}\!\text {d}s_{aj}\text {d}s_{jb}a(s_{aj},s_{jb},s_{AB})\,R_\text {pdf} \right) , \end{aligned}$$

where the integral is written in terms of the invariants $s_{aj}$ and $s_{jb}$ and we have suppressed the trivial integration over $\phi$. In the second line, colour and coupling factors, as well as leftover factors coming from the antenna phase space are absorbed into a redefined antenna function, $a(s_{aj},s_{jb},s_{AB})$. To impose the evolution measure, we first change the integration variables from $s_{aj}$ and $s_{jb}$ to *t* and $\zeta$, where *t* has dimension $\mathrm {GeV} ^2$ and $\zeta$ is dimensionless. The definition of $\zeta$ is in somewhat arbitrary, as long as it is linearly independent of *t* and there exists a one-to-one map back and forth between $(s_{aj},s_{jb})$ and $(t,\zeta )$. Generally, the freedom to choose $\zeta$ can be utilised to make the $(t,\zeta )$ integrands and phase-space boundaries as simple and efficient as possible. Transformed to arbitrary $(t,\zeta )$, Eq. (41) now reads

42 $$\begin{aligned}&\Pi _n(t_\text {start},t_{n+1})\nonumber \\&\quad = \exp \left( -\int _{t_{n+1}}^{t_\text {start}}\!\text {d}t\,\text {d}\zeta \, |J|\,a(s_{aj},s_{jb},s_{AB})\,R_\text {pdf} \right) , \end{aligned}$$

with the Jacobian |*J*| associated with the transformation from $(s_{aj},s_{jb})$ to $(t,\zeta )$. Rather than solving the exact expression, we make three simplifications, the effects of which we will later cancel by use of the veto algorithm:

- Instead of the physical antenna functions, *a*, we use simpler (trial) overestimates, $\hat{a}(s_{aj},s_{jb},s_{AB})$. For instance the trial antenna function for gluon emission off an initial-state quark–antiquark pair is chosen to be
  43 $$\begin{aligned} \hat{a}_{q\bar{q}\,g}^\text {II}=2\,\frac{s_{ab}^2}{s_{AB}s_{aj}s_{jb}}. \end{aligned}$$
- Instead of the PDF ratio, $R_\text {pdf}$, we use the overestimate
  44 $$\begin{aligned} \hat{R}_\text {pdf} = \left( \frac{x_A}{x_a}\frac{x_B}{x_b}\right) ^\alpha \frac{f_a(x_A,t_\text {min})}{f_A(x_A,t_\text {min})} \frac{f_b(x_B,t_\text {min})}{f_B(x_B,t_\text {min})}, \end{aligned}$$
  where $t_\text {min}$ is the lower limit of the range of evolution variable under consideration and $\alpha$ a parameter, whose value is, wherever possible, chosen differently, depending on the type of branching, to give a good performance.
- In cases where the physical $\zeta$ boundaries depend on the evolution variable *t*, we allow trial branchings to be generated in a larger hull encompassing the physical phase space, with $\zeta$ boundaries that only depend on the *t* integration limits.

Having the trial no-emission probability, $\hat{\Pi }_n(t_\text {start},t_{n+1})$, at hand we solve

45 $$\begin{aligned} \hat{\Pi }_n(t_\text {start},t_{n+1}) = \mathcal {R}\quad \text {with}\quad \mathcal {R}\in [0,1] \end{aligned}$$

for $t_{n+1}$ to obtain the scale of the next branching. Due to the simplifications discussed above, this can be done analytically. We then generate another uniformly distributed random number, $\mathcal {R}_{\zeta }$, from which we obtain a trial $\zeta$ value by solving (again analytically),

46 $$\begin{aligned} \mathcal {R}_\zeta = \frac{\hat{I}_\zeta (\hat{\zeta }_\text {min},\zeta )}{\hat{I}_\zeta (\hat{\zeta }_\text {min},\hat{\zeta _\text {max}})} \end{aligned}$$

where $\hat{I}$ is the integral over all $\zeta$ dependence in $\hat{\Pi }_n(t_\text {start},t_{n+1})$.

Finally, a uniformly distributed trial $\phi = 2\pi \mathcal {R}_\phi$ can be generated, furnishing the last branching variable. We now make use of the veto algorithm to recover the exact integral in Eq. (42), as shown in [62]. First, any trial branching outside the physical phase space is rejected. Each physical trial branching is then accepted with the probability

47 $$\begin{aligned} \mathcal {O}(\hat{t},t_{n+1})\,P^\text {shower}=\mathcal {O}(\hat{t},t_{n+1})\,\frac{a(s_{aj},s_{jb},s_{AB})}{\hat{a}(s_{aj},s_{jb},s_{AB})}\,\frac{R_\text {pdf}}{\hat{R}_\text {pdf}}, \end{aligned}$$

where $\mathcal {O}(\hat{t},t_{n+1})$ represents the ordering condition with respect to some scale $\hat{t}$. In traditional, strongly ordered showers this scale is equal to the scale of the last branching $t_{n}$ and the ordering condition therefore is

48 $$\begin{aligned} \mathcal {O}(\hat{t},t_{n+1}) = \mathcal {O}(t_{n},t_{n+1}) = \Theta (t_{n}-t_{n+1}). \end{aligned}$$

For more details on the algorithm see Appendix A.3 and the Vincia  compendium distributed alongside with the code.

### Colour coherence and colour indices 

When assigning colour indices to represent colour flow after a branching, we adopt a set of conventions that are designed to approximately capture correlations between partons that are not LC-connected, based on the arguments presented in [49]. Specifically, we let the last digit of the ”Les Houches (LH) colour tag” [63, 64] run between 1 and 9, and refer to this digit as the ”colour index”. LC-connected partons have matching LH colour tags and therefore also matching colour/anticolour indices, while colours that are in a relative octet state are assigned non-identical colour/anticolour indices. Hence the last digit of a gluon colour tag will never have the same value as that of its anticolour tag. This does not change the LC structure of the cascade; if using only the LH tags themselves to decide between which partons string pieces should be formed, the extra information is effectively just ignored. It does, however, open for the possibility of allowing strings to form between non-LC-connected partons that ”accidentally” end up with matching indices, in a way that at least statistically gives a more faithful representation of the full SU(3) group weights than the strict-LC one [49].

The new aspect we introduce here is to assign colour indices after each branching, whereas the model in [49] operated at the purely non-perturbative stage just before hadronisation. Furthermore, for gluon emissions, we choose to let the colour tag of the parent antenna be inherited by the daughter antenna with the largest invariant mass, while the one with the smaller invariant mass is assigned a new colour tag (subject to the rules described above). This is intended to preserve the coherence structure as seen by the rest of the event, so that, for instance, the new colour created in a near-collinear branching is attributed to the new small antenna, while the colour tag of the parent antenna continues on as the tag of the larger of the daughters. An advantage of this approach is that the octet nature of intermediate gluons, e.g. in collinear $g\rightarrow gg$ branchings, is preserved by our treatment, which is not the case in the implementation of [49].

**Fig. 5:**
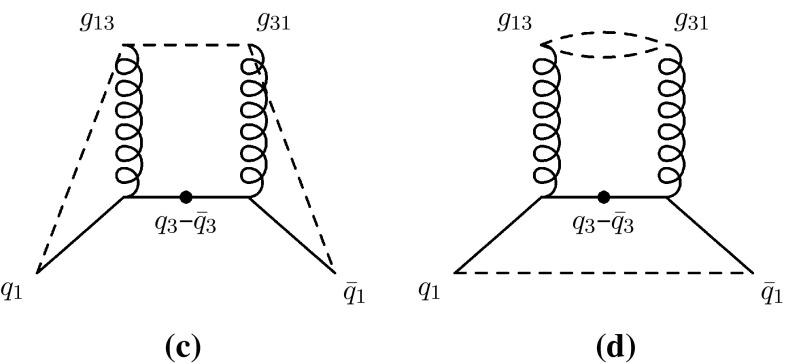
With a probability suppressed by $1/N_C^2$, the same *colour* index may occur twice in the diagram shown in Fig. 4b, illustrated here in the *left-hand pane*. When this occurs, the string topology shown in the *right-hand pane* is also possible (The model of [49] invokes a string-length minimisation argument to decide which is realised)

In Figs. 4 and 5 we illustrate our approach, and the ambiguity it addresses. For definiteness, and for simplicity, we consider the specific case of $Z\rightarrow q gg\bar{q}$, but the arguments are general. The two diagrams in Fig. 4 show the outgoing partons, produced by a *Z* boson decaying at the point denoted by $\bullet$. Both axes correspond to spatial dimensions, hence time is indicated roughly by the radial distance from the *Z* decay point. Examples of the colour indices defined above are indicated by subscripts, hence e.g., $g_{13}$ denotes a gluon carrying anticolour index 1 and colour index 3. Due to our selection rule, the type of assignment represented by Fig. 4a is always selected when $m_{gg}$ is small, $s_{gg}< s_{qg}$, while the one represented by Fig. 4b is selected when $s_{qg}<s_{gg}$ (when the second emission occurs in the $q-g$ antenna, and completely analogously when it occurs in the $g-\bar{q}$ one). The subleading-colour ambiguity illustrated by Fig. 5 can only occur for the latter type of assignment, hence will be absent in our treatment for collinear $g\rightarrow gg$ branchings (where the flow represented by Fig. 4a dominates), in agreement with the collinearly branching gluon having to be an octet. We regard this as an improvement on the treatment in [49], in which there was no mechanism to prevent collinear gluons from ending up in an overall singlet state; see also the remarks accompanying [49, Fig. 15].

As a last point, we remark that this new assignment of colour tags is currently left without impact, but is implemented in order to enable future studies, such as colour reconnection within Vincia .

### Uncertainty estimations 

Traditionally, shower uncertainties are evaluated by systematic up/down variations of each model parameter, which mandates the generation of multiple event sets, one for each variation. To avoid this time-consuming procedure, Vincia  instead generates a vector of variation weights for each event [39], where each of the weights corresponds to varying a different parameter. A separate publication details the formal proof of the validity of the method [65], which we have here extended to cover both the initial- and final-state showers in Vincia . (**Note added in proof:** during the publication of this manuscript, two further papers appeared reporting similar implementations in Herwig and Sherpa, see [66, 67].) In this section, we only give a brief overview of the implementation, referring to [39, 65] for details and illustrations. Technical specifications for how to switch the uncertainty bands on and off in the code, and how to access them, are provided in Vincia ’s HTML User Reference [46].

During the shower step, in which a trial branching gets accepted with the probability $P_\text {def}$ given in Eq. (47), the probability of the same branching to occur with a variation in e.g. the choice of renormalisation scale or antenna function is calculated,

49 $$\begin{aligned} P_\text {var} = \frac{\text {VAR}}{\text {DEF}}\,\,P_\text {def} \end{aligned}$$

where $\text {DEF}$ and $\text {VAR}$ are symbols representing the default and variation choice, respectively. In the case of an accepted branching the variation weight of the event gets simply multiplied with $P_\text {var}/P_\text {def}$, and for rejected branchings with

50 $$\begin{aligned} \frac{1-P_\text {var}}{1-P_\text {def}} \end{aligned}$$

to correctly take the no-emission probability into account.

The variations currently implemented in Vincia  are the following:

- Vincia ’s default settings, with default antenna functions, scale choices and colour factors.
- Variation of the renormalisation scale. Using $\alpha _s(t/k_\mu )$ and $\alpha _s(t\,k_\mu )$, with a user-specifiable value of the additional scaling factor $k_\mu$.
- Variation of the antenna functions. Using antenna sets with large and small nonsingular terms, representing unknown (but finite) process-dependent LO matrix-element terms. Note that these are cancelled by LO MECs (up to the matched orders).
- $\alpha _s$-suppressed counterparts of the finite-term variations above^8^ which are not cancelled by (LO) MECs.
- Variation of the colour factors. All gluon emissions use the colour factor of either $C_A=3$ or $2C_F=8/3$.
- Modified $$P_\text {imp}$$ factor,
  51 $$\begin{aligned} P_\text {imp}' = \frac{\hat{t}^2}{\hat{t}^2+t^2}. \end{aligned}$$

Note that, except for the first one, the variations are taken with respect to the user-defined settings. All of these variations are applied in the shower and the MECs, and they are limited to branchings in the hard system, i.e. they are for instance not applied in the showering of multi-parton interactions.

### Limitations

For completeness, we note that a few options and extensions of the existing Vincia  final-state shower have not yet been implemented in Vincia  2.0. These will remain available in earlier versions of the code (limited to pure final-state radiation hence mostly of interest for $e^+e^-$ studies) and may reappear in future versions, subject to interest and available manpower. Briefly summarised, this concerns the following features:

- Sector showers [28]: a variant of the antenna-shower formalism in which a single term is responsible for generating all contributions to each phase-space point. It has some interesting and unique properties including being one-to-one invertible and producing fewer (one) term at each order of GKS matrix-element corrections leading to the numerically fastest matching algorithm we are aware of (see [28]), at the price of requiring more complicated antenna functions with more complicated phase-space boundaries. For the initial-state extension of Vincia   we have so far focussed on the technically simpler case of “global” (as opposed to sector) antennae.
- One-loop matrix-element corrections. The specific case of one-loop corrections for hadronic *Z* decays up to and including 3 jets was studied in detail by HLS [40]. The extension of this method to hadronic initial states, and a more systematic approach to one-loop corrections in Vincia  in general, will be a major goal of future efforts.
- Helicity dependence [52]. The shower and matrix-element-correction algorithms described in this paper pertain to unpolarised partons. Although this is fully consistent with the unpolarised nature of the initial-state partons obtained from conventional parton distribution functions (PDFs), we note that an extension to a helicity-dependent formalism could nonetheless be a relatively simple future development. Moreover, we expect this would provide useful speed gains for the GKS matrix-element correction algorithm equivalent to those observed for the final-state algorithm [52].
- Full-fledged fermion mass effects [68]. Our treatment of mass effects for initial-state partons is so far limited to one parallelling the simplest treatment in conventional PDFs, the “zero-mass-variable-flavour-number (ZMVF) scheme”. In this scheme, heavy-quark PDFs are set to zero below the corresponding mass threshold(s) and are radiatively generated above them by $g\rightarrow Q\bar{Q}$ splittings, with $m_Q$ formally set to zero in those splittings and for the subsequent heavy-quark evolution. Thus, in Vincia  2.0, all partons are assigned massless kinematics, but $g\rightarrow Q\bar{Q}$ splittings are switched off (also in the final state) below the physical mass thresholds. This only gives a very rough approximation of mass effects [69, 70] but at least avoids generating unphysical singularities. Beyond the strict ZMVF scheme, optionally and for final-state branchings only, we allow for a set of universal antenna mass corrections to be applied and/or for tighter phase-space constraints to be imposed, with the latter obtained from the would-be massive phase-space boundaries. We note that a mixed treatment similar to the one currently employed by Pythia, with massive/massless kinematics for outgoing/incoming partons, respectively, would not be straightforward to adopt in Vincia   as it would be inconsistent with the application of on-shell matrix-element corrections.
- The so-called “Ariadne factor” [21] for gluon splitting antennae
  52 $$\begin{aligned} P_\text {Ari} = \frac{2s_N}{s_N+s_P}, \end{aligned}$$
  with $S_N$ the invariant mass squared of the colour neighbour on the other side of the splitting gluon and $s_P$ the invariant mass squared of the parent (splitting) antenna is limited to its original purpose, that of improving the description of 4-jet observables in *Z* decay, and is not applied outside that context.

## Matrix-element corrections

In this section we focus on the MEC formalism in Vincia  and discuss our strategy for reaching the non-ordered parts of phase space, both with respect to the factorisation scale in the case of the first branching and with respect to previous branching scales.

Note that in this paper all matrix elements are generated with MadGraph [50, 71]. The output is suitably modified to extract the leading-colour matrix element, i.e. to not sum over colour permutations, but pick the (diagonal) entry in MadGraph’s colour matrix that corresponds to the colour order of interest. All plots shown in this paper are based on leading-colour matrix elements.

### Hard jets in non-QCD processes

In this section we describe our formalism to combine events which are accompanied by at least one very hard jet, with the ones which are not. We emphasise that the considerations are general and apply to any processes that do not exhibit QCD jets at the Born level.

We first consider the Born inclusive cross section, differential in the Born phase space,

53 $$\begin{aligned} \text {d}\sigma _B^\text {incl}(t_\text {fac}) = f_0(x_0,t_\text {fac})\,|\mathcal {M}_B|^2\,\text {d}\Phi _B, \end{aligned}$$

where $t_\text {fac}$ is the factorisation scale, subscript zero emphasises that flavour and energy fraction correspond to the state $\Phi _B$ (subscript one will then correspond to the state $\Phi _{B+1}$ and so on), and the second PDF factor has been dropped for the sake of readability.

Since the ISR shower formally corresponds to a “backwards” evolution of the PDFs [33], the factorisation scale represents the natural upper bound (starting scale) for the initial-state shower evolution. This implies that any phase-space points with $t > t_\text {fac}$ will not be populated by the shower, potentially leaving a “dead zone” for high-*t* emissions. In principle, the freedom in choosing the evolution variable can be exploited to define *t* in such a way that the entire physical phase space becomes associated with scales $t<t_\text {fac}$ [30], including points with physical $p_\perp ^2\gg t_\text {fac}$. Here, however, we wish to maintain a close correspondence between the evolution variable and the physical (kinematic) $p_\perp$, requiring the development of a different strategy.

The approach used internally in Pythia is that of “power showers” (with [72] or without [73] matrix-element corrections): starting the shower from a scale $t_\text {start}$ that is higher than the factorisation scale. This method has been criticised for producing too hard jet emission spectra and violating the factorisation ansatz. Though the improved power showers defined in [74, 75] are better behaved (dampening the LL $1/p_\perp ^2$ kernels to explicitly subleading $Q^2/p_\perp ^4$ ones for emissions above the *Q* scale of the basic process), shortcomings are still present. Consider, for example, the Born exclusive cross section at an arbitrary shower cutoff, differential in the Born phase space, scale $t_\text {cut}$,

54 $$\begin{aligned} \text {d}\sigma _B^\text {excl}(t_\text {cut})&= \Pi _0(t_\text {start},t_\text {cut})\,f_0(x_0,t_\text {fac})\, |\mathcal {M}_B|^2\,\text {d}\Phi _B \end{aligned}$$

55 $$\begin{aligned}&= \frac{f_0(x_0,t_\text {fac})}{f_0(x_0,t_\text {start})}\,f_0(x_0,t_\text {cut})\, \Delta _0(t_\text {start},t_\text {cut})\,|\mathcal {M}_B|^2\,\text {d}\Phi _B. \end{aligned}$$

Unless $t_\text {start}=t_\text {fac}$, there appears an undesired PDF ratio, which reflects the difference in the factorisation and shower starting scale. To avoid this problem, we introduce two separate event samples, both initiated by the same matrix element with the same factorisation scale, as in Eq. (53). They are generated simultaneously, producing a single stream of ordinary randomly mixed, weighted events, with no need for external recipes to combine them. The first sample creates events that do not have a hard jet, by starting the shower at the factorisation scale (hence leaving the region $t>t_\text {fac}$ unpopulated). The second event sample is responsible for all events with at least one jet with scale $t>t_\text {fac}$. This sample is initialised by first reweighting the Born-level events such that the (temporary) factorisation scale is set to the phase-space maximum, $t_\text {max}$, and the shower algorithm is started from that scale. Events that do not produce at least one branching before the original (Born-level) factorisation scale is reached are vetoed, resulting in a total contribution to the inclusive cross section in Eq. (53) of

56 $$\begin{aligned} \frac{f_0(x_0,t_\text {max})}{f_0(x_0,t_\text {fac})}\,f_0(x_0,t_\text {fac})\, \left( 1-\Pi _0(t_\text {max},t_\text {fac})\right) \,|\mathcal {M}_B|^2\,\text {d}\Phi _B. \end{aligned}$$

Adding the two event samples together yields the new inclusive cross section,

57 $$\begin{aligned} \text {d}\sigma _B^\text {incl}(t_\text {fac}) =&~ f_0(x_0,t_\text {fac})\,|\mathcal {M}_B|^2\,\text {d}\Phi _B +\, f_0(x_0,t_\text {start})\, (1\nonumber \\&-\Pi _0(t_\text {start},t_\text {fac}))\, |\mathcal {M}_B|^2\,\text {d}\Phi _B \nonumber \\ =&~ f_0(x_0,t_\text {fac})\,|\mathcal {M}_B|^2\,\text {d}\Phi _B ~\nonumber \\&+\int \limits _{t_\text {fac}}^{t_\text {start}}\!\!\text {d}t\, f_1(x_1,t)\,\mathcal {A}(t)\, \Delta _0(t_\text {start},t_\text {})\,|\mathcal {M}_B|^2\,\text {d}\Phi _B , \end{aligned}$$

where $\mathcal {A}(t)$ contains all antenna functions, coupling and colour factors. By virtue of adding and subtracting $f_0(x_0,t_\text {fac})\,|\mathcal {M}_B|^2\,\text {d}\Phi _B \Delta _0(t_\text {start},t_\text {fac})$ and using the DGLAP equation

58 $$\begin{aligned} f_0(x_0,t_\text {start})&= f_0(x_0,t_\text {fac})\,\Delta _0(t_\text {start},t_\text {fac})\nonumber \\&+\,\int \limits _{t_\text {fac}}^{t_\text {start}}\!\!\text {d}t\,f_1(x_1,t)\,\mathcal {A}(t)\, \Delta _0(t_\text {start},t_\text {}) \end{aligned}$$

this becomes

59 $$\begin{aligned} \text {d}\sigma _B^\text {incl}(t_\text {fac}) =&~ f_0(x_0,t_\text {start})\,|\mathcal {M}_B|^2\,\text {d}\Phi _B \nonumber \\&+ f_0(x_0,t_\text {fac})\, (1{-}\Delta _0(t_\text {start},t_\text {fac})) |\mathcal {M}_B|^2\,\text {d}\Phi _B. \end{aligned}$$

Expanding (57) to $\mathcal {O}(\alpha _s)$ yields

60 $$\begin{aligned} \text {d}\sigma _B^\text {incl}(t_\text {fac}) =&~ f_0(x_0,t_\text {fac})\,|\mathcal {M}_B|^2\,\text {d}\Phi _B ~\nonumber \\&+\, \int _{t_\text {fac}}^{t_\text {start}}\!\!\text {d}t\,f_1(x_1,t)\,\mathcal {A}(t)\, |\mathcal {M}_B|^2\,\text {d}\Phi _B. \end{aligned}$$

Expanding (59) instead yields

61 $$\begin{aligned} \text {d}\sigma _B^\text {incl}(t_\text {fac}) =&~ f_0(x_0,t_\text {start})\,|\mathcal {M}_B|^2\,\text {d}\Phi _B ~\nonumber \\&+\, f_0(x_0,t_\text {fac}) \,\int _{t_\text {fac}}^{t_\text {start}}\!\!\text {d}t\,\mathcal {A}(t)\, |\mathcal {M}_B|^2\,\text {d}\Phi _B, \end{aligned}$$

which is seemingly at odds with (60). The problem is that both (60) and (61) have been derived by expanding, so that their relation through the DGLAP equation is lost. The crucial point—which is obscured after expanding—is already contained in (57): the inclusive cross section is calculated with a sensible factorisation scale $t_\text {fac}$, while all branchings with scales $t>t_\text {fac}$ contribute, in a controlled way, at higher orders. Section 4 contains some illustrations of the effects of these corrections for physical observables such as the dilepton rapidity and $p_\perp$ spectra in Drell–Yan processes.

The inclusive cross section obtained from Eq. (57) does not reduce to the zero-parton Born cross section, the changes being only due to hard emissions which have not been incorporated in the first term in (57). This differs from cross section changes in CKKW-inspired merging prescriptions [76, 77], which arise from real–virtual mismatches at the merging scale,^9^ or from the definition of the inclusive cross section in unitarised merging schemes [78, 79]. In the latter, the inclusive cross section is almost entirely given by the first term in (57), and only changed by ”incomplete” states which cannot be associated with valid parton-shower histories. The definition of what is deemed an ”incomplete state” is not conventional and thus may depend on the details of a particular implementation. Note, however, that [78–80] do not advocate including the factors ”$\Delta _0$” when reweighting ”incomplete” states. This could lead to interesting differences in observables relying on very boosted *Z*-boson momenta.

We note that, although the described method of adding hard jets in non-QCD processes is the default choice in Vincia , we include the possibility to perform an ordinary shower, starting off the factorisation scale $t_\text {fac}$. This is the recommended option when combining Vincia ’s shower with external matching and merging schemes.

**Fig. 6:**
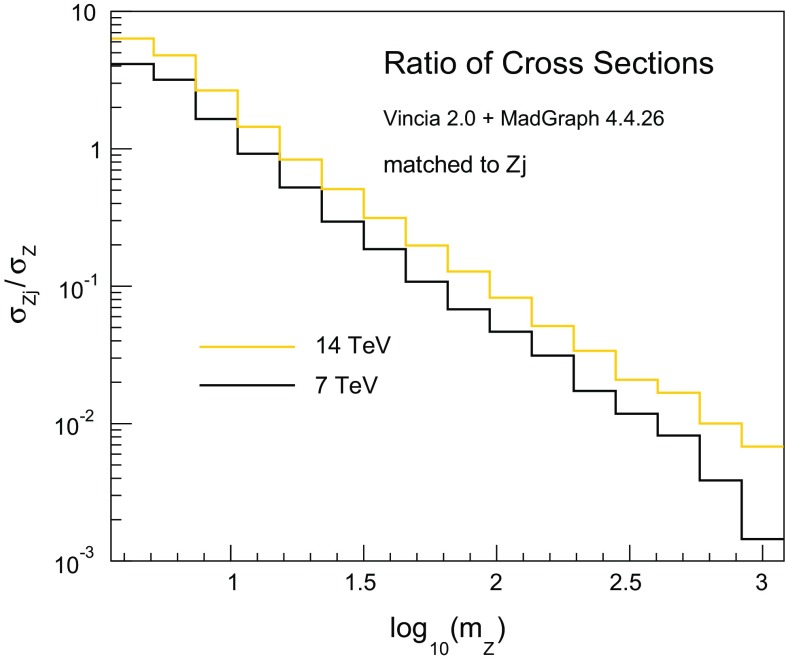
Ratio of cross sections in *Z* production as a function of the *Z* mass for $\sqrt{s}=7~\text {GeV}$ (*black*) and $\sqrt{s}=14~\text {GeV}$ (*orange*)

In Fig. 6 we show the relative contribution of the two event samples in *Z* production, as a function of the *Z* mass,

62 $$\begin{aligned} \frac{\sigma _{Zj}}{\sigma _{Z}}\,\,(m_Z) = \frac{f_0(x_0,s)\,\left( 1-\Pi _0(s,m_Z^2)\right) \,\left| \mathcal {M}_{Zj}\right| ^2\,\Phi _{Zj}}{f_0(x_0,m_Z^2)\,\left| \mathcal {M}_Z\right| ^2\,\Phi _Z}, \end{aligned}$$

with $\sqrt{s}=7~\text {GeV}$ (black) and $\sqrt{s}=14~\text {GeV}$ (orange). As expected, the contribution of events with at least one hard jets is larger for decreasing *Z* masses and increasing centre-of-mass energies. For both values of $\sqrt{s}$ the Born event sample eventually dominates for *Z* masses above $\mathcal {O}\,(10~\text {GeV})$.

### Strong ordering compared with tree-level matrix elements

To validate the quality of the antenna shower, we use large samples of $pp\rightarrow Zjj$ phase-space points, generated with Rambo [81] (an implementation of which is included in Vincia ). We cluster all of the phase-space points back to the corresponding $pp\rightarrow Z$ phase-space point, using the exact inverse of the $2\rightarrow 3$ recoil prescription used in the shower as a clustering algorithm; see Appendix A for the kinematics map used here. This allows one to reconstruct all possible ways in which the shower could have populated a certain phase-space point, analogously to the study carried out for final-state radiation in [39] (see also [82]). Comparing the shower approximation with the LO matrix element for $q_1\bar{q}_2\rightarrow Zg_3g_4$ yields the tree-level PS-to-ME ratio

**Fig. 7:**
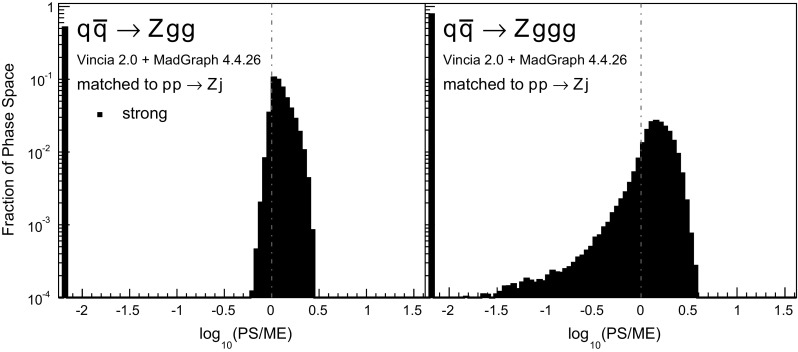
Antenna shower, compared to matrix elements: distribution of $\text {log}_{10}(\text {PS}/\text {ME})$ in a flat phase-space scan of the full phase space. Contents normalised to the number of generated points. Gluon emission only

63 $$\begin{aligned} R_4&\equiv \frac{\Theta (t_{\,\widehat{43}}-t_3)\,\mathcal {C}_{qg\,g}\, {\bar{a}}_{qg\,g}^\text {IF}(1,4,3)\, \mathcal {C}_{q\bar{q}\,g}\,{\bar{a}}_{q\bar{q}\,g}^\text {II}(\widehat{13},2,\widehat{43})\, \left| \mathcal {M}_Z(Z)\right| ^2}{\left| \mathcal {M}_{Zgg}(1,2;Z,3,4)\right| ^2} \nonumber \\&\quad +\, \frac{\Theta (t_{\,\widehat{34}}-t_4)\,\mathcal {C}_{\bar{q}g\,g}\, {\bar{a}}_{\bar{q}g\,g}^\text {IF}(2,3,4)\, \mathcal {C}_{q\bar{q}\,g}\,{\bar{a}}_{q\bar{q}\,g}^\text {II}(1,\widehat{24},\widehat{34})\, \left| \mathcal {M}_Z(Z)\right| ^2}{\left| \mathcal {M}_{Zgg}(1,2;Z,3,4)\right| ^2} \phantom {\frac{\dfrac{1}{2}}{1}} \end{aligned}$$

where the strong-ordering condition is incorporated by the $\Theta$ step functions and the hatted variables $\widehat{aj}$ denote clustered momenta. The two terms correspond to the two possible shower histories—obtained from starting by clustering either gluon 3 or 4, respectively—with the sequential clustering scales

64 $$\begin{aligned} t_3 = p_{\perp \,\text {IF}}^2(g_3)\quad \text {and}\quad t_{\,\widehat{43}} = p_{\perp \,\text {II}}^2(g_{\,\widehat{43}}), \end{aligned}$$

65 $$\begin{aligned} t_4 = p_{\perp \,\text {IF}}^2(g_4)\quad \text {and}\quad t_{\,\widehat{34}} = p_{\perp \,\text {II}}^2(g_{\,\widehat{34}}). \end{aligned}$$

$R_4$ therefore gives a measure of how much the shower under- or overcounts the tree-level matrix element. With the first emission already corrected^10^ Eq. (63) reduces to

66 $$\begin{aligned} R_4 =&~ \frac{\Theta (t_{\,\widehat{43}}-t_3)\,\mathcal {C}_{qg\,g}\, \bar{a}_{qg\,g}^\text {IF}(1,4,3)\, \left| \mathcal {M}_{Zg} (\widehat{13},2,\widehat{43}) \right| ^2}{\left| \mathcal {M}_{Zgg}(1,2;Z,3,4)\right| ^2} \nonumber \\&~+~ \frac{\Theta (t_{\,\widehat{34}}-t_4)\,\mathcal {C}_{\bar{q}g\,g}\, \bar{a}_{\bar{q}g\,g}^\text {IF}(2,3,4)\, \left| \mathcal {M}_{Zg} (1,\widehat{24},\widehat{34}) \right| ^2}{\left| \mathcal {M}_{Zgg}(1,2;Z,3,4)\right| ^2}. \phantom {\frac{\dfrac{1}{2}}{1}} \end{aligned}$$

Higher-order PS-to-ME ratios are constructed in a similar way.

Histograms showing the logarithmic distribution of the PS-to-ME ratios for $q\bar{q}\rightarrow Zgg$ and $q\bar{q}\rightarrow Zggg$, in a flat scan over the full phase space, comparing a strongly ordered shower with the LO amplitude squared, are shown in Fig. 7. The spike on the very left of the histograms corresponds to the part of phase space where there are no ordered shower histories. Note that about 35 % of the whole phase space in a flat scan of $q\bar{q}\rightarrow Zgg$ does not have an ordered shower path, a significantly higher fraction than the roughly 2 % found for the final-state phase spaces in [39]. We interpret this as due to the significantly larger size of the initial-state phase space, which is not limited by the original antenna invariant mass but only by the hadronic CM energy. The binning of the histogram is chosen such that the two bins around 0 (marked with a grey dashed line) correspond to the shower having less than 10 % deviation to the tree-level matrix element. For the shower with strong ordering about 10 % of the total number of phase-space points, corresponding to about 15 % of the phase space with at least one ordered path, populate these two bins.

**Fig. 8:**
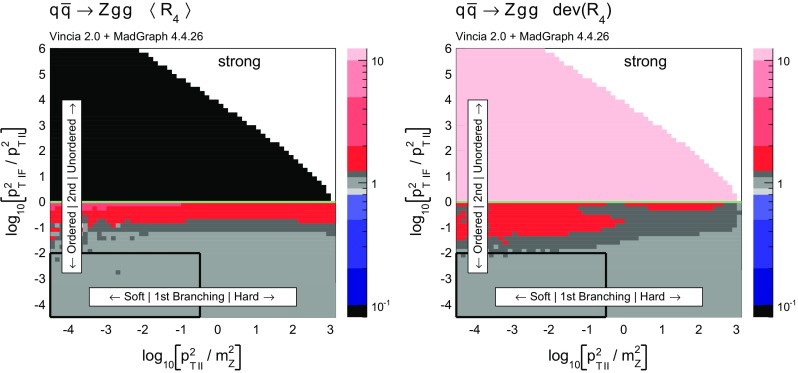
The value of $\langle R_4 \rangle$ (*left*) and $\text {dev}(R_4)$ (*right*), differentially over the 4-parton phase space, with $p_\perp ^2$ ratios characterizing the first and second emissions on the *x*- and *y* axis, respectively. Strong ordering in the shower, with gluon emission only

**Fig. 9:**
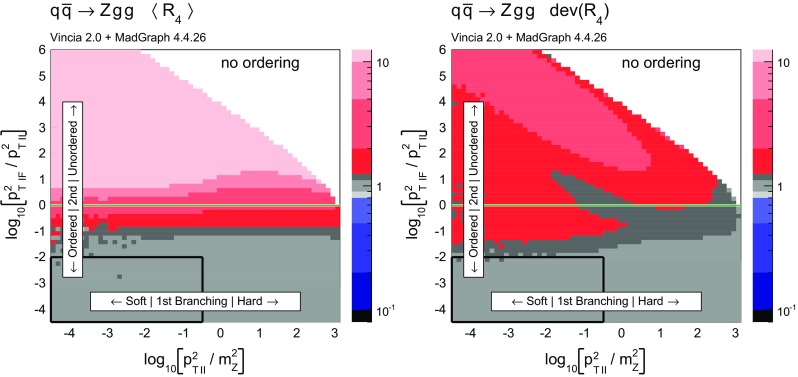
The value of $\langle R_4 \rangle$ (*left*) and $\text {dev}(R_4)$ (*right*), differentially over the 4-parton phase space, with $p_\perp ^2$ ratios characterizing the first and second emissions on the *x*- and *y* axis, respectively. No ordering in the shower, with gluon emission only

To gain an understanding of where in phase space significant deviations between the shower approximation and the LO amplitudes squared occur, we consider the 2D distributions presented in Figs. 8 and 9. For all plots, the *x* axes represent the degree of ordering of the first ($Z\rightarrow Zg$) emission, while the *y* axis represents the degree of ordering of the second ($Zg \rightarrow Zgg$) emission, defined more precisely below. Note that, since the phase spaces have more than 2 dimensions, each bin still represents an average of different phase-space points with the same *x* and *y* coordinates. Since the ratios on the axis are plotted logarithmically, zero denotes the border between ordered and unordered paths. The black-framed box in the lower left-hand corner of the plots highlights the strongly ordered region defined by $p_{\perp \,\text {IF}}^2 \ll p^2_{\perp \,\text {II}} \ll m^2_Z$, in which any (coherent) LL shower approximation is expected to give reasonable results. In the left-hand panes, grey colours signify less than 20 % deviation from a ratio unity (with the middle shade corresponding to less than 10 % deviation, corresponding to near-perfect agreement). Red shades signify increasingly large deviations, with contours at 2, 5, and 10. Blue contours extend to 1 / 2, 1 / 5, and 1 / 10, while black indicates regions where the shower answer is less than one tenth of the matrix-element answer. In the right-hand panes, the same colour scale is used to show a measure of the width of the $R_4$ distribution in each bin, defined below. These plots are intended to ensure that an average good agreement in the left-hand pane is not merely accidental, but also corresponds to a narrow distribution.

**Fig. 10:**
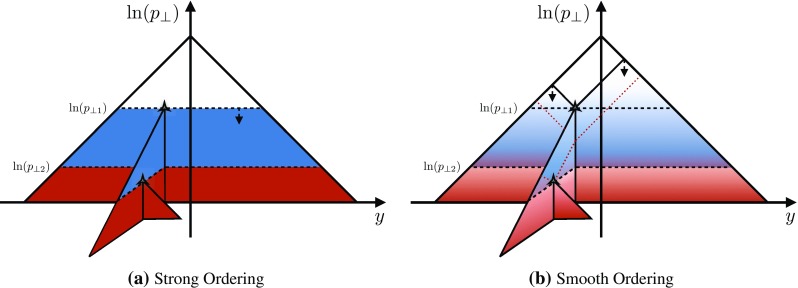
Illustration of the phase-space coverage of $p_\perp$-ordered dipole/antenna showers with **a** strong and **b** smooth ordering, in the “origami” plane of $\ln p_\perp$ vs. rapidity

In Fig. 8, the left-hand pane provides a clear illustration of the dead zone for the process $q\bar{q}\rightarrow Zgg$ in a strongly $p_\perp ^2$-ordered antenna shower. Each bin of the two-dimensional histogram shows the average of the value of $R_4$ in Eq. (66) over all phase-space points populating that bin. For every phase-space point there are two possible (not necessarily ordered) shower histories, with different scales for the first branching, $p_{\perp \,\text {II}}^2$, and second one, $p_{\perp \,\text {IF}}^2$. The combination of scales that correspond to the path with the smaller scale of the second branching is used to characterise the phase-space point. The black region in Fig. 8 for strong ordering corresponds to the spike in Fig. 7. Since there are two shower histories, there is in principle the possibility that the second history (which was not used to characterise the phase-space point) contributes as an ordered history, but this does not appear to happen anywhere in the region classified as unordered. The plot on the right shows the deviation within each bin, which we define to be

67 $$\begin{aligned} \text {dev}(R_4)=10^{\sqrt{\langle \text {log}_{10}^2(R_4)\rangle - \langle \text {log}_{10}(R_4)\rangle ^2}}, \end{aligned}$$

since the distribution of $R_4$ is naturally a logarithmic one. We assign a deviation of 10 to the dead zone, since the log would otherwise not be defined. As mentioned above, the deviation is intended to illustrate whether an average value in the left plot is achieved by a broad or a narrow distribution.

One could force the dead zone to disappear by simply removing the ordering condition and starting the shower at the phase-space maximum for each antenna. However, as can be seen from Fig. 9, this would highly overcount the matrix element in the unordered region, again parallelling the observations for the equivalent case of final-state radiation in [39]. The strong-ordering condition is clearly a better approximation to QCD, even if it does not fill all of phase space. To improve the shower, we will therefore need to allow the shower to access the whole phase space while suppressing the overcounting in the unordered region.

### Smooth ordering compared with tree-level matrix elements

As we saw in the previous section, a strongly ordered shower has a significant dead zone for hard emissions, especially in the initial-state sector. We now want to focus on how to remove them by generalising Vincia ’s “smooth ordering” [39] to initial-state phase spaces. Reference [39] shows that replacing the step function of an ordered shower with a smooth suppression factor leads to a surprisingly good description of the unordered region in *Z* decay. Based on this study, an improved version of the shower accept probability in Eq. (47), which allows one to take “unordered” branchings into account is

68 $$\begin{aligned} \mathcal {O}(\hat{t},t)\,P^\text {shower}=P_\text {imp}\,P^\text {shower}= \frac{\hat{t}}{\hat{t} + t}\,P^\text {shower}, \end{aligned}$$

where *t* is the scale of the trial branching at hand and $\hat{t}$ is the reference scale.

The difference between conventional strong ordering and Vincia ’s $P_\mathrm {imp}$-suppressed smooth ordering can be illustrated by considering so-called origami diagrams [83–85], in which the antenna (or, equivalently, dipole) phase space is depicted in terms of $\ln (p_\perp ^2)$ versus rapidity. Defining these by our gluon-emission evolution variable, $p_\perp ^2 = m^2_{12} m^2_{23}/m^2$ and by $y = \frac{1}{2} \ln (m^2_{12}/m^2_{23})$, respectively, for an antenna with total invariant mass *m* splitting into two smaller antennae with masses $m_{12}$ and $m_{23}$, the leading (double-logarithmic) contribution to the branching probability is transformed to just a constant over the antenna phase space,

69 $$\begin{aligned} \text {d}P \sim \frac{\mathcal {C} \alpha _s}{2\pi } \, {\mathrm {d}}\ln p_\perp ^2 \, {\mathrm {d}}y, \end{aligned}$$

where $\mathcal {C}$ is the colour factor normalised so that $\mathcal {C}\rightarrow N_C$ in the leading-colour limit. The phase-space boundary for gluon emissions with $p_\perp \ll m$ is determined by $y_{\mathrm {max}} (p_\perp ) = \frac{1}{2}\ln (m^2/p_\perp ^2)$, so that the rapidity range available for emissions at a given $p_\perp$ defines a triangular region,

70 $$\begin{aligned} \Delta y (p_\perp )= \ln (m^2/p_\perp ^2) = \ln (m^2) - \ln (p_\perp ^2), \end{aligned}$$

corresponding to the outer hulls of the diagrams shown in Fig. 10.

For an emission at any given value of $p_{\perp 1}^2 = m_{12}^2 m_{23}^2 / m^2$, the total rapidity range (at that $p_\perp$ value) is unchanged by the branching,

71 $$\begin{aligned} \Delta y(p_{\perp 1})= & {} \underbrace{\ln (m^2) - \ln (p_{\perp 1}^2)}_{\text{ pre-branching }} \nonumber \\= & {} \underbrace{ \ln (m_{12}^2) - \ln (p_{\perp 1}^2) + \ln (m_{23}^2) - \ln (p_{\perp 1}^2)}_{\text{ post-branching }}, \end{aligned}$$

cf. the dashed line at $\ln (p_\perp ) = \ln (p_{\perp 1})$ in the figure. For soft emissions, however, say at a reference value of $p_{\perp } = 1\,\mathrm {GeV}$, the post-branching configuration covers a total rapidity range which is larger by

72 $$\begin{aligned}&\Delta y (1\,\mathrm {GeV})_\mathrm {post} - \Delta y (1\,\mathrm {GeV})_\mathrm {pre} \nonumber \\&\quad = \ln (m_{12}^2) + \ln (m_{23}^2) - \ln (m^2)~=~\ln (p_\perp ^2). \end{aligned}$$

The additional phase space “opened up” by the branching can hence be represented by adding a double-sided isoceles right triangle to the origami diagram, with side lengths $\ln (p_{\perp 1})$, which—for lack of a better direction—is drawn pointing out of the original plane. Restricting the subsequent shower evolution to populate only the region below the $p_{\perp 1}$ scale produces a strongly ordered shower, illustrated in Fig. 10a with the blue and red shaded regions representing the phase space accessible to a second and third branching, respectively. The case of smooth ordering is illustrated in Fig. 10b for the same sequence of branchings. In this case, each of the antennae produced by the first branching are allowed to evolve over their full phase spaces, and their respective full phase-space triangles are therefore now included in the diagram, using solid black lines for the first branching and red dotted lines for the phase-space limits after the second branching. The suppression of the branching probability near and above the branching scale is illustrated by reducing the amount of shading of the corresponding regions. Comparing the figures, one can see that we expect no change in the total range or integrated rate of soft emissions (at the bottom of the diagrams). The only effects occur near and above the branching scale where the strongly ordered (LL) shower formalism is anyway unpredictive. In Sect. 3.4 below, we show explicitly that the leading-logarithmic structure of smoothly ordered showers is identical to that of strongly ordered ones, but for the remainder of this section we constrain our attention to comparisons with fixed-order matrix elements.

A further point that must be addressed in the context of the ordering criterion is that our matrix-element-correction formalism, discussed below, requires a Markovian (history-independent) definition of the $\hat{t}$ variable in the $P_\mathrm {imp}$ factor in Eq. (68). Rather than using the scale of the preceding branching directly (which depends on the shower path and hence would be history-dependent), we therefore compute this scale in a Markovian way as follows: Given a *n*-parton state we determine the values of the evolution variable corresponding to all branchings the shower could have performed to get from any $(n-1)$- to the given *n*-parton state. The reference scale $\hat{t}$ is then taken as the minimum of those scales. The dead zone, equivalent to the unordered region, is now populated by allowing branchings of a restricted set of antennae to govern the full relevant phase space. Such antennae are called unordered, while other antennae are called ordered. It is in principle permissible to treat all antennae in an event as unordered. To mimic the structure of effective $2{\rightarrow }4$ and higher branchings, we, however, only tag those antennae which are connected to partons that partook in the branching that gave rise to the chosen value for $\hat{t}$ as unordered. Branchings of ordered antennae may then contribute below the scale $\hat{t}$.

For example, consider the case of a gluon emission being associated with the smallest value of the evolution variable. In this case the gluon as well as the two partons playing the role of the parent antenna that emitted the gluon, are marked for unordering and therefore all antennae in which these three partons participate are allowed to restart the evolution at their phase-space limits. This limited unordering reflects that no genuinely new region of phase space would be opened up by allowing partons/antennae completely unrelated to the “last branching” to be unordered, as these will already have explored their full accessible phase spaces during the prior evolution.

We note that for the final state the available phase space reduces for each successive branching, limiting the effect of the smooth ordering. In [40] it is shown that, for final-state radiation, the damping factor in Eq. (68) does not modify the LL 1 / *t* behaviour and only generates explicitly subleading $\hat{t}/t^2$ corrections in the strongly unordered limit, $t \gg \hat{t}$. For the initial state, the phase-space boundaries are governed by the hadronic centre-of-mass energy leading to possibly large unordered regions and therefore a rather large effect of the smooth ordering. As the main purpose of the smooth ordering is to fill all available phase space for the MECs, we restrict it to the ME corrected branchings by default and keep all following shower emissions strongly ordered. In this case, all damping factors get replaced by the MEC weight, see Sect. 3.6, by virtue of the Sudakov veto algorithm.

**Fig. 11:**
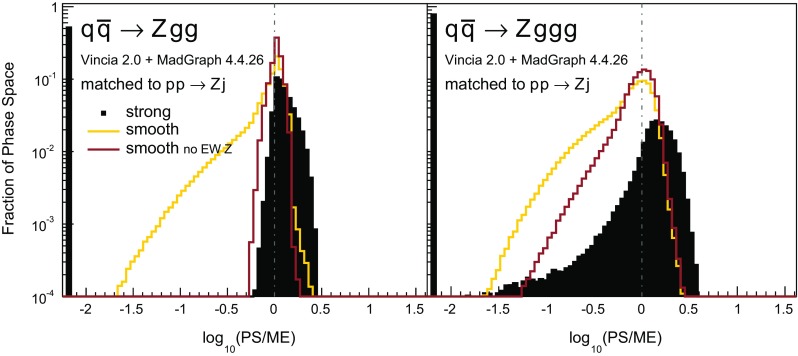
Antenna shower, compared to matrix elements: distribution of $\text {log}_{10}(\text {PS}/\text {ME})$ in a flat phase-space scan of the full phase space with strong and smooth ordering and smooth ordering with a cut on $m_{\perp \,Z}^2$. Contents normalised to the number of generated points. Gluon emission only

**Fig. 12:**
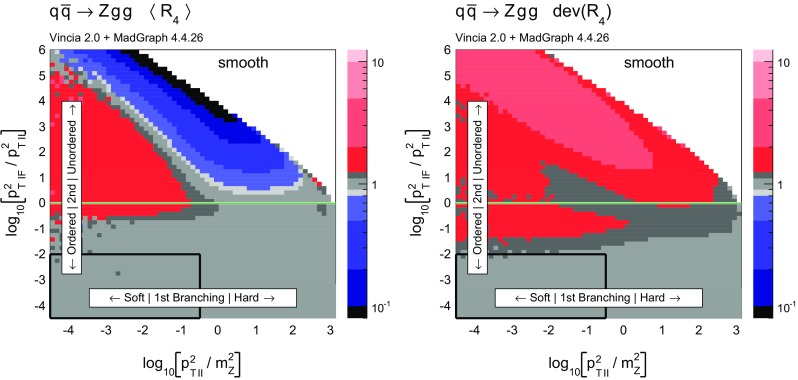
The value of $\langle R_4 \rangle$ (*left*) and $\text {dev}(R_4)$ (*right*), differentially over the 4-parton phase space, with $p_\perp ^2$ ratios characterising the first and second emissions on the *x* and *y* axis, respectively. Smooth ordering in the shower, with gluon emission only

We compare the logarithmic distributions of the ratio of the shower approximation to the matrix element for $q\bar{q}\rightarrow Zgg$ and $q\bar{q}\rightarrow Zggg$ for both strong and smooth ordering in Fig. 11. When applying smooth ordering, the distribution gets narrower on the side where the shower overcounts the tree-level matrix element, and that the dead-zone spike is replaced by an extended tail towards low ratios on the other side. This tail is due to configurations that look like a hard-QCD process accompanied with a radiated *Z*. Such phase-space points should in principle be populated by an electroweak shower, such as the one presented in [86]; not having developed the required formalism in the antenna context yet, however, we still allow our QCD shower to populate this region of phase space; it will in any case be corrected with matrix elements, see Sect. 3. To focus on the improvement in the QCD regions of phase space we apply a cut on the transverse mass of the *Z* boson and require it to be larger than the branching scale of the path that has been chosen to characterise the phase-space point,

73 $$\begin{aligned} p_{\perp \,\text {IF}}^2 < m_{\perp \,Z}^2=k_{\perp \,Z}^2+m_Z^2. \end{aligned}$$

We define $k_{\perp \,Z}^2$ to be the minimum of all possible

74 $$\begin{aligned} k_{\perp \,Z\,q}^2= \text {min}(E_Z^2,E_q^2)(1-\cos \theta _{Zq}). \end{aligned}$$

The resulting distributions are shown in red in Fig. 11. Applying the cut leads to a removal of the part of phase space where the *Z* should have been generated as an emission rather than as part of the hard process. The distribution is now dominated by QCD and the smoothly ordered shower produces a narrower as well as more symmetric distribution, compared to the strongly ordered shower.

**Fig. 13:**
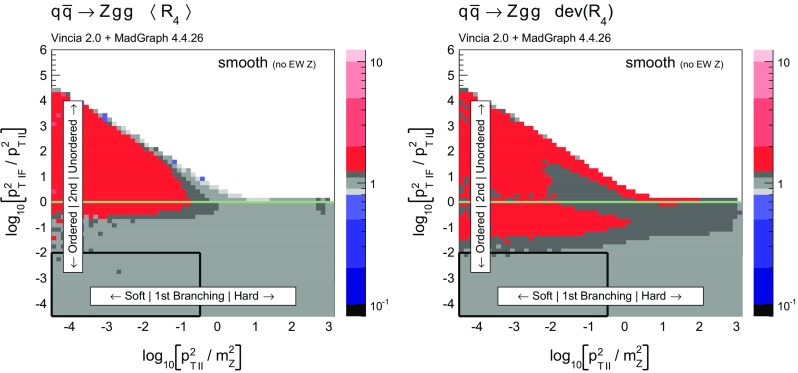
The value of $\langle R_4 \rangle$ (*left*) and $\text {dev}(R_4)$ (*right*), differentially over the 4-parton phase space, with $p_\perp ^2$ ratios characterizing the first and second emissions on the *x* and *y* axis, respectively. Smooth ordering in the shower, with a cut on $m_{\perp \,Z}^2$ and gluon emission only

Similarly we repeat the two-dimensional histograms for the smoothly ordered antenna shower in Fig. 12 without and in Fig. 13 with the cut on $m_{\perp \,Z}^2$. As expected, we obtain an improved description as compared to both the strong and unordered showers, Figs. 8 and 9 respectively. Due to the form of the improvement factor in Eq. (68) we get a factor of 0.5 at the green line, around where the scales of the two branchings coincide, leading to a better description already of this region. Once again these plots show that the shower undercounts the region where the *Z* boson is very soft and should have been generated with a weak shower, representing a path that is not available in Vincia  yet. The strongly unordered region remains somewhat overcounted, though by less than a factor 2, far better and with narrower distributions than was the case for the fully unordered shower, Fig. 9.

An extended set of plots, including Higgs production processes, can be found in Appendix B.

### Smooth ordering vs. strong ordering

**Fig. 14:**
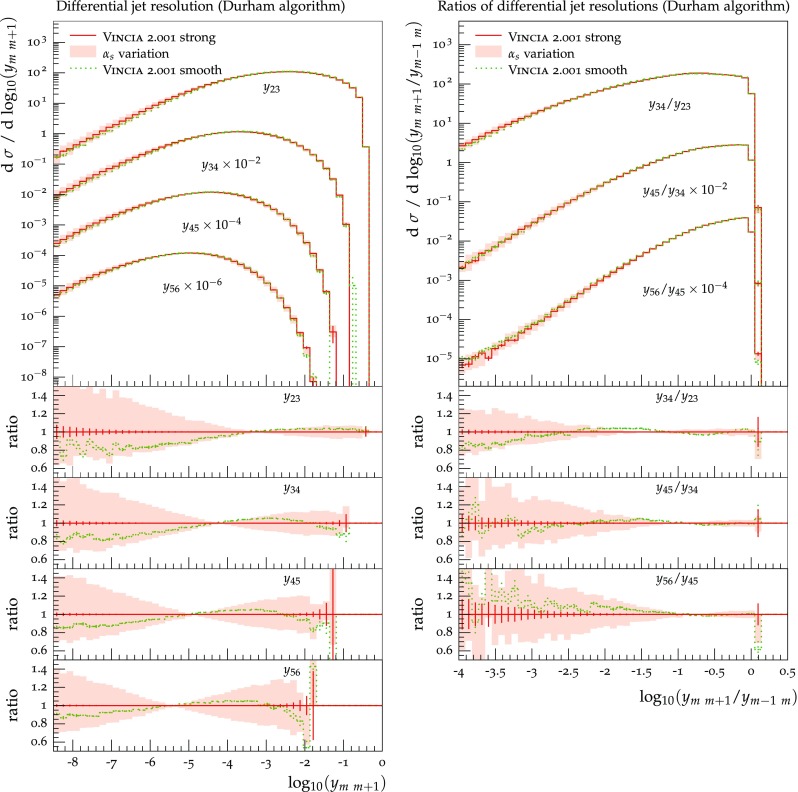
Logarithmic distributions of differential jet resolutions and their ratios for heavy *Z* decays ($m_Z=1000~\text {GeV}$). Predictions of Vincia  2.0 with strong (*smooth*) ordering are shown in *solid red* (*dotted green*) *lines*. The *red band* shows an $\alpha _s$ variation with $\alpha _s(m_Z)=0.12$ and $\alpha _s(m_Z)=0.14$

**Fig. 15:**
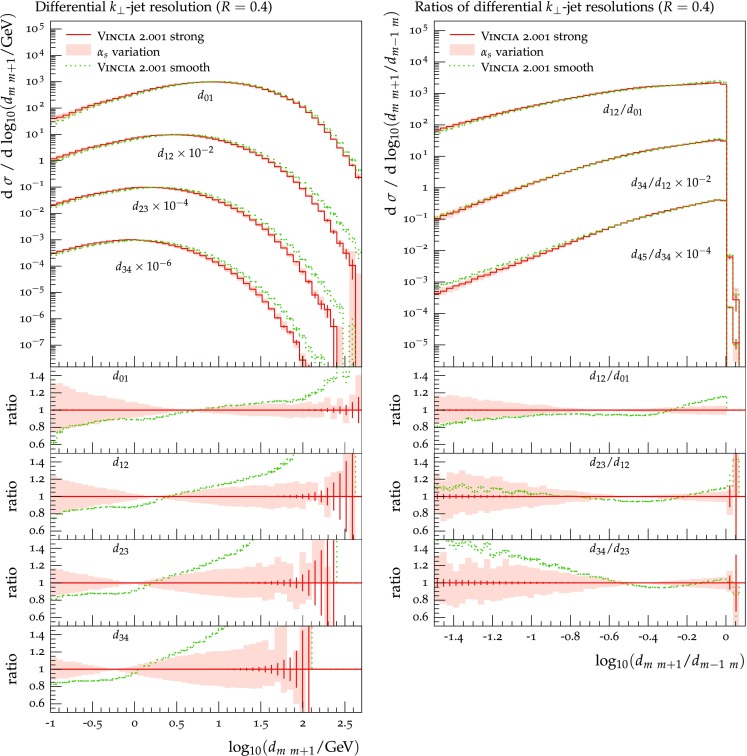
Logarithmic distributions of differential jet resolutions and their ratios for $Z+$jets events. Predictions of Vincia  2.0 with strong (*smooth*) ordering are shown in *solid red* (*dotted green*) *lines*. The *red band* shows an $\alpha _s$ variation with $\alpha _s(m_Z)=0.12$ and $\alpha _s(m_Z)=0.14$

This section presents a comparison of strong and smooth ordering, first in terms of their analytical leading-logarithmic structures, and then using jet clustering scales, investigating the processes $e^+e^-\rightarrow$ jets as well as $pp\rightarrow Z+$jets. The analyses are adapted from the code used in [30], originally written by Höche. In order to focus on the shower properties we present parton-level distributions, with MECs switched off, a fixed strong coupling with $\alpha _s(m_Z)=0.13$, and a very low cutoff, $10^{-3}~\text {GeV}$ for $e^+e^-\rightarrow$ jets and $10^{-2}~\text {GeV}$ for $pp\rightarrow Z+$jets. To furthermore put the magnitude of the differences between smooth and strong ordering into perspective, an $\alpha _s(m_Z)$-variation band for the strongly ordered result is included in Figs. 14 and 15.

We emphasise that, even leaving the $\alpha _s$ and cutoff settings aside, the distributions in this section are meant for validation only. The event generation modus used below does not make use of Vincia ’s matrix-element correction features. When using MECs, the main purpose of the smooth ordering is to fill the available phase space with non-vanishing weight, which allows a reweighting to reproduce the correct LO matrix-element result. Keeping this disclaimer in mind, it is still useful to investigate how the phase space is filled before MECs are applied.

#### Leading logarithms

As discussed in the preceding section, the leading (double-pole) behaviour of the gluon-emission antenna functions is just a constant over phase space when expressed in terms of the origami variables $\ln (p_\perp )$ and *y*. We begin by considering a conventional strongly ordered antenna shower, such as that of Ariadne [13, 21] (or Vincia  with strong ordering). The leading contribution to the Sudakov factor $\Delta (Q_\perp ^2, p_\perp ^2)$ representing the no-branching probability between two resolution scales $Q_\perp ^2 > p_\perp ^2$ (e.g., following a preceding branching which happened at the scale $Q_\perp$), is then, cf. Eq. (70),

75 $$\begin{aligned}- & {} \ln \Delta _\mathrm {strong} ~\mathop {\sim }\limits ^{\mathrm {LL}}~ \frac{\mathcal{C} \alpha _s}{2\pi } \int _{\ln p_\perp ^2}^{\ln Q_\perp ^2} \mathrm {d}\ln q_\perp ^2 \int _{-\ln (m/q_\perp )}^{\ln (m/q_\perp )} \mathrm {d}y \nonumber \\= & {} ~ \frac{\mathcal{C} \alpha _s}{2\pi } \int _{\ln p_\perp ^2}^{\ln Q_\perp ^2} \mathrm {d}\ln q_\perp ^2 \ln \left[ \frac{m^2}{q_\perp ^2}\right] \end{aligned}$$

76 $$\begin{aligned} = ~ \frac{\mathcal{C} \alpha _s}{2\pi } \left( \frac{1}{2} \ln ^2 \left[ \frac{Q_\perp ^2}{p_\perp ^2}\right] + \ln \left[ \frac{Q_\perp ^2}{p_\perp ^2}\right] \ln \left[ \frac{m^2}{Q_\perp ^2}\right] \right) , \end{aligned}$$

for a final–final antenna^11^ with invariant mass *m* and assuming $p_\perp ^2\ll m^2$. This agrees with the LL limit for dipole showers derived in [30]. We note that the second term is absent from [87, Eq. (8)] due to a phase-space restriction placed in Eq. (2) of that paper, which we believe is appropriate to remove double-counting of soft emissions in showers based on DGLAP kernels. In the context of antenna showers, however, the antenna functions already have the correct (eikonal) soft limits, and the imposition of this additional phase-space constraint would have the (undesired) effect of removing the added rapidity range corresponding to the extra origami fold discussed in Sect. 3.3, producing an “undercounting” of soft emissions. We therefore regard the expression above, Eq. (76), as the reference expression which an LL-correct antenna shower should reproduce.

A counter-example, illustrating an incorrect LL behaviour, can be furnished by considering a so-called “power shower” [73] in which the upper boundary of the integral above is replaced by $m^2$ rather than $Q_\perp ^2$ (e.g., letting newly created antennae evolve over their full phase spaces, irrespective of the ordering scale, and without any suppression). This produces an extra logarithm which is not present in the strongly ordered case:

77 $$\begin{aligned}&-\ln \Delta _{\mathrm {pwr}} \mathop {\sim }\limits ^{\mathrm {LL}} \frac{\mathcal{C} \alpha _s}{2\pi } \left( \frac{1}{2} \ln ^2 \left[ \frac{Q_\perp ^2}{p_\perp ^2}\right] \right. \nonumber \\&\quad +\left. \ln \left[ \frac{Q_\perp ^2}{p_\perp ^2}\right] \ln \left[ \frac{m^2}{Q_\perp ^2}\right] + {\frac{1}{2} \ln ^2 \left[ \frac{m^2}{Q_\perp ^2}\right] }\right) , \end{aligned}$$

where we have rewritten the $\frac{1}{2}\ln ^2(m^2/p_\perp ^2)$ result to make the two first terms identical to the ones produced in the strongly ordered case, so that the third term, highlighted in red, represents the difference.

For smooth ordering, with the $P_\mathrm {imp}$ suppression factor defined in Eq. (68), the relevant integral is

78 $$\begin{aligned} \int _{p_\perp ^2}^{m^2} \frac{1}{1+\frac{q_\perp ^2}{Q_\perp ^2}} \frac{\mathrm {d}q_\perp ^2}{q_\perp ^2} \ln \left[ \frac{ m^2}{q_\perp ^2}\right] , \end{aligned}$$

which after a bit of algebra can be cast in the following form:

79 $$\begin{aligned}&\frac{1}{2} \ln ^2\left[ \frac{Q_\perp ^2}{p_\perp ^2}\right] + \ln \left[ \frac{Q_\perp ^2}{p_\perp ^2}\right] \ln \left[ \frac{m^2}{Q_\perp ^2}\right] + \ln \left[ 1+\frac{p_\perp ^2}{Q_\perp ^2}\right] \ln \left[ \frac{m^2}{p_\perp ^2}\right] \nonumber \\&\quad - \mathrm {Li} _2\left[ \frac{-Q_\perp ^2}{m^2}\right] - \mathrm {Li} _2\left[ \frac{-p_\perp ^2}{Q_\perp ^2}\right] - \frac{\pi ^2}{6}, \end{aligned}$$

where the two first terms are again identical to those of Eq. (76). In the third term, $\ln (1+p_\perp ^2/Q_\perp ^2) \rightarrow 0$ for $p_\perp ^2/Q_\perp ^2 \rightarrow 0$, and the fourth and fifth terms are bounded by $-\pi ^2/12<\mathrm {Li} _2(-x)<0$ (with 0 corresponding to the limit $x \rightarrow 0$ and $-\pi ^2/12$ for $x \rightarrow 1$). We thus conclude that the LL properties of the antenna shower are not spoiled by changing from strong to smooth ordering.

#### Hadronic *Z* decays

To increase the available phase space we used a heavy *Z* with $m_Z=1000~\text {GeV}$ which decays hadronically. In Fig. 14 we present the parton-level result for four successive jet resolution measures, $y_{m\,m+1}$ (with $m\in \{2,3,4,5\}$), and their ratios $y_{m\,m+1}/y_{m-1\,m}$, using the Durham jet algorithm. Jet resolution scales exhibit a Sudakov suppression for low values, and exhibit fixed-order behaviour for large values. We note that in realistic calculations (and in experimental data), low-scale values are typically strongly affected by hadronisation corrections, which are absent here since we are at parton level, with a fixed $\alpha _s$. We also exclude values of $y_{m\,m+1}$ corresponding to scales below the shower cutoff. Small values of the ratios $y_{m\,m+1}/y_{m-1\,m}$ highlight the modelling in the region of large scale separation, i.e. where effects of resummation become relevant. Large values of $y_{m\,m+1}/y_{m-1\,m}$ are associated with the region of validity of fixed-order calculations.

In the distributions of the jet resolution scales themselves we observe moderate differences between the different ordering modes, up to $\mathcal {O}(20~\%)$. Smooth ordering generates more events with larger $y_{m\,m+1}$ separation and, consequently, fewer events with small separation, compared to strong ordering.

While the prediction with smooth ordering lies below the strongly ordered one for small values of the $y_{34}/y_{23}$ ratio, it eventually slightly exceeds the strong ordering in the $y_{56}/y_{45}$ ratio. This behaviour is a combination of two effects: Smooth ordering allows more phase-space coverage, while at the same time, the Markovian restart scale means that emissions from “ordered” antennae have more stringent phase-space restrictions than in the strongly ordered case. Thus, if more ordered antennae are present, which is only the case after several branchings, the Markovian restarting scale may lead to a softer multi-emission pattern than in the strongly ordered case. However, recall that MECs are an essential ingredient in the evolution, and that, for emissions beyond the highest ME multiplicity, no smooth ordering is applied. This means that, for lower multiplicities, the effect of smooth ordering is effectively removed and replaced by the full fixed-order result. For higher multiplicities, the shape change due to the Markovian restart scale is also absent, since smooth ordering is not applied. This suggests that smooth ordering of the entire cascade, and without MECs, exhibits some undesirable features. However, it is worth noting that the differences are largest in the soft region, where non-perturbative physics and tuning are expected to have large impact, as e.g. exemplified by a large dependence on the value of $\alpha _s(m_Z)$. Finally we note that the prediction with smooth ordering lie well within the $\alpha _s(m_Z)$-variation band of the strong ordering.

**Fig. 16:**
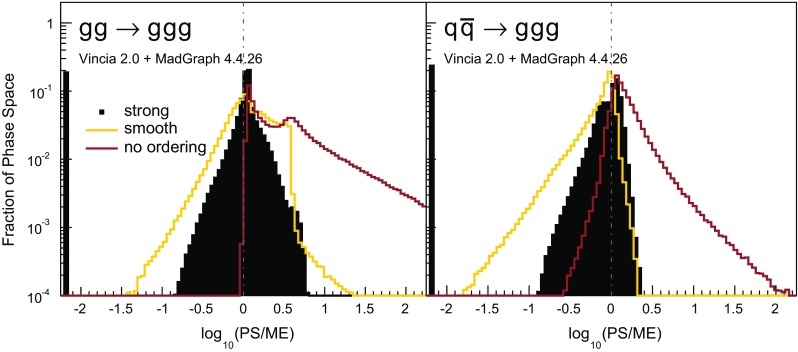
Antenna shower, compared to matrix elements: distribution of $\text {log}_{10}(\text {PS}/\text {ME})$ in a flat phase-space scan of the full phase space with strong, smooth, and no ordering with respect to the factorisation scale of the Born process. Contents normalised to the number of generated points. Gluon emission only

#### Drell–Yan

The parton-level results for $Z+$jets events are presented in Fig. 15: four successive jet resolution measures, $d_{m\,m+1}$ (with $m\in \{0,3\}$), and their ratios $d_{m\,m+1}/d_{m-1\,m}$, using the longitudinally invariant $k_\perp$ jet algorithm with $R=0.4$. As before, jet resolution scales show a fixed-order behaviour for large values, a Sudakov suppression and potentially large non-perturbative corrections for low values. The ratios $y_{m\,m+1}/y_{m-1\,m}$ are used to more clearly reveal the successive scale hierarchies.

The observations for both, the jet resolution scales, and their rations, are qualitatively similar to the $e^+e^-\rightarrow$ jets case, though quantitatively the effects here are larger. We notice the same turn-over when going from $d_{12}/d_{01}$ to $d_{34}/d_{23}$ we saw for *Z* decays, with the explanation being very similar to the case before. Smooth ordering will allow additional phase-space regions to be filled with harder emission (cf. Fig. 10). Due to the unitarity of the parton-shower algorithm, this naively means that fewer soft emissions occur. This is counter-acted by the Markovian restart scale, which means that the smoothly ordered shower yields softer emissions from “ordered” antennae. At low multiplicity, the former dominates, as all antennae are allowed to fill their available phase space, while at higher multiplicity, the latter drives the differences. Figure 15 shows trends in $d_{01}$ and $d_{12}$ similar to the ones visible in Figs. 10 and 20 of [87]. Note again that the additional, compensating effect of the Markovian restarting scale starts playing an important role for higher multiplicities.

### Hard jets in QCD processes

We already discussed our strategy to include hard branchings in non-QCD processes in Sect. 3.1. For processes with QCD jets in the final state we apply a different formalism, as the Born process already comes with a QCD scale. The first branching is allowed to populate all of phase space; however, the region with scales above the factorisation scale, $t>t_\text {fac}$, is treated with smooth ordering, as described in Sect. 3.3. In Fig. 16 we show the PS-to-ME ratios for $gg\rightarrow ggg$ and $q\bar{q}\rightarrow ggg$ where the factorisation scale is chosen to be the transverse momentum of the final-state partons in the Born $2\rightarrow 2$ process. We show a comparison of strong ordering, i.e. not including $t>t_\text {fac}$, smooth ordering with $\hat{t}=t_\text {fac}$ in the $P_\text {imp}$ factor, and no ordering, which corresponds to adding an event sample with $t>t_\text {fac}$. The plots indicate that the smooth ordering is preferred over adding hard jets as a separate event sample. Note that the asymmetric distribution of the PS-to-ME ratio for $gg\rightarrow ggg$ is the result of combining the distributions of different colour flows.

One could imagine applying the same treatment to non-QCD processes as well. However, this is not done in Vincia  as the factorisation scale in these processes is not a QCD scale and therefore not suited to enter the $P_\text {imp}$ factor.

### Matrix-element corrections with MadGraph 4

In this section we review the GKS procedure for iterative matrix-element corrections (MECs) [39]. To first order, the formalism is equivalent to that by Bengtsson and Sjöstrand in Refs. [5, 12], and to the approach used for real corrections in Powheg [88, 89]. In the context of final-state showers, the approach was generalised to multiple emissions in [39] where it was successfully used to include MECs through $\mathcal {O}(\alpha _s^4)$ for hadronic *Z* decays. A generalisation at the one-loop level has also been developed [40], though so far limited to $\mathcal {O}(\alpha _s^2)$. Here, we focus on tree-level corrections only.

Matrix-element corrections take the all-orders approximation of the shower as their starting point, and apply ME-based corrections to this structure order by order in perturbation theory. At tree level, the following multiplicative correction factor is applied to each antenna function for matching to leading-colour matrix elements,

80 $$\begin{aligned} \mathcal {C}_i\,{\bar{a}}_i~~\rightarrow ~~\mathcal {C}_i\,\bar{a}_i\,P_n^\text {ME}\quad \text {with} \quad P_n^\text {ME}=\frac{|\mathcal {M}_n|^2}{\sum _j \mathcal {C}_j\,{\bar{a}}_j\,|\mathcal {M}_{n-1}|^2}, \end{aligned}$$

with the *n*-particle matrix element squared $|\mathcal {M}_n|^2$, see Eqs. (81) and (82) for more details on colour ordering. Given Vincia ’s invertible kinematics maps and the explicit forms of the physical antenna functions defined in Sect. 2, the denominator is exactly calculable (taking the smooth ordering $P_\text {imp}$ factors defined in the previous section into account). The numerator is obtained by using amplitudes derived from MadGraph 4 [50], stored in Vincia ’s interfaces/MG4 subdirectory. Minor extensions were required to include processes with initial-state coloured partons, and several new matrix-element routines were added in the context of this work. The F77 syntax for calling a Vincia -modified MG4 matrix element is (using the specific example of a $b\bar{b}\rightarrow Hggg$ matrix element): 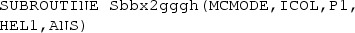 where

- INTEGER MCMODE selects between Leading Colour (0), Vincia  Colour (1), and Full Colour (2), as defined below,
- INTEGER ICOL selects which colour ordering is desired for MCMODE=0,1,
- DOUBLE PRECISION P1(0:3,NEXTERNAL) the momenta of the particles (in this example NEXTERNAL =6),
- INTEGER HEL1(4) holding up to 4 helicity configurations to be summed over, sufficient to average over an unpolarised initial 2-parton state or decaying vector boson, with specified final-state helicities. The enumeration of helicity configurations follows MadGraph’s normal helicity-counting convention.
- The requested matrix element squared is saved in the double-precision ANS variable, which in Vincia  always has only a single element.

From within Vincia   these matrix elements are accessed via C++ wrappers accessible via the VinciaPlugin:: mgInterface.ME2() methods, with definitions contained in the MG4interface.h and MG4interface.cc files. The input is a number of particles with partons being colour ordered, i.e. ordered in colour chains such as $q-g-g-\bar{q}$, where initial partons are crossed into the final state. The diagonal entry in MadGraph’s colour matrix, $\mathcal {C}^\text {MG}_{ii}$, associated with the given colour order, is chosen with ICOL. Using the more recent convention of MadGraph 5 [90]^12^ we define the leading-colour matrix element as

81 $$\begin{aligned} \left| \mathcal {M}_n\right| ^2 = \mathcal {C}^\text {MG}_{ii}~\left| \mathcal {J}_n^{(i)}\right| ^2, \end{aligned}$$

with the colour-stripped *n*-particle amplitude $\mathcal {J}_n^{(i)}$ corresponding directly to a JAMP in MadGraph’s nomenclature.

**Full-colour matrix-element corrections:** The full matrix element contains contributions that cannot be associated with a single colour ordering, i.e. the off-diagonal entries of the colour matrix, representing interferences between different colour orderings. To include those subleading-colour contributions while remaining within a formalism that provides strictly positive-definite correction factors, we use the following prescription [39] (Vincia  colour):

82 $$\begin{aligned} \left| \mathcal {M}_n\right| ^2&= \mathcal {C}^\text {MG}_{ii}~\left| \mathcal {J}_n^{(i)}\right| ^2 ~~\nonumber \\&\quad \rightarrow \mathcal {C}^\text {MG}_{ii}~\left| \mathcal {J}_n^{(i)}\right| ^2~ \frac{\sum _{j,k}\mathcal {C}^\text {MG}_{jk}~\mathcal {J}_n^{(j)}~\mathcal {J}_n^{(k)\,*}}{\sum _j \mathcal {C}^\text {MG}_{jj}~\left| \mathcal {J}_n^{(j)}\right| ^2}. \end{aligned}$$

The matrix element for each colour structure gets a correction from the subleading-colour part of the full matrix element that is proportional to the relative weight of that colour structure such that the sum over all colour flows reproduces the full-colour-summed matrix element norm squared.

Note that, though we show all matrix-element comparisons with leading colour, the conclusions do not change when replacing leading with full colour.

**Interference between different Born-level processes:** In previous versions of Vincia  the interference contributions from different Born-level processes were ignored; e.g., the interference between $Z\rightarrow d\bar{d}(g\rightarrow u\bar{u})$ and $Z\rightarrow u\bar{u}(g\rightarrow d\bar{d})$ contributing to $Z\rightarrow d\bar{d}u\bar{u}$ was not included. As those interferences can become fairly large and are already present for the first branching, e.g., $qg\rightarrow qgg$ can arise from $gg\rightarrow gg$ or $qg\rightarrow qg$ Born-level processes, we developed a more general formalism capable of handling these cases. Yet more interesting and illustrative are the interferences between $gg\rightarrow H$ and $Q\bar{Q}\rightarrow H$ Born processes, which both contribute to $Qg \rightarrow QH$ (with *Q* a heavy quark) but involve completely different types and orders of couplings. For this special case of Higgs production and decay we provide an option to allow/disallow such interferences.

**Fig. 17:**
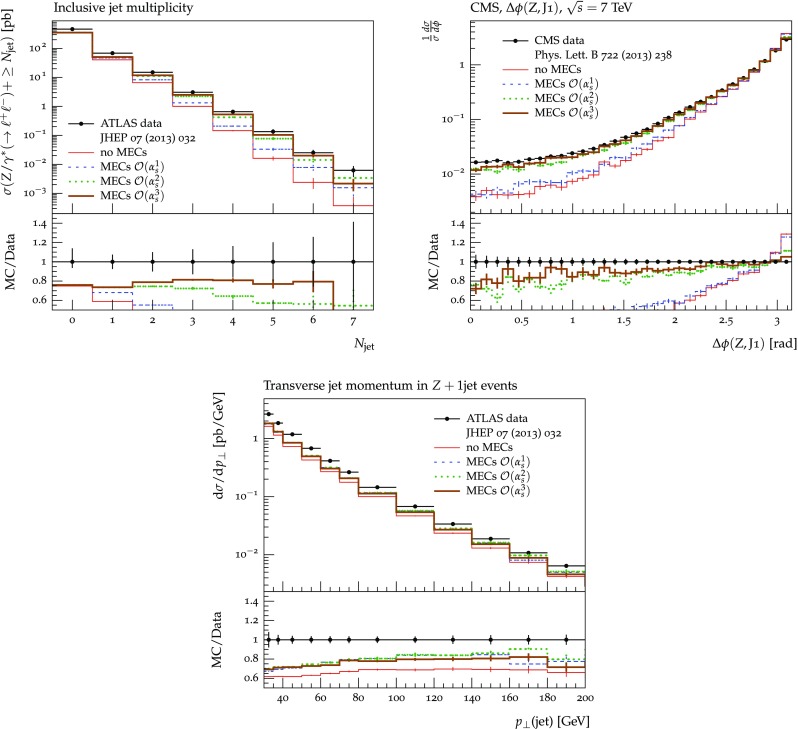
Inclusive cross section for the Drell–Yan lepton pair plus $\ge N$ jets (*top left*), distribution of the azimuthal angle between the *Z* boson and the hardest jet (*top right*), and jet $p_\perp$ in $Z+1\,\text {jet}$ events (*bottom*). Parton-level predictions of Vincia  2.0 for increasing order of MECs included, compared to ATLAS data from [91] and CMS data from [92]

**Impact of matrix-element corrections:** In Fig. 17 we show parton-level predictions of Vincia  in *Z* production events, i.e. multi-parton-interactions and hadronisation turned off, to focus solely on the shower properties and the impact of successive MECs. Comparisons to data including multi-parton-interactions and hadronisation will be presented in the section Sect. 4. We compare Vincia  with increasing orders of MECs included to ATLAS [91] and CMS [92] data. The inclusive cross section and the azimuthal angle between the reconstructed *Z* boson and the hardest jet (shown in the upper panel of Fig. 17) clearly highlight that MECs improve the description of data sensitive to multiple hard emissions. The progressive improvements that are introduced through iterated MECs is particularly obvious in the inclusive jet multiplicity. It is worthwhile mentioning that jet multiplicities beyond the third jet are only described by the approximate shower result. However, the combination of MECs up to third order seems to yield a good starting point for the shower, such that also high jet multiplicities are well described. Note that correcting only the hardest emission leads only to a modest improvement, since Vincia ’s antenna functions already provide a good approximation of the $Z+\text {jet}$ matrix element. The lower panel of Fig. 17 shows the jet transverse momentum in exclusive $Z+\text {jet}$ events. This observable should be dominated by the MEC of the hardest emission. Indeed, the description improves over plain showering, and is very stable upon iteratively including MECs to higher multiplicities. This showcases that MECs to higher-multiplicity states do not degrade the quality of the description of lower-multiplicity observables.

## Preliminary results and tuning

### The strong coupling

All components of Vincia  (i.e., both matrix-element corrections and showers) use a single reference value for strong coupling constant, with the default value $\alpha _s^{\overline{\mathrm {MS}}}(M_Z) = 0.118$, in agreement with the current world average [53, 93]. By default, we use two-loop running expressions, with the number of active flavours changing at each quark-mass threshold (including at $m_t$), though options for one-loop running or even fixed $\alpha _s$ values are provided as well. The inclusion of three-loop running effects is not relevant at the present (LO+LL) level of precision of the shower. In the infrared, the behaviour of $\alpha _s$ is regulated by allowing to evaluate it at a slightly displaced scale, $\alpha _s(\mu ) \rightarrow \alpha _s(\mu + \mu _0)$ and by imposing an upper bound $\alpha _s < \alpha _s^\mathrm {max}$. The set of default parameter values are:

+ Vincia:alphaSvalue = 0.118 ! Default alphaS(mZ) MSbar +

+ Vincia:alphaSorder = 2 ! Default is two-loop running +

+ Vincia:alphaSmuFreeze = 0.4 ! mu0 scale in alphaS argument, in GeV +

+ Vincia:alphaSmax = 1.2 ! max numerical value of alphaS +

Within the context of an LO+LL calculation, however, the value $\alpha _s(M_Z)=0.118$ produces a poor agreement with collider measurements; direct “tunings” at the LO+LL level typically find effective values closer to $\alpha _s(M_Z)=0.140$, see e.g. [39, 94]. To permit analogous tunings of Vincia , a user-specifiable prefactor is applied to the renormalisation-scale argument for each branching type,

83 $$\begin{aligned} \text{ Gluon } \text{ Emission } \text{: } \alpha _s(p_\perp )\rightarrow & {} \alpha _s(k_\mu \ p_\perp ),\end{aligned}$$

84 $$\begin{aligned} \text{ Gluon } \text{ Splitting } \text{: } \alpha _s(m_{qq})\rightarrow & {} \alpha _s(k_\mu ^{\mathrm {split}} \ m_{qq}), \end{aligned}$$

with equivalent parameters for splittings involving initial-state partons. The $k_\mu$ and $k_\mu ^{\mathrm {split}}$ parameters provide the same range of tuning possibilities for the effective coupling constant as in other parton-shower models, while they are simultaneously straightforward to interpret e.g. in the context of NLO matrix-element merging schemes.

The Vincia  shower algorithms do nonetheless incorporate a translation (on by default) between the $\overline{\mathrm {MS}}$ value given above and the so-called CMW (or MC) scheme which is appropriate for soft-gluon emission in coherent parton showers [32]. Since this translation is only rigorously defined in the limit of vanishing gluon energy, there is an ambiguity as to precisely how it should be applied to finite gluon energies. We address this by applying the CMW translation only to the coupling constant accompanying the eikonal (double-pole) term of the gluon-emission antenna functions,

85 $$\begin{aligned} \alpha _s^{\overline{\mathrm {MS}}} a_\mathrm {Emit}= & {} \alpha _s^{\overline{\mathrm {MS}}} \left( a_\mathrm {eik} + a_\mathrm {coll} + a_\mathrm {hard} \right) \end{aligned}$$

86 $$\begin{aligned}&\rightarrow \alpha _s^{\mathrm {CMW}} a_\mathrm {eik} \ + \ \alpha _s^{\overline{\mathrm {MS}}} \left( a_\mathrm {coll} + a_\mathrm {hard} \right) , \end{aligned}$$

with a few different options provided for how the eikonal term should be extrapolated to finite gluon energies. In a future study we shall aim to bring these ambiguities under better control by systematic application of one-loop corrected antenna functions, but this is still (far) beyond the scope of the present work.

### Vincia  2.0 default tune

Two main tools were used to perform the analyses: Vincia ’s own ROOT-based analysis tool, VinciaRoot [39], and Rivet [95]. For the hadron-collider distributions, we compare Vincia  2.0 with Pythia 8.2. For the $e^+e^-\rightarrow \mathrm {hadrons}$ analyses, we also include Vincia  1.2, since this version included NLO corrections to $e^+e^-\rightarrow 3\ \mathrm {jets}$ which have not yet been migrated to Vincia  2.0. Note, however, that even without the NLO corrections the two Vincia  versions are not exactly identical due to a slightly revised definition of the smooth-ordering criterion, to make it truly Markovian.

We note that these tunings were done manually (by “eye”), rather than by automated minimisation of $\chi ^2$ or equivalent measures. The latter is not as straightforward as it may sound, due to correlations between measurements and the influence of regions of low theoretical accuracy. These issues can be at least partially addressed by combining global knowledge and experience to (subjectively) choose binwise weighting factors. Nevertheless, manual and automated approaches may be considered complementary, with the former certainly competitive for the purpose of determining a set of “reasonable default values”, which is our principal aim here.

#### Hadronic *Z* decays

The final-state showering and hadronisation parameters are constrained using hadronic *Z* decays, mainly from the LEP experiments. In the context of Vincia  2.0, the rates of perturbative final-state branchings depend on the effective renormalisation scheme and scale choice, cf. Eq. (83), for which we have chosen the default values:

+ Vincia:CMWtypeFF = 2 ! CMW rescaling for FF antennae +

+ Vincia:alphaSkMuF = 0.6 ! muR prefactor for gluon emissions +

+ Vincia:alphaSkMuSplitF = 0.5 ! muR prefactor for gluon splittings +

                                                                    + ! (g -> qqbar)+

**Fig. 18:**
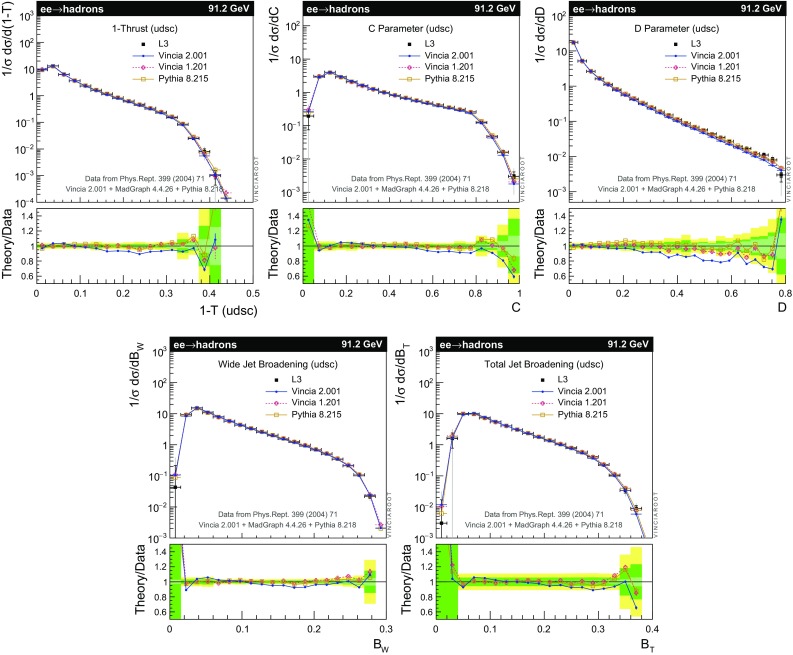
Event-shape variables compared with measurements performed by the L3 experiment

Figure 18 shows the event-shape observables^13^ that were used as the primary tuning constraints, compared with light-flavour tagged data from the L3 experiment [96]. In the main (top) plot panes, experimental data is represented by black square symbols, with 1-$\sigma$ and 2-$\sigma$ uncertainties represented by black vertical error bars and light-grey extensions, respectively. In the ratio panes, the inner (green) bands indicate the 1-$\sigma$ uncertainties on the data; outer (yellow) bands represent 2 $\sigma$.

Note that, since Vincia  2.0 does not incorporate the NLO corrections to $Z \rightarrow 3$ jets internally (unlike Vincia  1.2 [40]), we have chosen to allow the default tune to undershoot the reference data slightly in regions dominated by hard, resolved 3-jet events. This hopefully produces a more universal global tuning which should also be appropriate for use with the NLO merging strategies that are available within Pythia, notably UNLOPS [97].

The Lund string model [98–100] is used for hadronisation, with parameters (re)optimised for use with Vincia ’s shower model. The main parameters are the shower IR cutoff, the Lund fragmentation-function *a* and *b* parameters—which are defined by

87 $$\begin{aligned} f(z) \propto \frac{(1-z)^a}{z}\exp \left( \frac{-bm_\perp ^2}{z} \right) , \end{aligned}$$

with $z = E_\mathrm {hadron}/E_\mathrm {parton}$ and $m_\perp ^2 = m^2 + p_\perp ^2$—and the transverse-momentum broadening in string breaks, expressed as a Gaussian with width $\sigma _\perp \sim \mathcal{O}(\Lambda _\mathrm {QCD})$. The default Vincia  2.001 hadronisation-parameter values are,

+ Vincia:cutoffScaleFF = 0.9 ! Cutoff value in GeV for FF antennae +

+ StringZ:aLund = 0.5 ! Lund a parameter +

+ StringZ:bLund = 1.15 ! Lund b parameter +

+ StringZ:aExtraDiquark= 1.12 ! (extra for diquarks) +

+ StringPT:sigma = 0.295 ! Soft pT in string breaks +

**Fig. 19:**
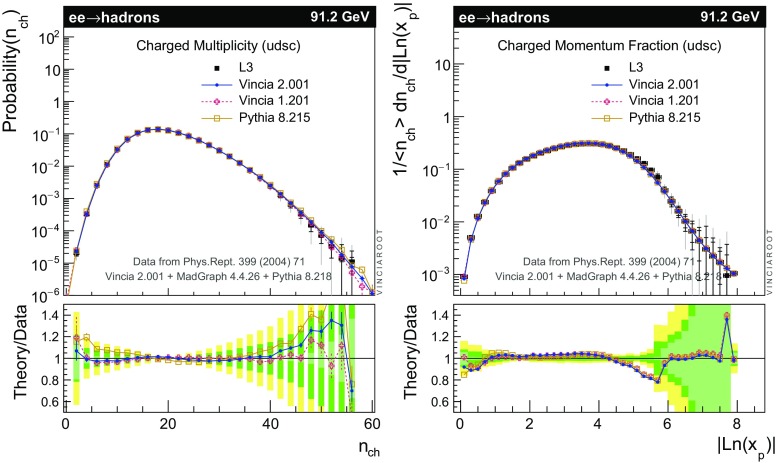
Charged-track multiplicity and momentum spectra, compared with measurements performed by the L3 experiment

The inclusive charged-particle multiplicity distribution and momentum ($x_p= 2|p|/E_\mathrm {cm}$) spectrum is shown in Fig. 19, again compared with light-flavour tagged L3 data from [96].

**Fig. 20:**
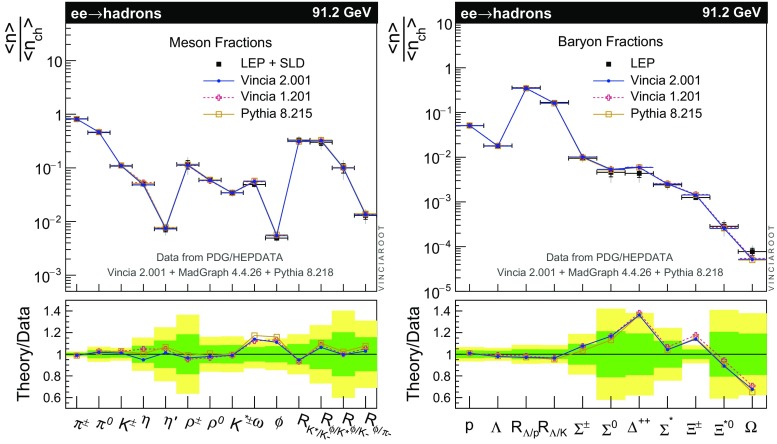
Identified-particle rates (expressed as fractions of the charged-particle multiplicity, or as indicated by *R* *symbols*), compared with the Monash 2013 reference values

Finally, we show the rates for identified light-flavour mesons and baryons in Fig. 20; these hardly change between the Pythia, Vincia  1, and Vincia  2 defaults. Note that we here compare to the reference measurement values derived for the Monash tune [94] of Pythia 8, which are not identical to the corresponding PDG values in particular for some of the baryon rates, see [94].

The corresponding full set of default parameter values are:

+ ! * String breakup flavour parameters +

+ StringFlav:probStoUD = 0.21 ! Strangeness-to-UD ratio +

+ StringFlav:mesonUDvector = 0.45 ! Light-flavour vector suppression +

+ StringFlav:mesonSvector = 0.555 ! Strange vector-meson suppression +

+ StringFlav:mesonCvector = 1.03 ! Charm vector-meson suppression +

+ StringFlav:mesonBvector = 2.2 ! Bottom vector-meson suppression +

+ StringFlav:probQQtoQ = 0.077 ! Diquark rate (for baryon production) +

+ StringFlav:probSQtoQQ = 1.0 ! Optional Strange diquark suppression +

+ StringFlav:probQQ1toQQ0 = 0.027 ! Vector diquark suppression +

+ StringFlav:etaSup = 0.53 ! Eta suppression +

+ StringFlav:etaPrimeSup = 0.105 ! Eta’ suppression +

+ StringFlav:decupletSup = 1.0 ! Optional Spin-3/2 Baryon Suppression +

+ StringFlav:popcornSpair = 0.9 ! Popcorn +

+ StringFlav:popcornSmeson = 0.5 ! Popcorn +

+ StringZ:rFactC = 1.60 ! Bowler parameter for c quarks +

+ StringZ:rFactB = 1.1 ! Bowler parameter for b quarks +

+ StringZ:useNonstandardB = true ! Special treatment for b quarks +

+ StringZ:aNonstandardB = 0.82 ! a parameter for b quarks +

+ StringZ:bNonstandardB = 1.4 ! b parameter for b quarks +

**Fig. 21:**
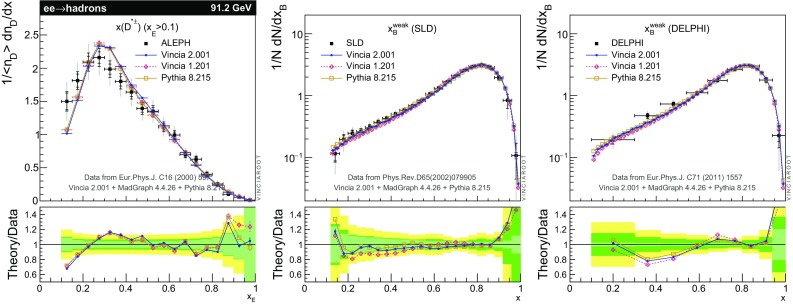
Distributions sensitive to heavy-quark fragmentation. *Left* the energy-fraction spectrum of charged $D^*$ mesons compared with ALEPH data [101]. *Center and right* the momentum-fraction spectrum of weakly decaying *B* hadrons compared to measurements by SLD [102] and DELPHI [103], respectively

Note that the last 6 parameters govern *c*- and particularly *b*-quark fragmentation. Since massive-quark effects are not explicitly addressed in this version of Vincia , these parameters have been chosen merely on a “best-effort” basis. We plan to return to this in a future update. A minimal set of checks on the level of agreement with heavy-quark spectra can be carried out using the vincia03-root and vincia05-root example programs included with the code. The former includes cross checks on the $g\rightarrow c\bar{c}$ and $g\rightarrow b\bar{b}$ rates as well as a $D^*$ spectrum, sensitive to *c*-quark fragmentation, while the latter focuses on constraints from *b*-tagged events. For completeness, the $D^*$ and *B*-hadron spectra produced by these example programs are reproduced in Fig. 21.

For the SLD $x_B$ spectrum, be advised that the current distribution of Vincia  (version 2.001) contains the spectrum obtained from the HepData archive [104] at the time of writing. However, the corrections contained in an erratum subsequently published by SLD [102] were missing from this table. The figure we show here contains the updated values (from the erratum). The updated table will be included in the next public release of Vincia , with corresponding updates expected in the HepData archive in due course.

**Fig. 22:**
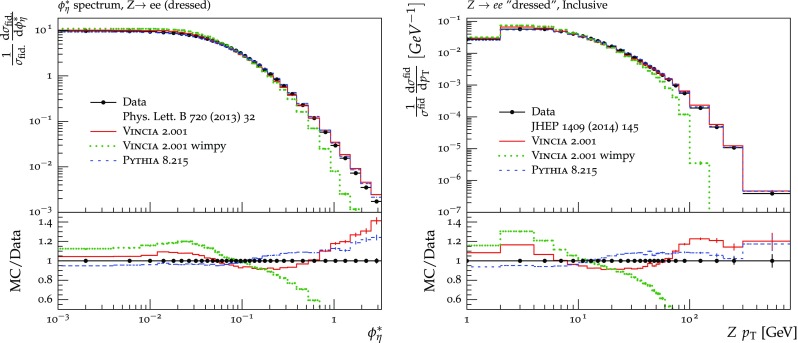
Angular correlations (*left*) and the transverse-momentum spectrum (*right*) of the Drell–Yan lepton pair. Predictions of default Vincia  2.0 in *red*, Vincia  2.0 wimpy in *green*, and Pythia 8.2 in *blue*, compared to ATLAS data from [105] and [106]

**Fig. 23:**
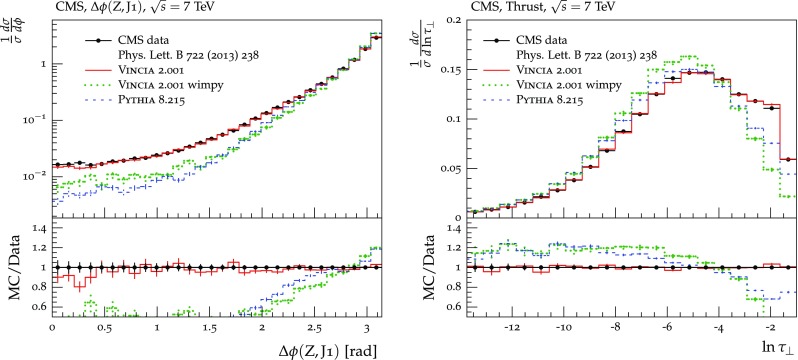
Distribution of the azimuthal angle between the *Z* boson and the hardest jet (*left*) and thrust (*right*). Predictions of default Vincia  2.0 in *red*, Vincia  2.0 wimpy in *green*, and Pythia 8.2 in *blue*, compared to CMS data from [92]

#### Drell–Yan

**Fig. 24:**
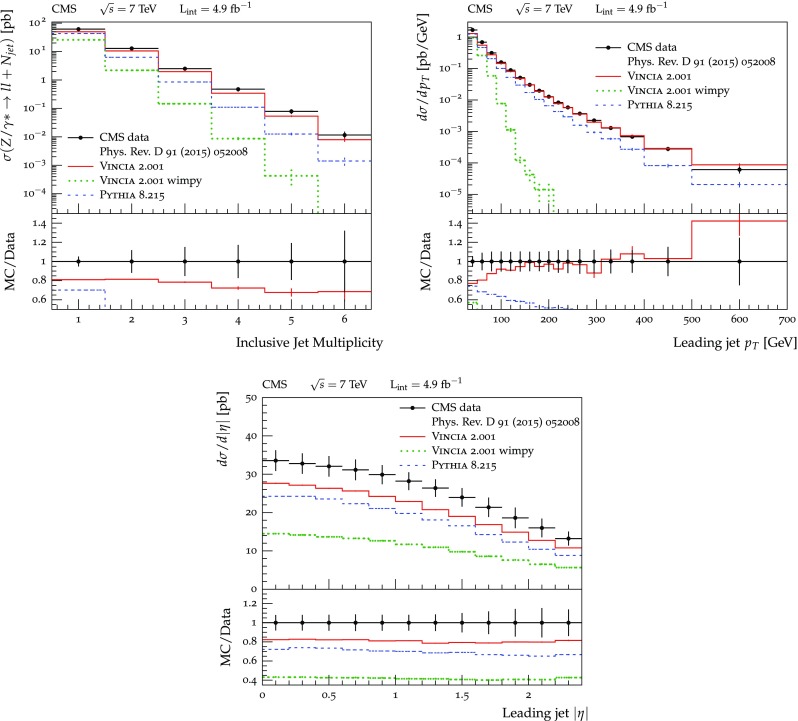
Inclusive cross section for the Drell–Yan lepton pair plus $\ge N$ jets (*top left*), the transverse-momentum (*top right*) and the pseudorapidity spectrum of the leading jet (*bottom*). Predictions of default Vincia  2.0 in *red*, Vincia  2.0 wimpy in *green*, and Pythia 8.2 in *blue*, compared to CMS data from [107]

In Figs. 22, 23 and 24 we show a set of observables in Drell–Yan events with ATLAS data from [105] and [106] and CMS data from [92] and [107]. We show predictions of default Vincia  2.0 in red, Vincia  2.0 wimpy (representing an ordinary shower, starting at the factorisation scale, i.e. no hard jets, no MECs, and strong ordering) in green, and Pythia 8.2 in blue. The Vincia  2.001 results correspond to the following default parameter choices:

#+ Perturbative shower parameters +

+ Vincia:CMWtypeII = 2 ! CMW rescaling of Lambda for II antennae +

+ Vincia:CMWtypeIF = 2 ! CMW rescaling of Lambda for IF antennae +

+ Vincia:alphaSkMuI = 0.75 ! Renormalisation-scale prefactor for ISR +

                                                                     + ! emissions +

+ Vincia:alphaSkMuSplitI = 0.7 ! -"- for g->qq splittings +

+ Vincia:alphaSkMuConv = 0.7 ! -"- for ISR conversions +

# + Shower IR cutoff and primordial kT +

+ Vincia:cutoffScaleII = 1.0 ! Cutoff value (in GeV) for II antennae +

+ Vincia:cutoffScaleIF = 0.9 ! Cutoff value (in GeV) for IF antennae +

+ BeamRemnants:primordialKThard = 1.05 ! Primordial kT for hard interactions +

+ BeamRemnants:primordialKTsoft = 0.7 ! Primordial kT for soft interactions +

Figure 22 shows angular correlations and the transverse-momentum spectrum of the Drell–Yan lepton pair. As one would expect the spectrum of Vincia  2.0 wimpy dies out at the *Z* mass. The prediction of default Vincia  2.0 shows too much activity in the hard tail of the spectrum which is caused by the reweighting of the event sample that includes high-$p_\perp$ jets, see Sect. 3.1. The tuning of the renormalisation-scale prefactors was chosen to produce as good a compromise as possible between the regions above and below $p_\perp \sim m_Z/2$.

Figure 23 shows the improved predictions when MECs are included. The left plot shows the relative azimuthal angle between the *Z* boson and the hardest jet, $\Delta \phi (\text {Z},\text {J}_1)$, where multiple shower emissions are required to obtain values below $\pi$. This plots shows that although Pythia’s power shower is matrix-element corrected for the first emission and results in a very good description of the *Z* transverse momentum, its prediction for $\Delta \phi (\text {Z},\text {J}_1)$ is worse than that of Vincia  2.0 wimpy. For this observable as well as for the thrust in the right plot in Fig. 23 default Vincia  2.0 agrees well with the data.

Figure 24 shows the inclusive cross section for the Drell–Yan lepton pair plus $\ge N$ jets, the transverse-momentum and the pseudorapidity spectrum of the leading jet. For all observables we find default Vincia  2.0 to produce a fairly good description of the data. As expected, Vincia  2.0 wimpy is not able to produce enough jets and cannot populate the full spectrum of the transverse momentum of the hardest jet.

**Fig. 25:**
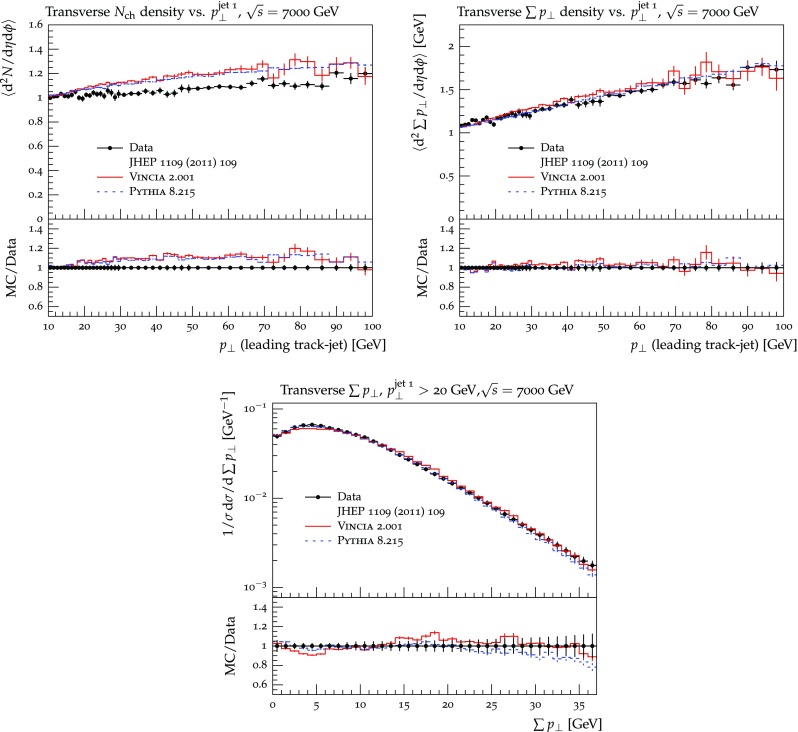
The underlying event in *pp* collisions at 7 TeV: Measurement of charged particles with $p_\perp > 0.5~\mathrm {GeV}$ and $|\eta |<2$ in the transverse region; average multiplicity (*top left*) and average scalar $\sum p_\perp$ (*top right*) as a function of the transverse momentum of the leading track-jet, and normalised scalar $\sum p_\perp$ distribution for leading track-jets with $p_\perp > 20~\mathrm {GeV}$ (*bottom*). Predictions of default Vincia  2.0 in *red* and Pythia 8.2 in *blue*, compared to CMS data from [110]. Note that we use a cut of $p_\perp > 15~\mathrm {GeV}$ in the hard process for the MC predictions and are therefore not showing the region of $1~\mathrm {GeV}<p_\perp ~\text {(leading track-jet)}<10~\mathrm {GeV}$ for the top histograms

#### Underlying event

Although soft-inclusive QCD physics is not the main focus of this version of Vincia , it is nonetheless relevant to verify that a reasonable description of the underlying event (UE) is obtained. We rely on the basic multi-parton-interaction (MPI) modelling of Pythia 8 [8, 34, 108] including its default colour-reconnection (CR) model, with parameters reoptimised for use with Vincia ’s initial- and final-state showers.

The MPI and CR parameter choices for the default Vincia  2.001 tune are as follows:

+ ! UE/MPI tuning parameters +

+ SigmaProcess:alphaSvalue = 0.118 +

+ SigmaProcess:alphaSorder = 2 +

+ MultiPartonInteractions:alphaSvalue = 0.119 +

+ MultiPartonInteractions:alphaSorder = 2 +

+ MultiPartonInteractions:pT0ref = 2.00 +

+ MultiPartonInteractions:expPow = 1.75 +

+ MultiPartonInteractions:ecmPow = 0.21 +

+ ! Parameters for PYTHIA 8’s baseline CR model +

+ ColourReconnection:reconnect = on +

+ ColourReconnection:range = 1.75 +

+ ! VINCIA is not compatible with perturbative diffraction +

+ Diffraction:mMinPert = 1000000.0 +

Note that we choose two-loop running for $\alpha _s$, analogously to the rest of Vincia , whereas the default Pythia 8.2 Monash tune [94] uses one-loop running. We also set the $\alpha _s(M_Z)$ reference value for hard processes (SigmaProcess: alphaSvalue) to the same value (0.118) as used for the showers, and use a similar value (0.119) for MPI, whereas the default Pythia tune employ larger values $\sim$ 0.13. The remaining MPI parameters were optimised using the 7-TeV charged-track summed-$p_\perp$ and number densities from [109], as well as their 900-GeV equivalents to constrain the energy-scaling parameter. The colour-reconnection strength was determined using the high-multiplicity region of the $\left<p_\perp \right>(N_\mathrm {ch})$ distribution measured by ATLAS [109] in minimum-bias events. It should be noted, however, that Vincia  is not suitable for (low-multiplicity) minimum-bias physics in its present form. This is partly related to the last parameter, which is included to switch off Pythia’s perturbative treatment of hard diffraction, with which Vincia  is not yet compatible.

**Fig. 26:**
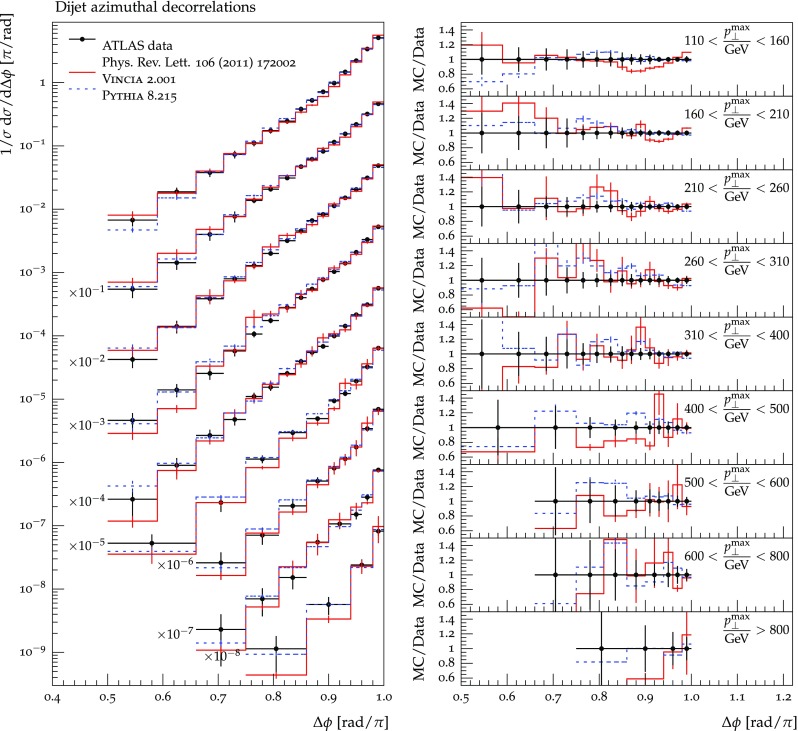
Distribution of dijet azimuthal decorrelations; predictions of Vincia  2.0 in *red* and Pythia 8.2 in *blue*, compared to ATLAS data from [112]

In Fig. 25, we compare default Vincia  2.0 with default Pythia 8.2, to three basic observables measuring the level of activity in the region transverse to the leading (hardest) charged-particle jet in the central pseudorapidity region, $|\eta |<2$, for LHC collisions at 7 TeV. We use the conventional definition of the transverse region, spanning $60^\circ< \Delta \phi < 120^\circ$ in azimuth with respect to the leading charged-particle jet, and compare to CMS data [110]. These comparisons satisfy us that at least the global properties of the UE are in acceptable agreement with the measurements, in particular in regards to the average $p_\perp$ density (top right-hand plot) and its event-to-event fluctuations (bottom right-hand plot). The charged-track multiplicity (top left-hand plot) is a more difficult observable to predict since it is less IR safe and hence more dependent on details of the hadronisation modelling; we presume that the small ($\mathcal{O}(10\,\%)$) discrepancies observed for both Pythia and Vincia  in this observables may be due to imperfections in Pythia’s still rather crude modelling of colour reconnections.

**Fig. 27:**
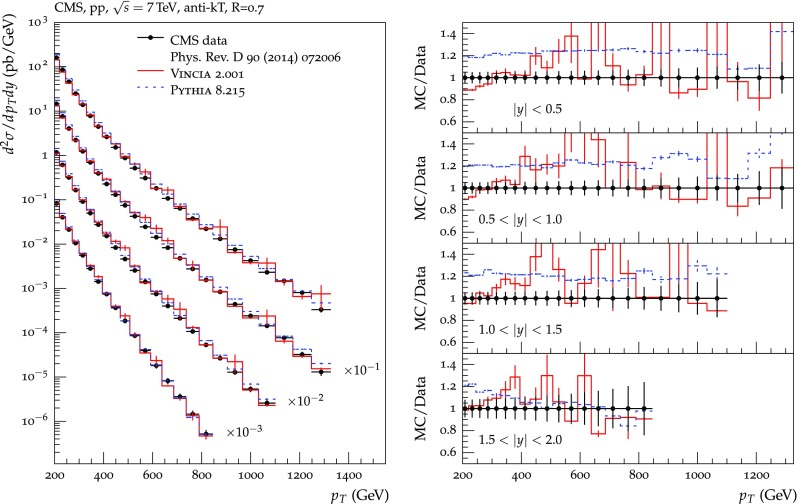
Inclusive jet cross section for 4 different rapidity bins as a function of the jet $p_\perp$. Predictions of Vincia  2.0 in *red* and Pythia 8.2 in *blue*. Data from CMS [113]

**Fig. 28:**
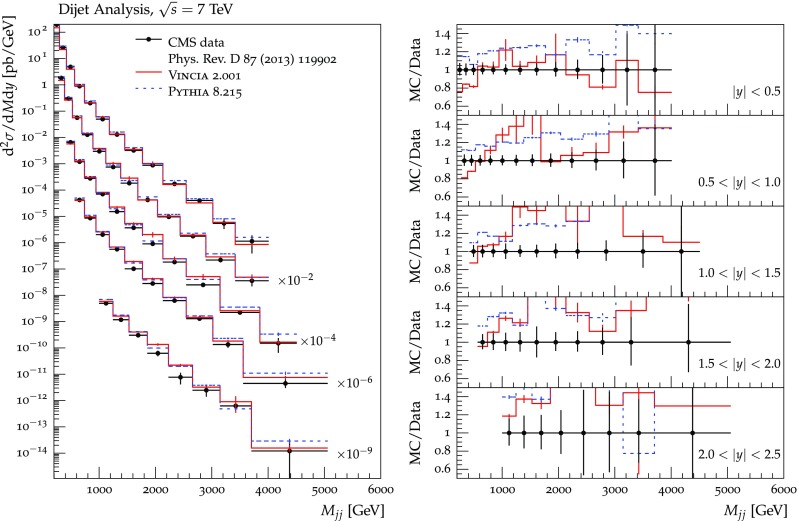
Inclusive dijet cross sections for 5 different rapidity bins as a function of the dijet mass. Predictions of Vincia  2.0 in *red* and Pythia 8.2 in *blue*. Data from CMS [114]

**Fig. 29:**
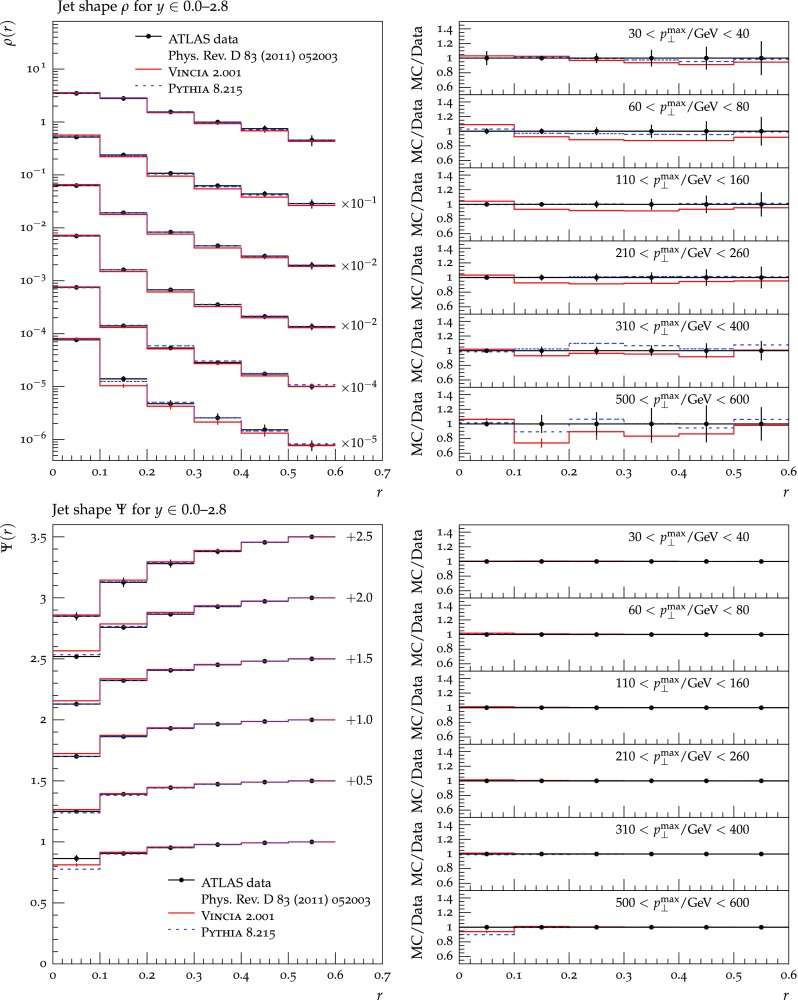
Distributions of the jet shape variables $\rho (r)$ (*top*) and $\Psi (r)$ (*bottom*) for different ranges of the jet transverse momentum. Predictions of Vincia  2.0 in *red* and Pythia 8.2 in *blue*, compared to ATLAS data from [115]

#### QCD jets

As our final set of validation checks, we consider the following observables in hard-QCD events: azimuthal dijet decorrelations, jet cross sections, and jet shapes. A technical aspect is that, due to the steeply falling nature of the jet $p_\perp$ spectrum, we use weighted events for all MC results in this section. The basic $2\rightarrow 2$ QCD process at the scale $\hat{p}_\perp$ is oversampled by an amount of $(\hat{p}_\perp /10)^4$, while the compensating event weight is $(10/\hat{p}_\perp )^4$. This allows one to fill the low-cross-section tails of the distributions with a reasonable amount of events. Note, however, that, for observables that are not identical to the biasing variable (which are all observables since no one-to-one measurement of the partonic $\hat{p}_\perp$ is possible), rare events with large weights can then produce “spurious” peaks or dips in distributions, accompanied by large error bars. Such features are to be expected in some of the distributions we show below; removing them would require generating substantially more events. While these features appear in the predictions of Vincia , they are not present in Pythia’s distributions. The reason is as follows: The aforementioned event weight becomes large for small values of $\hat{p}_\perp$. As this value serves as the starting scale in Pythia’s shower, the event will not produce any high-$p_\perp$ jets. In Vincia , however, the full phase space for the first emission is explored with the suppression factor $P_\text {imp}$ which is necessary for the application of MECs. In the rare cases, where Vincia  produces a jet with $p_{\perp \,j}\gg \hat{p}_\perp$, the large event weight becomes visible in distributions which require high-$p_\perp$ jets.

A second technical aspect is that, as shown in Fig. 16, the PS-to-ME ratios for QCD processes result in rather broad distributions already for the first-order correction with gluon emission only. This complicates including MECs for QCD processes, as violations in the Sudakov veto algorithm for generating emission and no-emission probabilities in the shower become more likely. By default, we neglect such violations. It is, however, possible for the user to check the effect of taking the violations into account properly via the procedure outlined in Ref. [111], which has been included in Vincia .

In Fig. 26 we show the predictions of Vincia  2.0 and Pythia 8.2 for dijet azimuthal decorrelations for different ranges of the jet transverse momentum and compare to ATLAS data from [112]. While we observe no glaring discrepancies with the data—the general trends of the distributions are well reproduced by both Vincia  and Pythia—there still appears to be some room for improvement, in particular with Vincia  undershooting the precisely measured data points around $\Delta \phi \sim 0.9$ in the lower two $p_\perp ^\text {max}$ bins by about 10–20 %.

Figures 27 and 28 show the transverse-momentum and jet mass spectra for different ranges of the jet rapidity and compare the MC predictions to CMS data from [113] and [114] respectively. We note that, whereas Pythia lies systematically above the data here, the lower default $\alpha _s$ value chosen in Vincia  causes the Vincia  normalisations to be substantially lower, even to the point of undershooting the measurements. This is not surprising given that the inclusive-jet cross section in Pythia/Vincia  is calculated at LO. The tails of the distributions unfortunately suffer from rather large weight-fluctuation effects, as was discussed above; nonetheless we note that the bins for which a reasonable statistical precision is obtained are generally closer to the data than the Pythia reference comparison.

Finally, in Fig. 29 we show the differential jet shape variable $\rho (r)$ and its cumulative integral $\Psi (r)$ for different ranges of the jet transverse momentum, compared with ATLAS data from [115]. This validates that the FSR broadening of QCD jets is in reasonable agreement with the experimental measurements, though we note that Vincia ’s distributions may be slightly too narrow, which we again regard as being consistent with the LL nature of Vincia ’s antenna functions and analogous to the slightly too narrow thrust distribution we allowed in the $e^+e^-$ event shapes. As far as a first default set of parameters goes, we are satisfied with this level of tuning, with future directions being informed both by lessons from combinations with external matrix-element matching and merging schemes and by attempts to integrate NLO antenna-function corrections into the shower itself, e.g. in the spirit of [40].

## Summary and conclusions

We presented the first publicly available antenna shower for initial and final state in Vincia  2.0, with focus on antenna functions and kinematic maps for initial-state radiation. Vincia  2.0 includes two different methods to explore the full phase space for the first emission, depending on the hard process at hand, without the disadvantages of a “power shower”. The full phase space of subsequent emissions is populated in a Markovian way. We compare explicitly to tree-level matrix elements for $pp\rightarrow Z/H\,jj(j)$ and $pp\rightarrow jjj$ to check the validity of our approximations.

We extended the iterative MEC approach to the initial state and include MECs for QCD up to $\mathcal {O}(\alpha _s^4)$ (4 jets), and for Drell–Yan and Higgs production up to $\mathcal {O}(\alpha _s^3)$ (*V* / *H* + 3 jets). This is the first time MECs beyond one leg have been applied to hadron collisions. However, this implementation was not without its complications; the large phase space available for initial-state branchings implies that “unordered” emissions account for a larger fraction of the full phase space than was the case for FSR, and the MEC factors are less well behaved and therefore more difficult / less efficient to implement, compared to pure final-state MECs. We also saw in Sect. 4.2 that biased event samples result in larger weight fluctuations for Vincia  than in the case of pure Pythia, presumably due to unordered emissions in Vincia  allowing a larger range of corrections to each event. In the context of future developments of Vincia , these aspects will therefore merit further consideration.

We presented first validation results with Vincia  2.0 for the main benchmark processes for FSR and ISR, including hadronic *Z* decays, Drell–Yan, and QCD jets. We observe good agreement with experimental data from the LEP/SLD and LHC experiments.

The development of a more highly automated interface to MadGraph 5 is among the main development targets for the near future. The feasibility of an interface to Njet2 [116] is also being explored.

## Figures and Tables

**Fig. 30:**
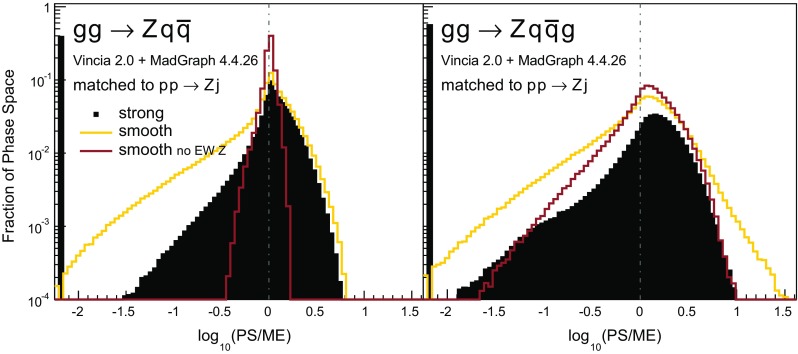
Antenna shower, compared to matrix elements: distribution of $\text {log}_{10}(\text {PS}/\text {ME})$ in a flat phase-space scan of the full phase space. Contents normalised to the number of generated points

**Fig. 31:**
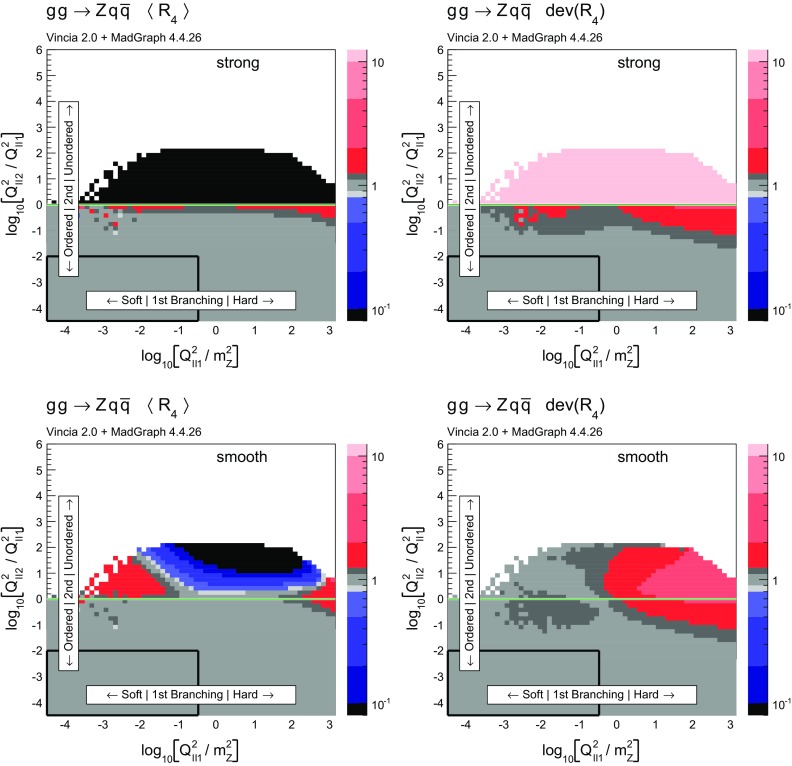
The value of $\langle R_4 \rangle$ (*left*) and $\text {dev}(R_4)$ (*right*), differentially over the 4-parton phase space, with $Q^2$ ratios characterising the first and second emissions on the *x* and *y* axis, respectively. Strong (*top*) and smooth (*bottom*) ordering in the shower

**Fig. 32:**
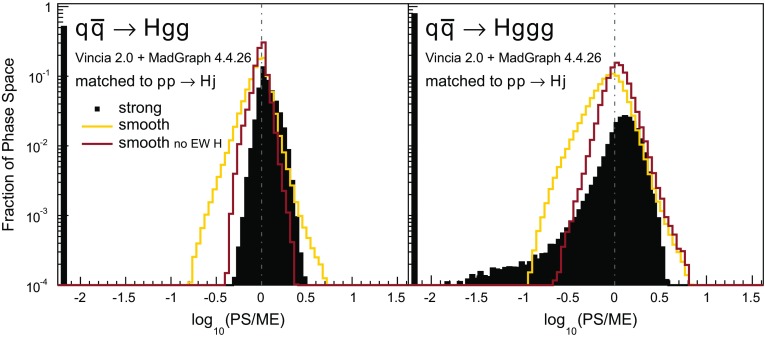
Antenna shower, compared to matrix elements: distribution of $\text {log}_{10}(\text {PS}/\text {ME})$ in a flat phase-space scan of the full phase space. Contents normalised to the number of generated points. Gluon emission only

**Fig. 33:**
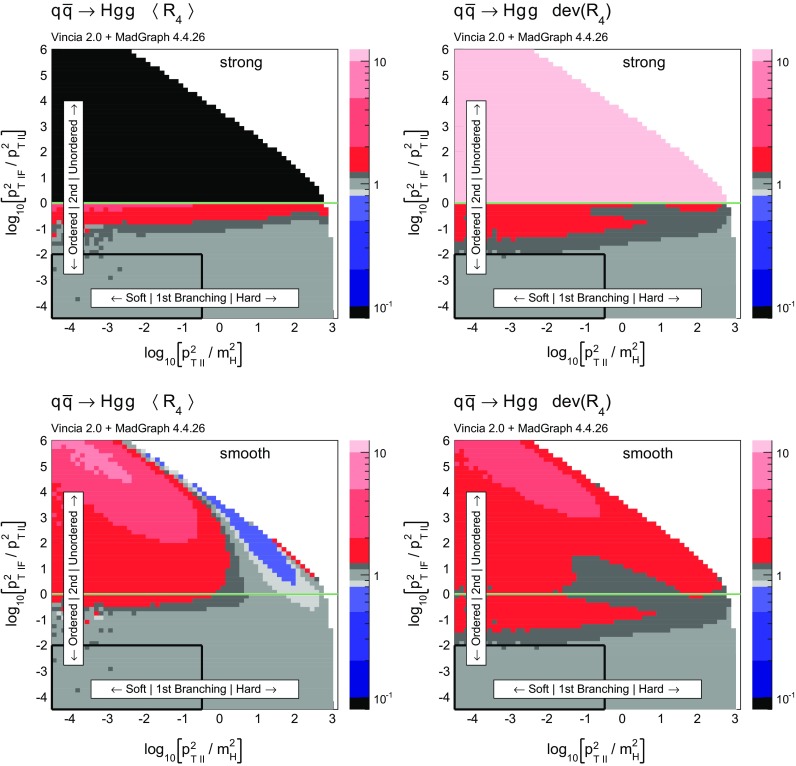
The value of $\langle R_4 \rangle$ (*left*) and $\text {dev}(R_4)$ (*right*), differentially over the 4-parton phase space, with $p_\perp ^2$ ratios characterising the first and second emissions on the *x* and *y* axis, respectively. Strong (*top*) and smooth (*bottom*) ordering in the shower, with gluon emission only

**Fig. 34:**
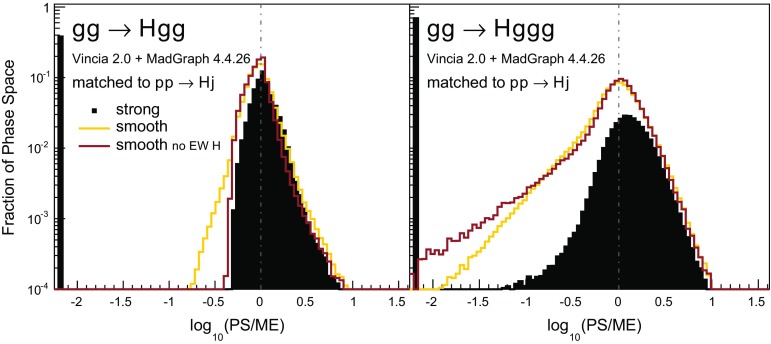
Antenna shower, compared to matrix elements: distribution of $\text {log}_{10}(\text {PS}/\text {ME})$ in a flat phase-space scan of the full phase space. Contents normalised to the number of generated points. Gluon emission only

**Fig. 35:**
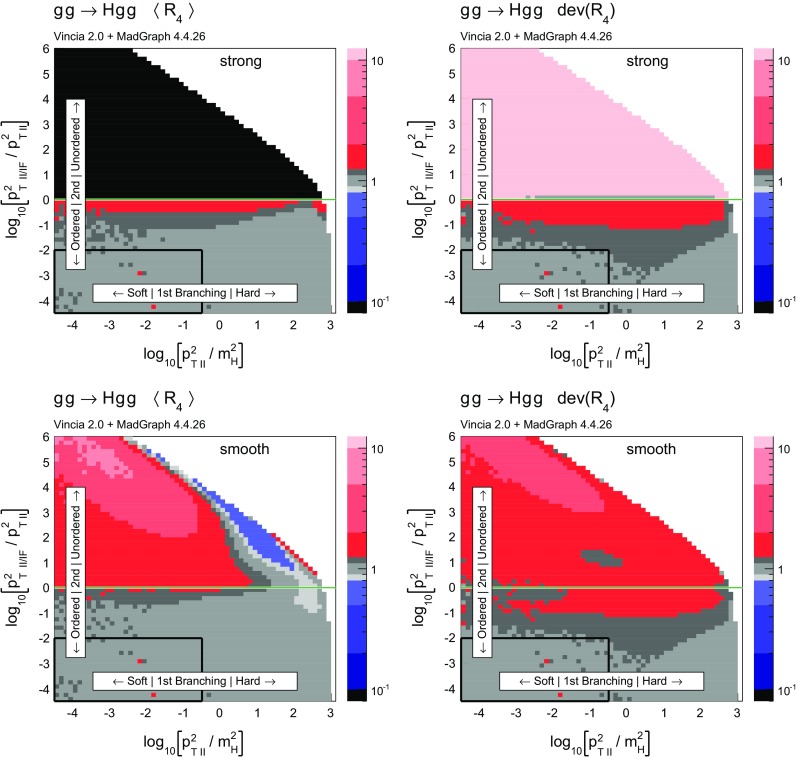
The value of $\langle R_4 \rangle$ (*left*) and $\text {dev}(R_4)$ (*right*), differentially over the 4-parton phase space, with $p_\perp ^2$ ratios characterising the first and second emissions on the *x* and *y* axis, respectively. Strong (*top*) and smooth (*bottom*) ordering in the shower, with gluon emission only

## Details of the shower algorithm

In this section we present some details of the shower algorithm, starting with the construction of the kinematics after the branching. Thereafter we will give a brief overview on how the antenna functions correctly reproduce the DGLAP functions in the collinear limit.

### Construction of the post-branching momenta

The antenna picture does not distinguish between the emitter/splitter and recoiler. Therefore, we have one mapping for each configuration, initial–initial, initia–final, and final–final. We express the momenta in terms of branching invariants only which leads to very simple expressions.

#### Initial–initial antennae

For a branching of type $AB\rightarrow abj$, the invariant mass and rapidity of the recoiler, $R\rightarrow r$, are not changed. The kinematics are constructed in the lab frame, where the post-branching momenta read as follows:

88 $$\begin{aligned} p_a^\mu= & {} \sqrt{\frac{s_{ab}}{s_{AB}}\frac{s_{AB}+s_{jb}}{s_{AB}+s_{aj}}}\,p_A^\mu , \end{aligned}$$

89 $$\begin{aligned} p_b^\mu= & {} \sqrt{\frac{s_{ab}}{s_{AB}}\frac{s_{AB}+s_{aj}}{s_{AB}+s_{jb}}}\,p_B^\mu , \end{aligned}$$

90 $$\begin{aligned} p_j^\mu= & {} \sqrt{\frac{s_{jb}^2}{s_{ab}s_{AB}}\frac{s_{AB}+s_{jb}}{s_{AB}+s_{aj}}}\,p_A^\mu \nonumber \\&+\, \sqrt{\frac{s_{aj}^2}{s_{ab}s_{AB}}\frac{s_{AB}+s_{aj}}{s_{AB}+s_{jb}}}\,p_B^\mu + \sqrt{\frac{s_{aj}s_{jb}}{s_{ab}}}\,p_\perp ^\mu , \end{aligned}$$

91 $$\begin{aligned} p_r^\mu= & {} p_a^\mu + p_b^\mu - p_j^\mu , \end{aligned}$$

with $p_\perp =(0,\cos \phi ,\sin \phi ,0)$, where $\phi$ is chosen uniformly in $[0,2\pi ]$.

#### Initial–final antennae

For a branching of type $AK\rightarrow akj$ the kinematics are constructed in the centre-of-mass frame of the parent antenna, which we define to be the rest frame of $p_A+p_K$ here, rotated so they are aligned with the *z* axis (the inverse of the corresponding Lorentz transformation is applied afterwards to bring the system back to the lab frame). The post-branching momenta read as follows:

92 $$\begin{aligned} p_a^\mu&= \frac{s_{AK}+s_{jk}}{s_{AK}}\,p_A^\mu , \end{aligned}$$

93 $$\begin{aligned} p_k^\mu&= \frac{s_{jk}s_{aj}}{s_{AK}(s_{AK}+s_{jk})}\,p_A^\mu + \frac{s_{ak}}{s_{AK}+s_{jk}}\,p_K^\mu \nonumber \\&\quad -\, \frac{\sqrt{s_{jk}s_{ak}s_{aj}}}{s_{AK}+s_{jk}}\,p_\perp ^\mu , \end{aligned}$$

94 $$\begin{aligned} p_j^\mu&= \frac{s_{jk}s_{ak}}{s_{AK}(s_{AK}+s_{jk})}\,p_A^\mu + \frac{s_{aj}}{s_{AK}+s_{jk}}\,p_K^\mu \nonumber \\&\quad +\, \frac{\sqrt{s_{jk}s_{ak}s_{aj}}}{s_{AK}+s_{jk}}\,p_\perp ^\mu , \end{aligned}$$

with $p_\perp$ defined as in the previous paragraph.

#### Final–final antennae

For a branching of type $IK\rightarrow ijk$ the kinematics are constructed in the centre-of-mass frame of the parent antenna, with the direction of parton *I* defining the positive *z* axis (The inverse of the corresponding Lorentz transformation is applied afterwards to bring the system back to the lab frame). A first set of post-branching momenta is constructed with parton *i* aligned with the *z* axis and using the *xz* plane to represent the branching plane,

95 $$\begin{aligned} p_i^\mu&= E_i(1,0,0,1), \end{aligned}$$

96 $$\begin{aligned} p_k^\mu&= E_k(1,\sin \theta _{ik},0,\cos \theta _{ik}), \end{aligned}$$

97 $$\begin{aligned} p_j^\mu&= E_j(1,-\sin \theta _{ij},0,\cos \theta _{ij}), \end{aligned}$$

with the energies

98 $$\begin{aligned} E_i = \frac{s_{IK}-s_{jk}}{2\,\sqrt{s_{IK}}},\quad E_k = \frac{s_{IK}-s_{ij}}{2\,\sqrt{s_{IK}}},\quad E_j = \frac{s_{IK}-s_{ik}}{2\,\sqrt{s_{IK}}}, \end{aligned}$$

and angles between the partons

99 $$\begin{aligned} \cos \theta _{ik} = \frac{2E_iE_k - s_{ik}}{2E_iE_k},\quad \cos \theta _{ij} = \frac{2E_iE_j - s_{ij}}{2E_iE_j}. \end{aligned}$$

The azimuth angle of the emitted gluon in the *xy* plane (defining the orientation of the branching plane) is generated by rotating the above momenta around the *z* axis by a uniformly chosen random angle $\phi$.

Finally, there remains one more global orientation angle, which can be cast as the angle between parton *i* and the original parton *I*, $\psi _{Ii}$, around an axis perpendicular to the branching plane (still in the centre-of-mass frame), i.e., specifying the degree to which $p_i$ is not aligned with the *z* axis after the branching. Different choices are implemented in Vincia (see [21, 23]), with the default being

$$\begin{aligned} \psi _{Ii}&= 1+\dfrac{2y_{ii}}{s_{IK}-s_{jk}}\quad \text {with}\\ y_{ii}&= -\frac{(1-\rho )s_{ik}/s_{IK}+2fs_{ij}s_{jk}/s_{IK}^2}{2(1-s_{ij}/s_{IK})},\\ f&= \frac{s_{jk}}{s_{ij}+s_{jk}},\quad \rho = \sqrt{1+4f(1-f)s_{ij}s_{jk}/s_{ik}s_{IK}}. \end{aligned}$$

The final post-branching momenta are constructed by rotating the *ijk* system by the angle $\psi _{Ii}$ around the axis perpendicular to the branching plane, and then finally performing the inverse Lorentz transform to bring the post-branching partons back to the lab frame.

### Collinear limits of the antenna functions

In this paragraph, we collect, for convenience of the reader, the collinear limits of the antenna functions used in Vincia . We also relate the antenna functions in this limit to corresponding DGLAP splitting kernels, which we will denote $P(x\rightarrow yz)$. Note that the apparent difference in colour factors for DGLAP splitting kernels and antenna functions is due to the phase-space and coupling factor, which, for antennae, is

100 $$\begin{aligned} \frac{\alpha _s\,\mathcal {C}_\text {ant}}{4\pi }\quad \text {with}\quad \mathcal {C}_\text {ant} \in [C_A,2C_F,T_R], \end{aligned}$$

whereas DGLAP kernels are conventionally defined with

101 $$\begin{aligned} \frac{\alpha _s\,\mathcal {C}_\text {DGLAP}}{2\pi }\quad \text {with}\quad \mathcal {C}_\text {DGLAP} \in [C_A,C_F,T_R/2]. \end{aligned}$$

We note that antenna functions with gluons as parent partons only reproduce half of a DGLAP kernel, as gluons take part in two antennae. In addition a factor of 1 / *z* will multiply the DGLAP kernels in the case of initial-state radiation.

The collinear limits of the antenna functions below agree with the limits found in Refs. [16–18, 118].

#### Initial–initial antennae

In the case of initial–initial antenna functions the energy-sharing variable is $z=s_{AB}/s_{ab}$ and we arbitrarily pick the invariant mass of one of the parton pairs, $Q^2=s_{aj}$, and its scaled version, $y=Q^2/s_{AB}$. For an easy comparison with the DGLAP kernels we rewrite the antenna functions in terms of these variables,

102 $$\begin{aligned} {\bar{a}}_{q\bar{q}\,g}^\text {II}&=~ \frac{1}{Q^2}\,\frac{1}{z}\,\left( 2\frac{z}{1-z-zy}+ \left( 1-z-zy\right) \right) +\mathcal {O}(Q^2), \end{aligned}$$

103 $$\begin{aligned} {\bar{a}}_{gg\,g}^\text {II}&=~ 2\,\frac{1}{Q^2}\,\frac{1}{z}\,\left( \frac{z}{1-z-zy}+\frac{1}{z}-1-y\right. \nonumber \\&\quad +\,\left. \left( 1-z-zy\right) \frac{z}{1+zy}\right) +\mathcal {O}(Q^2), \end{aligned}$$

104 $$\begin{aligned} {\bar{a}}_{qx\,q}^\text {II}&=~ \frac{1}{2}\,\frac{1}{Q^2}\,\frac{1}{z}\, \left( \frac{\left( 1-z-zy\right) ^2+1}{z}\right) , \end{aligned}$$

105 $$\begin{aligned} {\bar{a}}_{gx\,\bar{q}}^\text {II}&=~ \frac{1}{Q^2}\,\frac{1}{z}\,\left( 1-2z+2\frac{z^2}{1-zy}\right) . \end{aligned}$$

Note that we made use of $s_{jb}=s_{AB}(1-z-zy)/z$ and wrote the terms in the gluon-emission antennae that do not contain $1/Q^2$ singularities as $\mathcal {O}(Q^2)$, as they will vanish in the unresolved limit anyway.

Given this new form of the antenna functions, the collinear limits, $y\rightarrow 0$, are simple to read off,

106 $$\begin{aligned} {\bar{a}}_{q\bar{q}\,g}^\text {II}&\rightarrow ~ \frac{1}{Q^2}\,\frac{1}{z}\,\left( \frac{1+z^2}{1-z}\right) = \frac{1}{Q^2}\,\frac{1}{z}\,P(q\rightarrow qg),\end{aligned}$$

107 $$\begin{aligned} {\bar{a}}_{gg\,g}^\text {II}&\rightarrow ~ 2\,\frac{1}{Q^2}\,\frac{1}{z}\left( \frac{z}{1-z}+\frac{1-z}{z}+z(1-z)\right) \nonumber \\&= \frac{1}{Q^2}\,\frac{1}{z}\,P(g\rightarrow gg)\,\end{aligned}$$

108 $$\begin{aligned} \bar{a}_{qx\,q}^\text {II}&\rightarrow \frac{1}{2}\,\frac{1}{Q^2}\,\frac{1}{z}\,\left( \frac{(1-z)^2+1}{z}\right) = \frac{1}{2}\,\frac{1}{Q^2}\,\frac{1}{z}\,P(q\rightarrow gq), \end{aligned}$$

109 $$\begin{aligned} \bar{a}_{gx\,\bar{q}}^\text {II}&\rightarrow \frac{1}{Q^2}\,\frac{1}{z}\,\left( z^2+(1-z)^2\right) = \frac{1}{Q^2}\,\frac{1}{z}\,P(g\rightarrow q\bar{q}). \end{aligned}$$

Note in particular that the second and fourth antenna functions include the full DGLAP kernels for $g\rightarrow gg$ and $g\rightarrow q\bar{q}$, respectively. This is different from their final-state counterparts (see below) in which two neighbouring antenna functions must be summed over to recover the full DGLAP kernels. This difference arises from the fact that there is no “emission into the initial state”—the initial-state gluon only occurs as a hard leg, not as the emitted parton.

#### Initial–final antennae

We start with the collinear limit of the initial-state side, $Q^2=s_{aj}$ and $y=Q^2/s_{AK}$, and energy-sharing variable $z=s_{AK}/(s_{AK}+s_{jk})$ and rewrite the antenna functions,

110 $$\begin{aligned} {\bar{a}}_{qq\,g}^\text {IF}&=~ \frac{1}{Q^2}\,\frac{1}{z}\,\left( \frac{1+z^2-2zy}{1-z}\right) +\mathcal {O}(Q^2),\end{aligned}$$

111 $$\begin{aligned} {\bar{a}}_{gg\,g}^\text {IF}&= 2\,\frac{1}{Q^2}\,\frac{1}{z}\,\left( \frac{z(1-zy)}{1-z}+\frac{(1-z)(1-zy)}{z}\right. \nonumber \\&\left. \quad +\,z(1-z)\right) +\mathcal {O}(Q^2), \end{aligned}$$

112 $$\begin{aligned} {\bar{a}}_{qx\,q}^\text {IF}&=~ \frac{1}{2}\,\frac{1}{Q^2}\,\frac{1}{z}\,\left( \frac{(1-z)^2+(1-zy)^2}{z}\right) ,\end{aligned}$$

113 $$\begin{aligned} {\bar{a}}_{gx\,\bar{q}}^\text {IF}&=~ \frac{1}{Q^2}\,\frac{1}{z}\,\left( z(1-2z)y+z^2+(1-z)^2\right) . \end{aligned}$$

Note that we made use of $s_{jk}=s_{AK}(1-z)/z$ and $s_{ak}=s_{AK}(1-yz)/z$ and, as before, wrote the terms in the gluon-emission antennae that do not contain $1/Q^2$ singularities as $\mathcal {O}(Q^2)$.

Given this new form of the antenna functions, the collinear limits, $y\rightarrow 0$, are simple to read off,

114 $$\begin{aligned} {\bar{a}}_{qq\,g}^\text {IF}&\rightarrow \frac{1}{Q^2}\,\left( \frac{1+z^2}{1-z}\right) = \frac{1}{Q^2}\,\frac{1}{z}\,P(q\rightarrow qg),\end{aligned}$$

115 $$\begin{aligned} {\bar{a}}_{gg\,g}^\text {IF}&\rightarrow ~ 2\,\frac{1}{Q^2}\,\frac{1}{z}\,\left( \frac{z}{1-z}+\frac{1-z}{z}+z(1-z)\right) \nonumber \\&=\frac{1}{Q^2}\,\frac{1}{z}\,P(g\rightarrow gg)\, \end{aligned}$$

116 $$\begin{aligned} \bar{a}_{qx\,q}^\text {IF}&\rightarrow ~ \frac{1}{2}\,\frac{1}{Q^2}\,\frac{1}{z}\,\left( \frac{(1-z)^2+1}{z}\right) \nonumber \\&= \frac{1}{2}\,\frac{1}{Q^2}\,\frac{1}{z}\,P(q\rightarrow gq),\end{aligned}$$

117 $$\begin{aligned} {\bar{a}}_{gx\,\bar{q}}^\text {IF}&\rightarrow ~ \frac{1}{Q^2}\,\frac{1}{z}\,\left( z^2+(1-z)^2\right) = \frac{1}{Q^2}\,\frac{1}{z}\,P(g\rightarrow q\bar{q}). \end{aligned}$$

Now we continue with the collinear limit of the final-state side, $Q^2=s_{jk}$ and $y=Q^2/s_{AK}$, and energy-sharing variable $z=s_{ak}/s_{AK}$. The antenna functions, rewritten in terms of the new variables and using $s_{aj}=s_{AK}(1-z+y)$, read

118 $$\begin{aligned} \bar{a}_{qq\,g}^\text {IF}&=~ \frac{1}{Q^2}\,\frac{1}{z}\,\left( \frac{2z+(1-z+y)^2}{1-z+y}\right) +\mathcal {O}(Q^2),\end{aligned}$$

119 $$\begin{aligned} \bar{a}_{gg\,g}^\text {IF}&=~ \frac{1}{Q^2}\left( 2\,\frac{z}{1-z+y}+z(1-z)\right) +\mathcal {O}(Q^2), \end{aligned}$$

120 $$\begin{aligned} \bar{a}_{xq\,\bar{q}}^\text {IF}&=~ \frac{1}{2}\,\frac{1}{Q^2}\left( (1-z+y)^2+z^2\right) . \end{aligned}$$

Given this new form of the antenna functions, the collinear limits, $y\rightarrow 0$, are simple to read off,

121 $$\begin{aligned} \bar{a}_{qq\,g}^\text {IF}&\rightarrow ~ \frac{1}{Q^2}\,\left( \frac{1+z^2}{1-z}\right) \qquad = \frac{1}{Q^2}\,P(q\rightarrow qg),\end{aligned}$$

122 $$\begin{aligned} \bar{a}_{gg\,g}^\text {IF}&+ \bar{a}_{gg\,g}^\text {IF} [z\leftrightarrow 1-z] \rightarrow ~ 2\,\frac{1}{Q^2}\,\nonumber \\&\times \,\left( \frac{z}{1-z}+\frac{1-z}{z}+z(1-z)\right) \qquad = \frac{1}{Q^2}\,P(g\rightarrow gg)\, \end{aligned}$$

123 $$\begin{aligned} \bar{a}_{xq\,\bar{q}}^\text {IF}&\rightarrow ~ \frac{1}{2}\,\frac{1}{Q^2}\,\left( (1-z)^2+z^2\right) \qquad = \frac{1}{2}\,\frac{1}{Q^2}\,P(g\rightarrow q\bar{q}). \end{aligned}$$

#### Final–final antennae

In the case of final–final antenna functions the energy-sharing variable is $z=s_{ik}/s_{IK}$ and we arbitrarily pick the invariant mass of one of the parton pairs, $Q^2=s_{jk}$, and its scaled version, $y=Q^2/s_{IK}$. For an easy comparison with the DGLAP kernels we rewrite the antenna functions in terms of these variables (leaving out the finite parts as their choice is arbitrary),

124 $$\begin{aligned} \bar{a}_{q\bar{q}\,g}^\text {FF}&=~ \frac{1}{Q^2}\,\left( \frac{2z+(1-z-y)^2}{1-z-y}\right) +\mathcal {O}(Q^2),\end{aligned}$$

125 $$\begin{aligned} \bar{a}_{gg\,g}^\text {FF}&= \frac{1}{Q^2}\left( 2\,\frac{z}{1-z-y}+(1-z-y)(z+y)\right) +\mathcal {O}(Q^2),\end{aligned}$$

126 $$\begin{aligned} {\bar{a}}_{xq\,\bar{q}}^\text {FF}&=~ \frac{1}{2}\,\frac{1}{Q^2}\left( (1-z-y)^2+z^2\right) . \end{aligned}$$

Note that we made use of $s_{ij}=s_{IK}(1-z-y)$ and, as before, wrote the terms in the gluon-emission antennae that do not contain $1/Q^2$ singularities as $\mathcal {O}(Q^2)$.

Given this new form of the antenna functions, the collinear limits, $y\rightarrow 0$, are simple to read off,

127 $$\begin{aligned}&\bar{a}_{q\bar{q}\,g}^\text {FF} \rightarrow ~ \frac{1}{Q^2}\,\left( \frac{1+z^2}{1-z}\right) \qquad = \frac{1}{Q^2}\,P(q\rightarrow qg),\end{aligned}$$

128 $$\begin{aligned}&\bar{a}_{gg\,g}^\text {FF} + \bar{a}_{gg\,g}^\text {FF} [z\leftrightarrow 1-z] \rightarrow 2\,\frac{1}{Q^2}\,\nonumber \\&\times \left( \frac{z}{1-z}+\frac{1-z}{z}+z(1-z)\right) = \frac{1}{Q^2}\,P(g\rightarrow gg)\, \end{aligned}$$

129 $$\begin{aligned}&\bar{a}_{xq\,\bar{q}}^\text {FF} \rightarrow ~ \frac{1}{2}\,\frac{1}{Q^2}\,\left( (1-z)^2+z^2\right) \quad = \frac{1}{2}\,\frac{1}{Q^2}\,P(g\rightarrow q\bar{q}). \end{aligned}$$

**Table 1:** Definitions of the evolution variable *t* and the complementary phase-space variable $\zeta$ for II, IF and FF configurations, with the $\zeta$ boundaries in the last two columns

	Evolution	Definition	$\zeta$ Boundaries	
	Variable *t*	of $\zeta$	$\zeta _\text {min}$	$\zeta _\text {max}$
II	$\frac{s_{aj}s_{jb}}{s_{ab}}$	$\frac{s_{aj}}{s_{ab}}$	$\frac{s-s_{AB}}{s}-\sqrt{\frac{t_\text {max}-t}{s}}$	$\frac{s-s_{AB}}{s}+\sqrt{\frac{t_\text {max}-t}{s}}$
		$\frac{s_{aj}}{s_{AB}}$	$\frac{s-s_{AB}}{2s_{AB}}-\frac{1}{s_{AB}}\sqrt{s(t_\text {max}-t)}$	$\frac{s-s_{AB}}{2s_{AB}}+\frac{1}{s_{AB}}\sqrt{s(t_\text {max}-t)}$
		$\frac{s_{jb}}{s_{AB}}$	$\frac{s-s_{AB}}{2s_{AB}}-\frac{1}{s_{AB}}\sqrt{s(t_\text {max}-t)}$	$\frac{s-s_{AB}}{2s_{AB}}+\frac{1}{s_{AB}}\sqrt{s(t_\text {max}-t)}$
	$s_{aj}$	$\frac{s_{ab}}{s_{AB}}$	$\frac{s_{AB}+t}{s_{AB}}$	$\frac{s}{s_{AB}}$
	$s_{jb}$	$\frac{s_{ab}}{s_{AB}}$	$\frac{s_{AB}+t}{s_{AB}}$	$\frac{s}{s_{AB}}$
IF	$\frac{s_{aj}s_{jk}}{s_{AK}+s_{jk}}$	$\frac{s_{jk}+s_{AK}}{s_{AK}}$	$\frac{s_{AK}+t}{s_{AK}}$	$\frac{1}{x_A}$
		$\frac{s_{aj}}{s_{AK}+s_{jk}}$	$\frac{t~x_A}{s_{AK}(1-x_A)}$	1
		$\frac{s_{aj}}{s_{jk}}$	$\frac{t~x_A}{s_{AK}(1-x_A)^2}$	$\frac{s_{AK}+t}{t}$
	$s_{aj}$	$\frac{s_{jk}+s_{AK}}{s_{AK}}$	$\text {max}\left( 1,\frac{t}{s_{AK}}\right)$	$\frac{1}{x_A}$
	$s_{jk}$	$\frac{s_{aj}}{s_{AK}+s_{jk}}$	0	1
FF	$4\,\frac{s_{ij}s_{jk}}{s_{IK}}$	$\frac{s_{ij}}{s_{ij}+s_{jk}}$	$\frac{1}{2}\left( 1-\sqrt{1-\frac{t}{s_{IK}}}\right)$	$\frac{1}{2}\left( 1+\sqrt{1-\frac{t}{s_{IK}}}\right)$
	$s_{ij}$	$\frac{s_{jk}}{s_{IK}}$	0	$\frac{s_{IK}-t}{s_{IK}}$
	$s_{jk}$	$\frac{s_{ij}}{s_{IK}}$	0	$\frac{s_{IK}-t}{s_{IK}}$

### Phase-space variables and limits

In Table 1 we give an overview on combinations of the evolution variable *t* and complementary phase-space variable $\zeta$ that are used in the shower.

## Comparison with matrix elements

In this section we show an extended set of plots where we compare the shower approximation to leading-order matrix elements; see Sects. 3.2 and 3.3 for a description of the observables. We show the one- and two-dimensional distributions of the PS-to-ME ratios for $gg\rightarrow Zq\bar{q}(g)$ in Figs. 30 and 31, for $q\bar{q}\rightarrow Hgg(g)$ in Figs. 32 and 33, and for $gg\rightarrow Hgg(g)$ in Figs. 34 and 35. As before, see Eqs. (73) and (74), we include distributions with a cut on the transverse mass of the boson, $m_{\perp \,Z}^2$ (labelled “no EW Z”) and $m_{\perp \,H}^2$ (labelled “no EW H”), respectively.

## Footnotes

1 Dokshitzer–Gribov–Lipatov–Altarelli–Parisi. We mourn the recent passing of Guido Altarelli (1941–2015), a founder of this field and a great inspirer.

2 Note that a “Catani–Seymour” dipole [19, 20] corresponds roughly speaking to a partial-fractioning of an antenna or Lund colour dipole into two pieces.

3 Older parton-shower models often treat initial-state (ISR) and final-state (FSR) evolution in disjoint sequences. In this case, it is challenging to ensure that FSR evolution from the enlarged and changed parton ensemble after ISR evolution recovers the coherent features. Implementations of a combined simultaneous evolution chain for ISR and FSR may also be challenging. The current $p_\perp$-ordered showers in Pythia 8 [8, 34, 35] do, for example, not account for the coherence structure of the hardest gluon emission in $t\bar{t}$ events [36]. In contrast, we suspect that due to the fact that physical output states (parsed through hadronisation) are only constructed at the end of the evolution, the angular-ordered algorithms of Herwig [6] and Herwig++ [7] produce coherent sequences of emission angles in IF configurations correctly. This assessment relies on the assumption that the algorithms ensure that the angular constraints on final-state emission variables are unchanged by the ISR shower evolution, and vice versa.

4 E.g., in our treatment the case illustrated by [49, Fig. 19b] cannot occur: $Z\rightarrow qgg\bar{q}$ with the two gluons collinear to each other, non-collinear to any of the quarks, and in a singlet state.

5 Giele–Kosower–Skands [39].

6 The earliest Vincia  paper on final–final antennae [23] used an alternative convention: $\hat{a} + \hat{b} \rightarrow a + r + b$.

7 The resulting evolution equation will contain objects that are very close to the unintegrated parton densities of [56, 57].

8 Up to and including Vincia  2.001, these variations were erroneously applied by multiplying or dividing the antenna functions by $(1+\alpha _s)$, which is degenerate with the renormalisation-scale variations.

9 The value of merging scales is typically well below $t_\text {fac}$.

10 This is trivial for $q\bar{q}\rightarrow Zgg$ as the corresponding antenna function already is the ratio of the LO matrix elements.

11 For initial–initial antennae, replace *m* in the phase-space limit on the rapidity integral in Eq. (75) by $\sqrt{s}=\sqrt{s_{AB}/(x_A x_B)}$, assuming $x_Ax_B \ll 1$. For initial–final antennae, replace it by $\sqrt{s_{AK}/x_A}$ assuming $x_A\ll 1$.

12 In MadGraph 4 the colour matrix for amplitudes with multiple quark pairs is more complicated and required a decomposition by hand to separate the leading- from the subleading-colour parts, as is now done automatically by MadGraph 5.

13 For definitions, see e.g. [96].
